# Rapid Discovery of Antifungal α-Pyrone Analogs from *Diaporthe kyushuensis* ZMU-48-1 via an HSQC-Based DeepSAT Strategy

**DOI:** 10.3390/jof12030161

**Published:** 2026-02-25

**Authors:** Siwen Yuan, Jiaqi Zheng, Yueling Wu, Xijing Wang, Haiwen Wang, Min Yan, Tianpeng Yin

**Affiliations:** School of Bioengineering, Zunyi Medical University, Zhuhai 519041, China; zhengjiaqi@zmuzh.edu.cn (J.Z.); wuyuelin@zmuzh.edu.cn (Y.W.); wangxijing@zmuzh.edu.cn (X.W.); wanghaiwen@zmuzh.edu.cn (H.W.); yanmin@zmuzh.edu.cn (M.Y.)

**Keywords:** DeepSAT, α-pyrone, antifungal, *Diaporthe kyushuensis*

## Abstract

Endophytic fungi represent an extensive source of chemically diverse and bioactive polyketides. Herein, an HSQC-based DeepSAT-guided strategy was employed for the scaffold-prioritized isolation of metabolites from *Diaporthe kyushuensis* ZMU-48-1, an endophyte isolated from *Acacia confusa*. DeepSAT analysis of the semi-purified fractions identified an α-pyrone chemotype, which facilitated the targeted isolation of thirteen pyranone-type polyketides, including eight previously undescribed analogues, diaporthopyranone A–H (**1**–**8**). The structures of the new compounds were rigorously elucidated via comprehensive spectroscopic analysis (1D and 2D NMR and HRESIMS), while their absolute configurations were established by comparing experimental and TDDFT-calculated ECD data. All isolates (**1**–**13**) were screened for antifungal activity against nine plant pathogens. While most metabolites were inactive at 200 μg/mL, compound 4 inhibited *Alternaria alternata* and *Valsa mali* (MICs = 100 and 80 μg/mL, respectively), and compounds **6** and **8** exhibited selective inhibition against *Colletotrichum musae* (MICs = 60 and 80 μg/mL). These results expand the pyranone repertoire of the genus *Diaporthe* and underscore the utility of the HSQC–DeepSAT platform for the streamlined discovery of specific natural product scaffolds.

## 1. Introduction

Fungal diseases of crops pose a persistent threat to global food security, resulting in substantial yield losses and compromising the quality of agricultural products. The emergence of resistant plant pathogenic strains, driven by the prolonged application of a limited set of commercial fungicides, necessitates the continuous discovery of novel antifungal lead structures with diverse modes of action [1]. Endophytic fungi have increasingly been recognized as a prolific and under-explored repository of structurally unique polyketides, many of which exhibit significant agrochemical potential [2,3]. Among these, species of the genus *Diaporthe* are notable for their ability to produce a diverse array of secondary metabolites, particularly polyketide-derived lactones and oxygenated heterocycles with potent antimicrobial and phytotoxic properties [4,5].

α-Pyrones (2*H*-pyran-2-one derivatives) represent a prominent class of simple yet versatile polyketide scaffolds characterized by a six-membered unsaturated lactone ring. These metabolites are widely distributed across the fungal and plant kingdoms, often serving as key biosynthetic intermediates or end-products with broad-spectrum biological activities, including antifungal, antibacterial, and cytotoxic effects [6,7]. Despite their structural simplicity, the -pyrone framework provides a scaffold for extensive functionalization through hydroxylation, methylation, and side-chain elongation, leading to a rich chemical space. Although several pyranones have been isolated from the genus *Diaporthe* [8,9,10], their structural diversity within this genus remains incompletely mapped, and their specific roles as antifungal agents against specialized plant pathogens are only partially characterized.

The traditional “isolate-then-characterize” workflow for discovering such metabolites is often hindered by the presence of complex metabolic profiles and the repetitive isolation of known compounds. To address these challenges, Nuclear Magnetic Resonance (NMR)-based metabolite profiling has emerged as a robust tool for the rapid dereplication and prioritization of extracts. Specifically, Heteronuclear Single Quantum Coherence (HSQC)-guided analysis allows for the organization of semi-purified fractions based on chemical class and scaffold characteristics. The development of DeepSAT—a deep learning-based system trained to predict chemical fingerprints and scaffold classes directly from HSQC spectra—has revolutionized the annotation of dominant chemotypes in complex mixtures [11]. By enabling scaffold-level prioritization prior to exhaustive purification, DeepSAT streamlines the discovery process, allowing researchers to focus on specific, high-interest chemical families such as α-pyrones within diverse fungal extracts.

**Figure 1:**
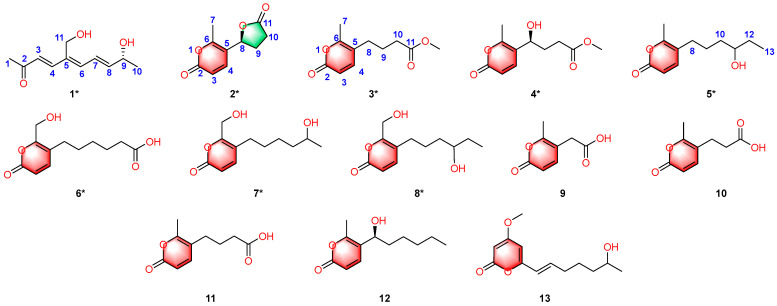
Structures of compounds **1**–**13** (* denotes new compounds).

In the present study, we describe a structure-directed investigation of the endophytic fungus *Diaporthe kyushuensis* ZMU-48-1, isolated from *Acacia confusa* [12]. Utilizing an HSQC-based DeepSAT strategy, we successfully prioritized fractions exhibiting an α-pyrone dominated chemotype, which directed the subsequent targeted isolation. This approach afforded thirteen pyranone derivatives, including eight previously undescribed analogues, diaporthopyranone A–H (**1**–**8**). (Figure 1). Herein, we report the DeepSAT-assisted isolation, the rigorous structural elucidation of the new metabolites via extensive spectroscopic analysis and Time-Dependent Density Functional Theory-Electronic Circular Dichroism (TDDFT-ECD) calculations, and the evaluation of their antifungal activities against a panel of nine economically significant plant pathogens (*Fusarium graminearum*, *Botryosphaeria dothidea*, *Fusarium oxysporum*, *Colletotrichum gloeosporioides*, *Valsa mali*, *Colletotrichum musae*, *Alternaria alternata*, *Colletotrichum agenarium*, and *Bipolaris sorokiniana*).

## 2. Materials and Methods

### 2.1. General Experimental Procedure

1D and 2D NMR spectra were recorded on a Bruker AVANCE III 600 spectrometer (Bruker, Karlsruhe, Germany) equipped with a 5 mm cryoprobe (CPP BBO600S3 BB-H&F-D-05 Z XT). The deuterated solvents CDCl_3_ and CD_3_OD were purchased from Cambridge Isotope Laboratories (Tewksbury, MA, USA). High-resolution electrospray ionization mass spectra (HR-ESI-MS) were acquired on an Orbitrap Fusion Lumos mass spectrometer (Thermo Fisher Scientific, Waltham, MA, USA). Infrared (IR) spectra were recorded on an Agilent Cary 630 FT-IR spectrometer (Agilent, Santa Clara, CA, USA). Optical rotations were measured on an MCP 200 polarimeter (Anton Paar, Ryde, Austria).

Preparative high-performance liquid chromatography (HPLC) was carried out on a Waters system (Waters, Milford, MA, USA) comprising a 1525 binary gradient pump and a 2998 photodiode-array detector. Separations were performed on Xtimate C18 (10 × 250 mm, 5 μm; Welch Materials, Shanghai, China), Ultimate XB-Phenyl (10 × 250 mm, 5 μm; Welch Materials, Stamford, CT, USA), and ACE C18 (4.6 × 250 mm, 5 μm; Advanced Chromatography Technology, Aberdeen, UK) columns. HPLC-grade acetonitrile and methanol were obtained from Tianjin Concord Technology Co., Ltd. (Tianjin, China), and chromatograms were monitored at 220, 254, 275, and 310 nm.

Column chromatography (CC) was performed on silica gel (300–400 mesh; Qingdao Haiyang Chemical Factory, Qingdao, China). Thin-layer chromatography (TLC) was conducted on GF_254_ plates from the same manufacturer. The spots were visualized under UV light and/or by spraying with vanillin–sulfuric acid reagent followed by heating. Analytical-grade methanol (MeOH), dichloromethane (DCM), petroleum ether (PE), ethyl acetate (EtOAc), and ethanol (EtOH) were purchased from Tianjin Damao Chemical Reagent Co., Ltd. (Tianjin, China).

### 2.2. Fungal Material and Identification

The fungal strain *Diaporthe kyushuensis* ZMU-48-1 was obtained from decayed leaves of *Acacia confusa* Merr. collected from Huanglübei Mountain, Jinwan District, Zhuhai, Guangdong Province, China, in October 2023. Briefly, the collected leaves were surface-sterilized with 2% (*v*/*v*) sodium hypochlorite for 2 min and 75% (*v*/*v*) ethanol for 1 min, followed by multiple washes with sterile water. The sterilized leaves were then cultured on Potato Dextrose Agar (PDA) medium at 30 °C. Pure cultures were obtained via the hyphal tip method, where actively growing hyphae at the colony edge were repeatedly transferred to fresh medium until a single, morphologically uniform isolate was secured [13]. The strain was deposited in the Microbial Resource Bank of the Zhuhai Campus, Zunyi Medical University (Zhuhai, China). Molecular identification was conducted via internal transcribed spacer (ITS) rDNA sequencing (Beijing Qingke Biotechnology Co., Ltd., Beijing, China) using the universal primers ITS1 (5′-TCCGTAGGTGAACCTGCGG-3′) and ITS4 (5′-TCCTCCGCTTATTGATATGC-3′). The ITS rDNA sequence of strain ZMU-48-1 has been deposited in the GenBank database under the accession number PV259067. Whole-genome sequencing of strain ZMU-48-1 was performed by Sangon Biotech Co., Ltd. (Shanghai, China).

Mycelial plugs were cultured in 50 mL PDB at 28 °C and 180 rpm for 6 days. Approximately 20 mg of dried mycelium was ground in liquid nitrogen and incubated with 400 μL Buffer Digestion at 65 °C for 1 h. Following RNase A treatment and protein precipitation with Buffer PF at −20 °C, the DNA was purified via centrifugation and isopropanol precipitation. PCR amplification was conducted using the following thermal profile: initial denaturation at 98 °C for 1 min; followed by 12 cycles of 98 °C for 10 s, 60 °C for 30 s, and 72 °C for 30 s; and a final extension at 72 °C for 5 min [12].

### 2.3. Fermentation

Seed cultures of *D. kyushuensis* ZMU-48-1 were obtained by transferring mycelial plugs from 2–3-day-old PDA plates into 250 mL Erlenmeyer flasks containing 100 mL PDB and incubating at 28 °C and 180 rpm for 3 days. For submerged production cultures, 10 mL aliquots of the seed culture were inoculated into 500 mL Erlenmeyer flasks containing 200 mL PDB supplemented with either 3% (*w*/*v*) NaCl or 3% (*w*/*v*) NaBr. The flasks were shaken at 28 °C and 180 rpm for 14 days, affording a total culture volume of 4.0 L for each condition. For rice solid-state fermentation, 100 g of rice and 100 mL of 3% (*w*/*v*) sea-salt solution were placed in 600 mL tissue culture bottles (20 kg of rice in total) and sterilized at 121 °C for 30 min. After cooling, each bottle was directly inoculated with several mycelial plugs from 2–3-day-old PDA cultures of *D. kyushuensis* ZMU-48-1 and incubated statically at 28 °C for 43 days. The resulting liquid and solid cultures were harvested separately for extraction and isolation.

### 2.4. Extraction and Isolation

The fermented rice medium was extracted three times with EtOH (50 L in total); the combined EtOH extracts were concentrated and partitioned with EtOAc (45 L) to afford the crude rice extract (66.7 g). The crude rice extract was fractionated by silica gel CC using a PE–EtOAc gradient (100:0 to 0:100, *v*/*v*) to give seven fractions (Fr. B1–B7). Fr. B4 and Fr. B5 were combined and further separated on silica gel with a CH_2_Cl_2_–MeOH gradient (20:1 to 2:1, *v*/*v*) to yield subfractions, among which Fr. D4 and Fr. D5 were prioritized for purification.

**Figure 2:**
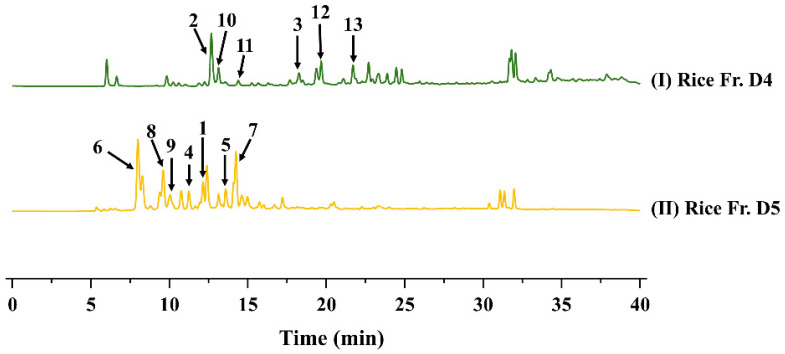
The HPLC analysis of different secondary metabolites in different medium.

Fr. D4 was purified by preparative HPLC using MeOH–H_2_O and CH_3_CN–H_2_O systems to afford compounds **10** (7 mg, 25% MeOH, *t*_R_ = 7.54 min), **2** (36 mg, 18% MeOH, *t*_R_ = 11.40 min), **11** (10 mg, 25% MeOH, *t*_R_ = 11.74 min), **13** (10 mg, 27% CH_3_CN, *t*_R_ = 17.47 min), **3** (12 mg, 30% CH_3_CN, *t*_R_ = 14.90 min), and **12** (9 mg, 40% CH_3_CN, *t*_R_ = 9.39 min). Fr. D5 was purified on the same system with CH_3_CN–H_2_O to yield compounds **1** (6 mg, 27% CH_3_CN, *t*_R_ = 8.77 min), **4** (17 mg, 28% CH_3_CN, *t*_R_ = 7.56 min), **5** (8 mg, 23% CH_3_CN, *t*_R_ = 9.82 min), **7** (15 mg, 17% CH_3_CN, *t*_R_ = 14.64 min) and **9** (12 mg, 22% CH_3_CN, *t*_R_ = 8.13 min); further elution with 20% CH_3_CN furnished compounds **6** (7 mg, *t*_R_ = 9.15 min) and **8** (5 mg, *t*_R_ = 12.33 min). The overall separation scheme and the chromatographic peaks corresponding to compounds **1**–**13** in the crude rice extract is summarized in Figure 2.

#### 2.4.1. Diaporthopyranone A (**1**)

Brownish yellow oil; [*α*]25 D −1.13 (c 0.1, MeOH); IR (KBr, ν_max_ cm^−1^): 3336, 2929, 2856, 2837, 1716, 1683, 1670, 1506, 1456, 1018, 669, 592, 547. For the ^1^H and ^13^C NMR spectroscopic data, see Table 1. HR-ESI-MS *m*/*z* 219.0992 [M + Na]^+^ (calcd for C_11_H_16_NaO_3_, 219.0997).

#### 2.4.2. Diaporthopyranone B (**2**)

Brown oil; IR (KBr, ν_max_ cm^−1^): 3383, 2929, 2856, 2505, 2077, 1714, 1647, 1456, 1338, 1188, 974, 659, 592. For the ^1^H and ^13^C NMR spectroscopic data, see Table 1. HR-ESI-MS *m*/*z* 195.0647 [M + H]^+^ (calcd for C_10_H_11_O_4_, 195.0657).

#### 2.4.3. Diaporthopyranone C (**3**)

Brown solid; IR (KBr, ν_max_ cm^−1^): 3361, 2929, 2856, 1732, 1716, 1635, 1506, 1456, 1338, 1085, 1029, 867, 827. For the ^1^H and ^13^C NMR spectroscopic data, see Table 1. HR-ESI-MS *m*/*z* 211.0961 [M + H]^+^ (calcd for C_11_H_15_O_4_, 211.0970).

#### 2.4.4. Diaporthopyranone D (**4**)

Brown solid; IR (KBr, ν_max_ cm^−1^): 3340, 2943, 2833, 1716, 1456, 1417, 1118, 1020, 667, 599. For the ^1^H and ^13^C NMR spectroscopic data, see Table 1. HR-ESI-MS *m*/*z* 241.1069 [M + H]^+^ (calcd for C_12_H_17_O_5_, 241.1076).

#### 2.4.5. Diaporthopyranone E (**5**)

Brownish yellow oil; [*α*]25 D −7.50 (c 0.1, MeOH); IR (KBr, ν_max_ cm^−1^): 3365, 2929, 2899, 2864, 2505, 2320, 2077, 1734, 1683, 1541, 1456, 1116, 1082, 972, 831, 667, 603. For the ^1^H and ^13^C NMR spectroscopic data, see Table 1. HR-ESI-MS *m*/*z* 227.0911 [M + H]^+^ (calcd for C_11_H_15_O_5_, 227.0919).

#### 2.4.6. Diaporthopyranone F (**6**)

Brownish yellow oil; [*α*]25 D +3.85 (c 0.168, MeOH); IR (KBr, ν_max_ cm^−1^): 3325, 2929, 2854, 2835, 1716, 1647, 1456, 1417, 1015, 659, 599, 555. For the ^1^H and ^13^C NMR spectroscopic data, see Table 1. HR-ESI-MS *m*/*z* 211.1326 [M + H]^+^ (calcd for C_12_H_19_O_3_, 211.1334).

#### 2.4.7. Diaporthopyranone G (**7**)

Amorphous powder; [*α*]25 D −3.08 (c 0.184, MeOH); IR (KBr, ν_max_ cm^−1^): 3373, 2935, 2866, 1770, 1714, 1699, 1635, 1558, 1456, 1417, 1338, 1118, 1029, 929, 887, 833, 667, 592, 547. For the ^1^H and ^13^C NMR spectroscopic data, see Table 1. HR-ESI-MS *m*/*z* 227.1276 [M + H]^+^ (calcd for C_12_H_19_O_4_, 227.1283).

#### 2.4.8. Diaporthopyranone H (**8**)

Amorphous powder; [*α*]25 D −10.57 (c 0.1, MeOH); IR (KBr, ν_max_ cm^−1^): 3363, 2941, 2833, 1714, 1635, 1456, 1417, 1116, 1022, 833, 667, 599, 547. For the ^1^H and ^13^C NMR spectroscopic data, see Table 1. HR-ESI-MS *m*/*z* 227.1277 [M + H]^+^ (calcd for C_12_H_19_O_4_, 227.1283).

### 2.5. ECD Calculations

Conformational searches were performed using the MMFF force field in Spartan’14 (Version 1.1.4). The resulting low-energy conformers within a 10 kcal mol^−1^ window were further optimized via DFT at the B3LYP/6-31G(d) level in Gaussian 09 (Revision B.01). Vibrational frequency calculations at the same level confirmed the absence of imaginary frequencies and provided Gibbs free energies (ΔG) at 298.15 K. ECD spectra were subsequently calculated for the stable conformers at the B3LYP/6-311G(d,p) level, employing the polarizable continuum model (PCM) for solvent effects. The Boltzmann-weighted ECD curves were generated using SpecDisth (Version 1.71), and Origin (Version 2018), and compared with experimental data. Computational resources were provided by the National Supercomputer Center in Guangzhou (Tianhe-2).

### 2.6. Antifungal Activity

Minimum inhibitory concentrations (MICs) were determined in liquid culture via a serial dilution method as described previously with minor modifications [14]. Briefly, six microtubes were prepared, each containing 0.5 mL of broth medium. Test compounds were dissolved in a 5% (*v*/*v*) aqueous DMSO solution. An initial screening was performed at 200 μg/mL for all compounds, and active ones were further evaluated using a targeted gradient (100, 80, 60, 40, and 20 μg/mL) to determine the precise MIC. A negative control containing 5% (*v*/*v*) DMSO was included in parallel and showed no inhibitory effect. Subsequently, 0.5 mL of a standardized spore suspension was added to each tube, followed by incubation at 28 °C for 24 h under identical conditions. Fungal growth was inspected visually, and the MIC was defined as the lowest concentration at which no visible growth was observed. Carbendazim was used as a positive control to validate the assay. All antifungal assays were performed in three independent biological replicates to ensure the reproducibility of the MIC values.

## 3. Results

### 3.1. HSQC-Based DeepSAT Analysis

**Figure 3:**
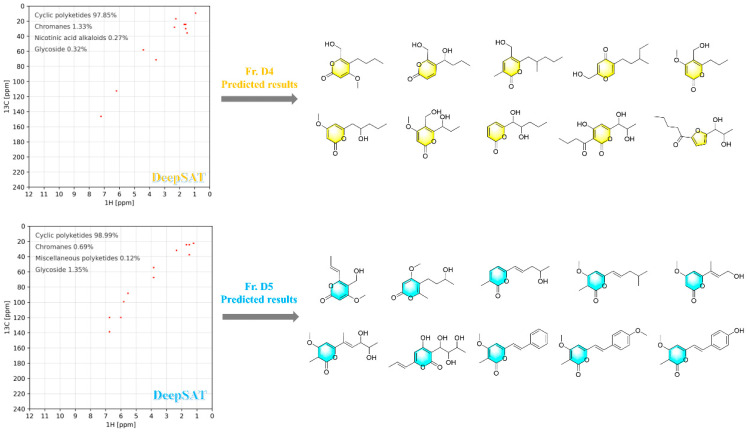
DeepSAT predicted the pyranone scaffold family from fraction Fr. D4 and Fr. D5.

After silica gel fractionation of the crude rice EtOAc extract (Fr. B1–B7), the combined Fr. B4 and Fr. B5 were further separated into subfractions Fr. D1–D7. To rapidly assess the dominant chemotypes, the ^1^H–^13^C HSQC spectra of all seven subfractions (Fr. D1–D7) were acquired and analyzed using the DeepSAT platform (https://www.deepsat.com/, accessed on 12 July 2025) [11]. DeepSAT independently suggested that only Fr. D4 and Fr. D5 were overwhelmingly enriched in cyclic polyketide features and returned top-ranked scaffold predictions centered on an α-pyranone (2*H*-pyran-2-one) type core, with a small set of closely related analogs differing mainly in side-chain substitution (Figure 3).

Guided by these HSQC–DeepSAT predictions, Fr. D4 and Fr. D5 were prioritized for targeted, repeated purification. This scaffold-focused workflow furnished a coherent series of structurally related α-pyranone derivatives, consistent with the predicted chemotype distribution. Collectively, the HSQC-based DeepSAT analysis provided a rapid, fraction-level rationale for prioritization and enabled efficient isolation of an α-pyranone analogue set from the rice culture extract.

### 3.2. Structural Characterization

**Figure 4:**
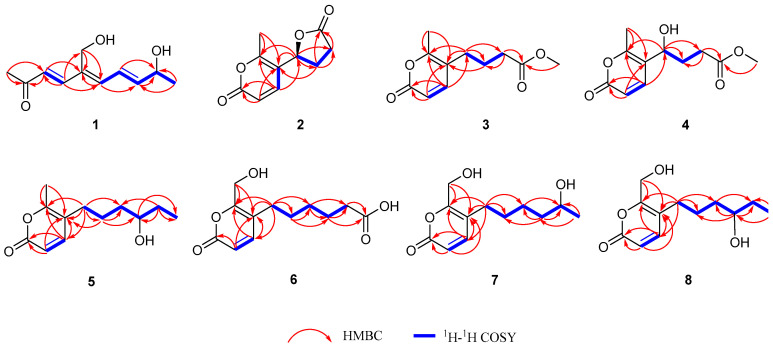
Key ^1^H-^1^H COSY and HMBC correlations of **1**–**8**.

**Figure 5:**
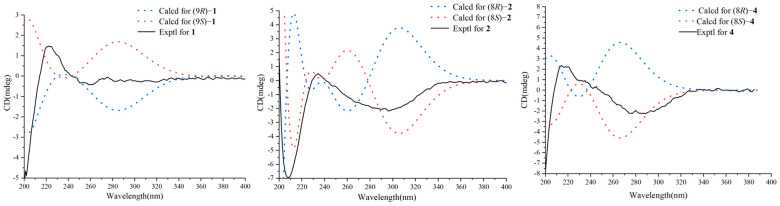
Experimental and predicted ECD spectra of compounds **1**, **2** and **4**.

Diaporthopyranone A (**1**) was obtained as a brownish yellow oil. The HR-ESI-MS spectrum of **1** revealed a pseudomolecular ion peak at *m*/*z* 219.0992 [M + Na]^+^, which is in accordance with the molecular formula C_11_H_16_NaO_3_ and indicates four degrees of unsaturation. Its IR spectrum exhibited absorptions for hydroxyl groups (3336 cm^−1^) and conjugated carbonyl groups (1670 cm^−1^). The ^1^H NMR spectrum revealed five olefinic protons at *δ*_H_ 6.41 (1H, d, *J* = 16.1 Hz, H-3), *δ*_H_ 7.27 (1H, d, *J* = 16.1 Hz, H-4), *δ*_H_ 6.64 (1H, d, *J* = 11.5 Hz, H-6), *δ*_H_ 6.78 (1H, dd, *J* = 15.0, 11.5 Hz, H-7), and *δ*_H_ 6.12 (1H, dd, *J* = 15.0, 5.8 Hz, H-8), one oxygenated methine *δ*_H_ 4.38 (1H, m, H-9), one oxygenated methylene *δ*_H_ 4.41 (2H, s, H-11), and two methyls *δ*_H_ 2.30 (3H, s, H-1) and *δ*_H_ 1.27 (3H, d, *J* = 6.5 Hz, H-10). The ^13^C and HSQC spectra revealed eleven signals, including two methyl signals at *δ*_C_ 23.3 (C-1) and *δ*_C_ 27.3 (C-10), one oxygenated methylene signal at *δ*_C_ 56.7 (C-11), five olefinic signals (*δ*_C_ 127.5, *δ*_C_ 147.9, *δ*_C_ 142.4, *δ*_C_ 125.2 and *δ*_C_ 146.4), one oxygenated methine signal at *δ*_C_ 68.7 (C-9), and two quaternary carbons (one olefinic at *δ*_C_ 137.4 and one carbonyl at *δ*_C_ 201.5) (Table 1). The presence of three double bonds and one carbonyl group results in a total of four degrees of unsaturation, indicating that compound **1** is a chain compound. A five-carbon chain consisting of one methyl, one oxygenated methine, and three olefinic methines could be identified by consecutive ^1^H-^1^H COSY correlations through H-10 to H-6, along with HMBC correlations from H-10 to C-8 and H-8 to C-6. Moreover, the HMBC correlations from H-6 to C-5 and C-4, and from H-3 to C-5 and C-1, and from H-1 to C-2 and C-3 established the long ten-carbon chain. Finally, the oxygenated methylene was connected to C-5 according to the HMBC correlations from H-11 to C-5, C-4, and C-6 (Figure 4). Thus, the planar structure of **1** was established. The *trans* configurations of Δ^3,4^ and Δ^7,8^ were confirmed by their larger coupling constants. And the *cis* configuration of Δ^5,6^ was established on the basis of NEOSY correlations between H-4 and H-6, H-7 and H-11. In addition, the configuration of C-9 was identified to be *R* according to the ECD calculations and comparisons of the experimental data (Figure 5). Therefore, the structure of **1** was established.

Diaporthopyranone B (**2**) was isolated as a brown oil, whose molecular formula was C_10_H_10_O_4_ with six degrees of unsaturation on the basis of the pseudomolecular ion peak at *m*/*z* 195.0647 [M + H]^+^ in the HR-ESI-MS spectrum. The IR spectrum exhibited absorption bands attributable to hydroxyl groups (3383 cm^−1^) and conjugated lactone carbonyl groups (1714 cm^−1^). ^1^H NMR revealed two neighboring coupling olefinic protons (*δ*_H_ 6.28, 7.61, d, *J* = 9.7 Hz, each 1H), an oxygenated methine (*δ*_H_ 5.56 dd, *J* = 9.7, 6.5 Hz, 1H), and a methyl (*δ*_H_ 2.35 s, 3H). Its ^13^C and DEPT spectra displayed ten carbon signals, including one methyl, two methylene, three methine (two olefinic, one oxygenated), and four quaternary carbon signals (two olefinic, two carbonyl) (Table 1). The four olefinic carbons, one carbonyl carbon, and one methyl form a methyl-substituted α-pyrone, which has been reported to be the main metabolite of *Diaporthe* fungi. The existence of a methyl-substituted α-pyrone could be further supported by the ^1^H-^1^H COSY correlation between H-3 and H-4 and the HMBC correlations from the methyl group to C-5 and C-6, from H-3 to C-2 and C-5, and from H-4 to C-5 and C-6 (Figure 3). In addition, an independent spin system consisting of one oxygenated methine and two methylenes could be identified in the ^1^H-^1^H COSY spectrum, along with a carbonyl carbon at *δ*_C_ 179.2 s (C-11), which formed a γ-butyrolactone. This butyrolactone ring was connected to C-5 in the pyrone ring on the basis of the HMBC correlations from H-4 to C-8 and from H-8 to C-4 and C-6 (Figure 4). Thus, the planar structure of **2** was established. The absolute configuration of 2 was assigned as 8*S* based on the good agreement between its experimental ECD spectrum and the calculated spectrum of the (8*S*)-2 isomer (Figure 5).

Diaporthopyranone C (**3**) was obtained as a brown solid. HR-ESI-MS revealed a pseudomolecular ion at *m*/*z* 211.0961 [M + H]^+^, which is consistent with the molecular formula C_11_H_14_O_4_ and indicates five degrees of unsaturation. Its IR spectrum showed characteristic absorptions for hydroxyl groups (3361 cm^−1^) and conjugated carbonyl groups (1716 cm^−1^). Its ^1^H and ^13^C NMR spectra also showed characteristic signals for a methyl-substituted α-pyrone (*δ*_H_ 6.17, 7.43, d, *J* = 9.4 Hz, each 1H, *δ*_H_ 2.26 s, 3H; *δ*_C_ 165.1 s, 113.8 d, 149.5 d, 116.7 s, 160.5 s, and 17.2 q) (Table 1) [15]. In addition, a spin system consisting of three methylenes was present in the ^1^H-^1^H COSY spectrum. The termini of this chain were linked with a formic ether according to the HMBC correlation from H-9 and H-10 to C-11, which formed a methyl butyrate. This side chain was connected to C-5 in the pyrone ring on the basis of the HMBC correlations from H-4 to C-8 and from H-8 to C-6 and C-4 (Figure 4). Therefore, the structure of **3** was established.

Diaporthopyranone D (**4**) was obtained as a brownish yellow oil, and its molecular formula was determined to be C_11_H_14_O_5_ via HR-ESI-MS analysis, which revealed a pseudomolecular ion peak at *m*/*z* 227.0911 [M + H]^+^. Its IR spectrum exhibited absorptions for hydroxyl groups (3365 cm^−1^) and conjugated carbonyl groups (1716 cm^−1^). The diagnostic signals, including a pair of coupled olefinic protons (*δ*_H_ 6.23, 7.63, d, *J* = 9.6 Hz, each 1H) and a methyl singlet (*δ*_H_ 2.29, s, 3H) in the ^1^H NMR spectrum, four olefinic (*δ*_C_ 114.1 d, 145.8 d, 120.3 s, and 160.1 s) and one carbonyl (*δ*_C_ 164.8 s) carbon, and one methyl (*δ*_C_ 17.0 q) in the ^13^C and DEPT spectra, revealed that **4** possesses a methyl-substituted α-pyrone nucleus (Table 1). In addition, the ^1^H-^1^H COSY spectrum revealed the existence of a spin system consisting of an oxygenated methine (*δ*_H_ 4.60, m, 1H) and two methylenes. The termini of this chain were linked with a formic ether according to the HMBC correlation from H-9 and H-10 to C-11, whereas the other end of this chain was connected to C-5 on the basis of the HMBC correlations from H-4 to C-8 and from H-8 to C-5 and C-6 (Figure 4). Thus, the planar structure of **4** was established. Comparison of the experimental ECD spectrum of **4** with the calculated ECD spectra indicated that it corresponds to the (8*S*)-**4** isomer, thereby establishing the absolute configuration at C-8 as *S* (Figure 5).

Diaporthopyranone E (**5**) was obtained as a brownish yellow oil. HR-ESI-MS revealed a pseudomolecular ion peak at *m*/*z* 211.1326 [M + H]^+^, which is consistent with the molecular formula C_12_H_18_O_3_. Its IR spectrum showed absorptions for a hydroxyl group (3325 cm^−1^), conjugated carbonyl groups (1647 cm^−1^), and a C–O stretching vibration at 1016 cm^−1^. The ^1^H, ^13^C and DEPT spectra also revealed diagnostic signals for a methyl-substituted α-pyrone nucleus (Table 1) [16]. In addition, a six-carbon chain consisting of four methylenes, one methyl, and one oxygenated methine could be identified by consecutive ^1^H-^1^H COSY correlations through H-8 to H-13, along with HMBC correlations from H-8 to C-10, from the oxygenated methine (*δ*_H_ 3.54, dt, *J* = 7.7, 3.7 Hz, 1H) to C-13 and C-8. This chain was linked to C-5 in the α-pyrone ring on the basis of the HMBC correlations from H-4 to C-8 and from H-8 to C-4, C-5 and C-6 (Figure 4). Thus, the planar structure of **5** was established. However, the limited availability of the isolate precluded the application of Mosher’s method, leaving its absolute configuration unassigned at this stage.

Diaporthopyranone F (**6**) was isolated as a brown solid. Its molecular formula was established as C_12_H_16_O_5_ with five degrees of unsaturation according to the pseudomolecular ion peak at *m*/*z* 241.1069 [M + H]^+^ in the HR-ESI-MS spectrum. The IR spectrum showed absorptions for a hydroxyl group (3340 cm^−1^), conjugated carbonyl groups (1683 cm^−1^), and a C–O stretching vibration at 1020 cm^−1^. The ^1^H NMR spectrum revealed two coupled olefinic protons (*δ*_H_ 6.27, 7.48, d, *J* = 9.4 Hz, each 1H) and an oxygenated methylene (*δ*_H_ 4.39, s, 2H). The ^13^C and DEPT spectra revealed four olefinic carbons (*δ*_C_ 116.0 d, 149.5 d, 118.8 s, and 160.1 s), a carbonyl carbon (*δ*_C_ 164.6 s), and an oxygenated methylene (*δ*_C_ 59.2 t) (Table 1). These data suggested that **6** possesses a hydroxymethyl substituent in the α-pyrone ring [17]. In addition, n-hexanoic acid was identified on the basis of consecutive ^1^H-^1^H COSY correlations through H-8 to H-12, along with HMBC correlations from H-8 to C-10, from H-9 to C-11, and from H-11 and H-12 to C-13 [18]. This side chain was connected to C-5 according to the HMBC correlations from H-4 to C-8 and from H-8 to C-6, C-5 and C-4 (Figure 4). Thus, the structure of 6 was established.

Diaporthopyranone G (**7**) was obtained as an amorphous powder. HR-ESI-MS revealed a pseudomolecular ion peak at *m*/*z* 227.1276 [M + H]^+^, which is consistent with the molecular formula C_12_H_18_O_4_. Its IR spectrum showed absorption bands for a hydroxyl group (3373 cm^−1^) and conjugated carbonyl groups (1699 cm^−1^). Its NMR spectra showed characteristic signals for a hydroxymethyl-substituted α-pyrone ring (*δ*_H_ 6.28, 7.48, d, *J* = 9.4 Hz, each 1H, *δ*_H_ 4.39 s, 2H; *δ*_C_ 164.6 s, 116.0 d, 149.5 d, 118.8 s, 160.1 s, and 59.2 t) (Table 1). Similarly, a six-carbon chain consisting of four methylenes, one methyl, and one oxygenated methine was identified by consecutive ^1^H-^1^H COSY correlations through H-8 to H-13, along with HMBC correlations from H-8 to C-10, from H-9 to C-11, and from H-13 to the oxygenated methine and C-11. Finally, the location of this side chain was determined by the HMBC correlations from H-4 to C-8 and from H-8 to C-6, C-5 and C-4 (Figure 4). With the planar scaffold of **7** firmly established, its absolute stereochemistry remained unassigned, as the scarcity of the isolate precluded the requisite chemical derivatization.

Diaporthopyranone H (**8**) was obtained as an amorphous powder. HR-ESI-MS revealed a pseudomolecular ion peak at *m*/*z* 227.1277 [M + H]^+^, which is consistent with the molecular formula C_12_H_18_O_4_ and indicates four degrees of unsaturation. Its IR spectrum exhibited absorption bands for a hydroxyl group (3363 cm^−1^), conjugated carbonyl groups (1635 cm^−1^), and a C–O stretching vibration at 1022 cm^−1^. The ^1^H, ^13^C, and DEPT NMR spectra revealed signals for a hydroxymethyl-substituted α-pyrone ring (*δ*_H_ 6.27, 7.48, d, *J* = 9.4 Hz, each 1H; *δ*_H_ 4.39 s, 2H; *δ*_C_ 164.6 s, 116.0 d, 149.5 d, 118.8 s, 160.1 s and 59.2 t) (Table 1). Similarly, a six-carbon chain was identified by consecutive ^1^H-^1^H COSY correlations through H-8 to H-13, along with HMBC correlations from H-8 to C-10, from H-9 to C-11, and from the oxygenated methine H-11 (*δ*_H_ 3.44, m, 1H) to C-9, C-10, and C-13. This side chain was located at C-5 on the basis of the HMBC correlations from H-4 to C-8 and from H-8 to C-6, C-5 and C-4 (Figure 4). The planar structures of compounds **5**, **7**, and **8** were thus defined. However, their absolute configurations remain unassigned at this stage. Because their stereocenters are located on highly flexible, saturated aliphatic chains distant from the primary α-pyrone chromophore, ECD analysis would yield highly conformationally averaged and unreliable data. Furthermore, the limited supply of these minor isolates precluded the necessary chemical derivatization (e.g., Mosher’s method) required for assigning such flexible distal alcohols.

**Table 1:** ^1^H (600 MHz) and ^13^C (150 MHz) NMR spectroscopic data of compounds **1**–**8.**

Nos.	1 ^b^		2 ^b^		3 ^b^		4 ^b^		5 ^b^		6 ^a^		7 ^b^		8 ^b^	
	*δ*_H_ Type (*J*)	*δ*_C_ Type	*δ*_H_ Type (*J*)	*δ*_C_ Type	*δ*_H_ Type (*J*)	*δ*_C_ Type	*δ*_H_ Type (*J*)	*δ*_C_ Type	*δ*_H_ Type (*J*)	*δ*_C_ Type	*δ*_H_ Type (*J*)	*δ*_C_ Type	*δ*_H_ Type (*J*)	*δ*_C_ Type	*δ*_H_ Type (*J*)	*δ*_C_ Type
1	2.30 s	23.3 q	/	/	/	/	/	/	/	/	/	/	/	/	/	/
2		201.5 s		164.0 s		165.1 s		164.8 s		163.0 s		164.6 s		164.6 s		164.6 s
3	6.41 d (16.1)	127.5 d	6.28 d (9.7)	114.6 d	6.17 d (9.4)	113.8 d	6.23 d (9.6)	114.1 d	6.14 d (9.4)	113.5 d	6.27 d (9.4)	116.0 d	6.28 d (9.4)	116.0 d	6.27 d (9.4)	116.0 d
4	7.27 d (16.1)	147.9 d	7.61 d (9.7)	144.5 d	7.43 d (9.4)	149.5 d	7.63 d (9.6)	145.8 d	7.17 d (9.4)	147.1 d	7.48 d (9.4)	149.5 d	7.48 d (9.4)	149.5 d	7.48 d (9.4)	149.5 d
5	/	137.4 s		115.7 s		116.7 s		120.3 s		115.3 s		118.8 s		118.8 s		118.8 s
6	6.64 d (11.5)	142.4 d	/	162.3 s	/	160.5 s	/	160.1 s	/	158.6 s	/	160.1 s	/	160.1 s	/	160.1 s
7	6.78 dd (15.0, 11.5)	125.2 d	2.35 s	17.2 q	2.26 s	17.2 q	2.29 s	17.0 q	2.23 s	17.4 q	4.39 s	59.2 t	4.39 s	59.2 t	4.39 s	59.2 t
8	6.12 dd (15.0, 5.8)	146.4 d	5.56 dd (9.7, 6.5)	78.0 d	2.40 m	29.4 t	4.60 m	67.7 d	2.31 q (7.4)	29.6 t	2.45 m	29.5 t	2.45 t (7.7)	29.6 t	2.45 m	29.6 t
9	4.38 m	68.7 d	2.52 m	29.7, t	1.80 m	25.9 t	1.95 m	33.0 t	1.51 m	26.0 t	1.55 m	31.1 t	1.54 m	31.4 t	1.65 m	27.6 t
10	1.27 d (6.5)	27.3 q	2.73 m	30.0, t	2.37 m	33.7 t	2.42 t (7.1)	30.8 t	/	36.2 t	1.40 m	29.8 t	1.45 m	26.3 t	1.41 m	37.1 t
11	4.41 s	56.7 t	/	179.2 s		175.3 s		175.4 s	3.54 dt (7.7, 3.7)	73.1 d	1.63 m	26.5 t	1.31 m	39.9 t	3.44 m	73.6 t
12									1.41 m	30.5 t	2.24 m	36.7 d	3.71 dd (6.4, 5.0)	68.4 d	/	31.1 d
13									0.94 t (7.5)	10.0 q	/	180.0 s	1.15 d (6.2)	23.5 q	/	10.4 q
OCH_3_-11						52.1 q		52.1 q								

Note: ^a^ Measured in CDCl_3_; ^b^ Measured in CD_3_OD.

The planar structures of compounds **5**, **7**, and **8** were thus defined. However, their absolute configurations remain unassigned. Extensive computational efforts, including TDDFT-ECD, DP4+ NMR analysis, and optical rotation calculations, proved inadequate for reliable assignment due to severe conformational averaging arising from their highly flexible, saturated aliphatic side chains. Definitive determination via chemical derivatization, such as the Mosher’s method, was also prohibited by the limited supply of these minor isolates.

Although the isolated compounds share a well-known α-pyrone core scaffold characteristic of the genus *Diaporthe*, compounds **1**–**8** represent new chemical entities that significantly expand the structural diversity of this class. The precise structural novelties of these analogues lie primarily in their diverse side-chain functionalizations relative to previously reported metabolites. For instance, compound **2** is distinguished by an advanced fused γ-butyrolactone ring, whereas compound **4** features a specific C-9 hydroxylation that differentiates it from its known deoxy analogue **3**. Furthermore, compounds **6**–**8** exhibit distinct alkyl chain elongations coupled with unique terminal or sub-terminal oxidation states. These atom-level modifications collectively enrich the known chemodiversity of *Diaporthe*-derived pyranones.

On the basis of spectroscopic analyses and comparisons with reported data, the remaining metabolites were identified as five known pyranone derivatives, namely, diaporpyrone D (**9**) [8], diaporpyrone F (**10**) [8], diaporpyrone A (**11**) [9] and 5-[(1*S*)-1-hydroxyhexyl]-6-methyl-2*H*-pyran-2-one (**12**) [10], phomopyrone D (**13**) [19].

### 3.3. Putative Biosynthetic Pathways

**Figure 6:**
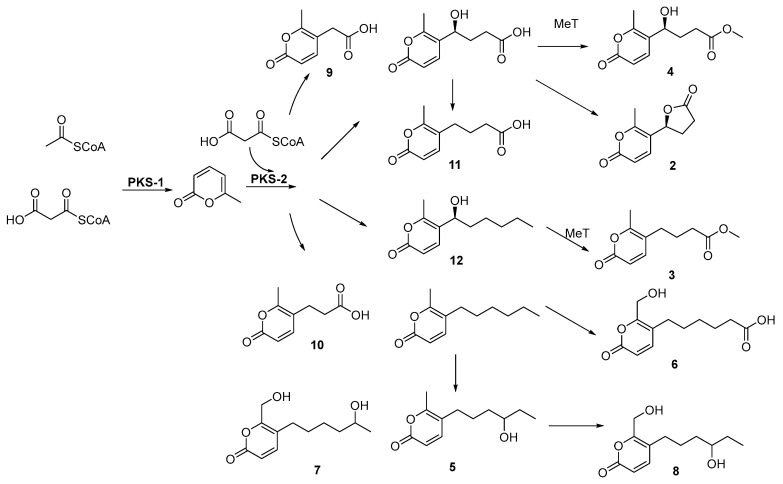
Proposed biosynthetic pathway of compounds **2**–**12.**

The putative biosynthetic assembly of these pyrone polyketides is hypothesized to begin with the iterative condensation of acetyl-CoA and malonyl-CoA units, initiated by a putative non-reducing polyketide synthase PKS-1 to generate the foundational 6-methyl-α-pyrone core. This scaffold is proposed to serve as the priming substrate for a secondary polyketide synthase, PKS-2, which likely orchestrates further chain elongation and programmed ketoreduction to yield diversified intermediates such as the carboxylic acids **9** and **10** and the reduced analogs **11** and **12**. The structural complexity of the library is subsequently elaborated through a proposed suite of post-PKS tailoring enzymes: an O-methyltransferase (MeT) may catalyze the esterification of terminal carboxylic acids to produce methyl esters **3** and **4**, while the formation of the bicyclic lactone **2** likely proceeds via the enzymatic or spontaneous cyclization of a hydroxylated side-chain precursor. Furthermore, late-stage oxidative processing, presumably mediated by cytochrome P450 monooxygenases, is hypothesized to introduce significant functional diversity through ω- or (ω−1)-oxidation of the aliphatic side chains and the hydroxylation of the C6-methyl group to a hydroxymethyl moiety [20]. These modifications would lead to the production of terminal dicarboxylates like **6** and polyhydroxylated derivatives such as **7** and **8**, illustrating a highly branched and modular biosynthetic logic characteristic of fungal specialized metabolism (Figure 6).

### 3.4. Antifungal Activities

**Figure 7:**
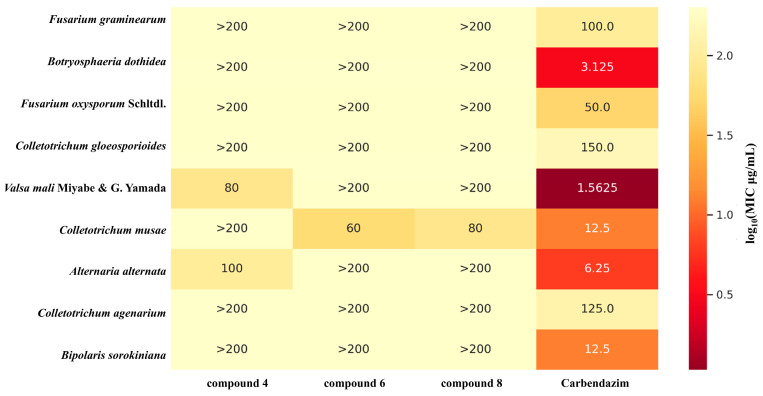
Antifungal activities (MICs, μg/mL) of compounds **4**, **6** and **8** against plant pathogenic fungi.

All the isolated compounds (**1**–**13**) were evaluated for their antifungal activity against nine plant pathogenic fungi, namely, *Fusarium graminearum*, *Botryosphaeria dothidea*, *Fusarium oxysporum* Schltdl., *Colletotrichum gloeosporioides*, *Valsa mali*, *Colletotrichum musae*, *Alternaria alternata*, *Colletotrichum agenarium*, and *Bipolaris sorokiniana*. As shown in Figure 7, compound **4** showed moderate inhibitory activity against *Alternaria alternata* and *Valsa mali*, with MIC values of 100 and 80 μg/mL, respectively. In comparison, the positive control carbendazim had an MIC of 6.25 μg/mL against *C. musae* and 1.0625 μg/mL against *C. gloeosporioides*. In addition, compounds **6** and **8** inhibited *Colletotrichum musae*, with MIC values of 60 and 80 μg/mL, respectively, whereas carbendazim exhibited an MIC of 12.5 μg/mL against this pathogen. The remaining compounds displayed only weak or negligible activity under the same conditions. Overall, these data indicate that the pyranone derivatives, particularly compound **4** against *V. mali* and *A. alternata*, and compounds **6** and **8** against *C. musae*, possess measurable antifungal potential (representative images of the growth inhibition are provided in Supplementary Figure S1), although their potency is still inferior to that of the commercial fungicide carbendazim.

## 4. Discussion

The identification of bioactive secondary metabolites from endophytic fungi is frequently impeded by the rediscovery of known compounds and the masking of minor constituents by major ones. To overcome these bottlenecks, advanced NMR-based dereplication tools have been developed. For instance, computational tools like MixONat have proven highly effective in dereplicating complex mixtures by matching experimental ^13^C and DEPT NMR signals against predefined structural databases [21]. However, their inherent reliance on existing databases can potentially limit the identification of truly novel scaffolds. In contrast, the HSQC-based DeepSAT strategy leverages convolutional neural networks (CNNs) to directly predict chemical fingerprints and scaffold classes from 2D HSQC spectra, circumventing the strict requirement for exact database matches [11]. Recently, this cutting-edge artificial intelligence platform has been successfully employed by researchers to rapidly annotate structural families within unpurified marine cyanobacterial extracts [22] and to guide the discovery of novel polyketides from mangrove-derived fungi [23], demonstrating its robust capability in early-stage chemotype prioritization. Building upon these emerging applications, in this study, we effectively adapted the DeepSAT workflow to prioritize the isolation of an α-pyrone chemotype from the terrestrial endophytic fungus *Diaporthe kyushuensis* ZMU-48-1. This targeted approach successfully facilitated the recovery of thirteen pyranone analogs, including eight novel compounds (**1**–**8**), thereby validating the utility of deep learning-assisted profiling in navigating endophytic metabolomes to access specific chemical spaces.

Interpreted within the context of antifungal discovery, the biological evaluation indicated that while the overall potency of the isolated pyranones was moderate compared to the commercial fungicide carbendazim, specific analogues exhibited distinct species-selectivity. A preliminary analysis of the structure-activity relationships (SAR) suggests that oxidative modifications on the pyrone scaffold may influence this selectivity. Notably, compounds **6** and **8**, which feature a C-6 hydroxymethyl group, showed selective inhibition against *C. musae*, whereas analogues with a C-6 methyl group (such as **1**–**5**) were inactive against this specific pathogen. Furthermore, compound **4** differs from the inactive compound **3** primarily by the presence of a hydroxyl group at the C-9 position, which appears to be crucial for its activity against *V. mali* and *A. alternata*. While the sparse data and weak-to-moderate MIC values (60–100 μg/mL) limit broad mechanistic interpretations, such oxidative functionalization is a well-documented strategy in fungal secondary metabolism to alter target specificity [24]. From a broader ecological perspective, the production of these specific metabolites, albeit with low in vitro potency, parallels the chemical defense mechanisms of other bioactive endophytic fungi (e.g., *Trichoderma* and *Pestalotiopsis* spp.), which deploy diverse functionalized polyketides against competing phytopathogens [25,26]. Ultimately, rather than representing immediate agricultural leads, these specific α-pyrone scaffolds serve as a preliminary proof-of-concept for our targeted discovery methodology. They highlight the necessity of future structural optimization (e.g., at C-6 and C-9) to enhance antifungal efficacy.

## 5. Conclusions

In summary, the implementation of an HSQC-based DeepSAT workflow successfully streamlined the metabolic profiling and prioritization of the endophytic fungus *D. kyushuensis* ZMU-48-1. By rapidly identifying an α-pyrone-dominated chemotype within complex fractions, this structure-guided approach facilitated the targeted isolation of a focused library of thirteen pyranone congeners, including eight novel metabolites, diaporthopyranone A–H (**1**–**8**). Biological evaluation revealed that these analogues exhibit only preliminary, low-potency antifungal activity (MICs 60–100 μg/mL) compared to the highly potent commercial fungicide carbendazim. Therefore, rather than establishing these compounds as promising antifungal leads, these findings more broadly validate the efficacy of HSQC-DeepSAT analysis as an efficient, structure-informed strategy to rapidly prioritize specific chemical spaces and accelerate the targeted discovery of natural product classes from complex fungal metabolomes.

## 6. Patents

The work reported in this manuscript has resulted in two patent applications filed with the China National Intellectual Property Administration (CNIPA). These include “A pyranone natural product with antifungal activity, its preparation method, and application” (Application No. 202511541679.X) and “A preparation method of pyranone natural products and their use in agricultural antifungal agents” (Application No. 202511541673.2).

## Data Availability

The datasets used and/or analyzed during the current study are available from the corresponding author on reasonable request.

## Supplementary Materials

The following supporting information can be downloaded at: https://www.mdpi.com/article/10.3390/jof12030161/s1, Figure S1: Visual assessment of the antifungal activities of compounds **4**, **6**, and **8** against *Botryosphaeria dothidea* (A–C), *Colletotrichum gloeosporioides* (D–E), and *Colletotrichum musae* (F–G); Figure S2: HRESIMS spectrum of compound **1**; Figure S3: IR spectrum of compound **1**; Figure S4: ECD spectra of compound **1** in MeOH; Figure S5: ^1^H-NMR (600 MHz, CD_3_OD) spectrum of compound **1**; Figure S6: ^13^C-NMR (150 MHz, CD_3_OD) spectrum of compound **1**; Figure S7: HSQC spectrum of compound **1**; Figure S8: HMBC spectrum of compound **1**; Figure S9: ^1^H-^1^H COSY spectrum of compound **1**; Figure S10: NOESY spectrum of compound **1**; Figure S11: HRESIMS spectrum of compound **2**; Figure S12: IR spectrum of compound **2**; Figure S13: ECD spectra of compound **2** in MeOH; Figure S14: ^1^H-NMR (600 MHz, CD_3_OD) spectrum of compound **2**; Figure S15: ^13^C-NMR and DEPT (150 MHz, CD_3_OD) spectrum of compound **2**; Figure S16: HSQC spectrum of compound **2**; Figure S17: HMBC spectrum of compound **2**; Figure S18: ^1^H-^1^H COSY spectrum of compound **2**; Figure S19: HRESIMS spectrum of compound **3**; Figure S20: IR spectrum of compound **3**; Figure S21: ECD spectra of compound **4** in MeOH; Figure S22: ^1^H-NMR (600 MHz, CD_3_OD) spectrum of compound **3**; Figure S23: ^13^C-NMR and DEPT (150 MHz, CD_3_OD) spectrum of compound **3**; Figure S24: HSQC spectrum of compound **3**; Figure S25: HMBC spectrum of compound **3**; Figure S26: ^1^H-^1^H COSY spectrum of compound **3**; Figure S27: HRESIMS spectrum of compound **4**; Figure S28: IR spectrum of compound **4**; Figure S29: ^1^H-NMR (600 MHz, CD_3_OD) spectrum of compound **4**; Figure S30: ^13^C-NMR and DEPT (150 MHz, CD_3_OD) spectrum of compound **4**; Figure S31: HSQC spectrum of compound **4**; Figure S32: HMBC spectrum of compound **4**; Figure S33: ^1^H-^1^H COSY spectrum of compound **4**; Figure S34: HRESIMS spectrum of compound **5**; Figure S35: IR spectrum of compound **5**; Figure S36: ^1^H-NMR (600 MHz, CDCl_3_) spectrum of compound **5**; Figure S37: ^13^C-NMR and DEPT (150 MHz, CDCl_3_) spectrum of compound **5**; Figure S38: HSQC spectrum of compound **5**; Figure S39: HMBC spectrum of compound **5**; Figure S40: ^1^H-^1^H COSY spectrum of compound **5**; Figure S41: HRESIMS spectrum of compound **6**; Figure S42: IR spectrum of compound **6**; Figure S43: ^1^H-NMR (600 MHz, CD_3_OD) spectrum of compound **6**; Figure S44: ^13^C-NMR and DEPT (150 MHz, CD_3_OD) spectrum of compound **6**; Figure S45: HSQC spectrum of compound **6**; Figure S46: HMBC spectrum of compound **6**; Figure S47: ^1^H-^1^H COSY spectrum of compound **6**; Figure S48: HRESIMS spectrum of compound **7**; Figure S49: IR spectrum of compound **7**; Figure S50: ^1^H-NMR (600 MHz, CD_3_OD) spectrum of compound **7**; Figure S51: ^13^C-NMR and DEPT (150 MHz, CD_3_OD) spectrum of compound **7**; Figure S52: HSQC spectrum of compound **7**; Figure S53: HMBC spectrum of compound **7**; Figure S54: 1H-1H COSY spectrum of compound **7**; Figure S55: HRESIMS spectrum of compound **8**; Figure S56: IR spectrum of compound **8**; Figure S57: ^1^H-NMR (600 MHz, CD_3_OD) spectrum of compound **8**; Figure S58: ^13^C-NMR and DEPT (150 MHz, CD_3_OD) spectrum of compound **8**; Figure S59: HSQC spectrum of compound **8**; Figure S60: HMBC spectrum of compound **8**; Figure S61: ^1^H-^1^H COSY spectrum of compound **8**.
