# Supplementary material for: Rapid Discovery of Antifungal α-Pyrone Analogs from Diaporthe kyushuensis ZMU-48-1 via an HSQC-Based DeepSAT Strategy
Source: J Fungi (Basel). 2026 Feb 25;12(3):161. doi: 10.3390/jof12030161 (PMC13027769; doi:10.3390/jof12030161)
Supplement: Supplementary file 1 [file jof-12-00161-s001.zip › jof-4163625-supplementary.pdf]

Supplementary Material

Rapid Discovery of Antifungal α-Pyrone Analogs from *Diaporthe kyushuensis* ZMU-48-1 via an HSQC-Based DeepSAT Strategy

Siwen Yuan[†,*], Jiaqi Zheng[†], Xijing Wang, Min Yan, Haiwen Wang, Tianpeng Yin*

School of Bioengineering, Key Laboratory of Quality Control and Evaluation of Traditional Chinese and Ethnic Medicines, Administration for Market Regulation of Guizhou Province, Zunyi Medical University, Zhuhai, 519041

*** Corresponding authors: E-mail: ytp@zmu.edu.cn (T. Yin), yuansiwen@zmuzh.edu.cn (S. Yuan)**

† These authors contributed equally to this work.

List of Supporting Information
content

Figure S1: Visual assessment of the antifungal activities of compounds 4, 6, and 8 against *Botryosphaeria dothidea* (A-C), *Colletotrichum gloeosporioides* (D-E), and *Colletotrichum musae* (F-G). Carbendazim and 5% DMSO were used as positive and negative controls, respectively. 5

Figure S2: HRESIMS spectrum of compound 1 5

Figure S3: IR spectrum of compound 1........6

Figure S4: ECD spectra of compound 1 in MeOH........6

Figure S5: ^{1}H-NMR (600 MHz, CD_3OD) spectrum of compound 1 7

Figure S6: ^{13}C-NMR (150 MHz, CD_3OD) spectrum of compound 1 7

Figure S7: HSQC spectrum of compound 1........8

Figure S8: HMBC spectrum of compound 1 8

Figure S9: ^{1}H-^{1}H COSY spectrum of compound 1........9

Figure S10: NOESY spectrum of compound 1 9

Figure S11: HRESIMS spectrum of compound 2........10

Figure S12: IR spectrum of compound 2 10

Figure S13: ECD spectra of compound 2 in MeOH........11

Figure S14: ^{1}H-NMR (600 MHz, CD_3OD) spectrum of compound 2 11

Figure S15: ^{13}C-NMR and DEPT (150 MHz, CD_3OD) spectrum of compound 2........12

Figure S16: HSQC spectrum of compound 2........12

Figure S17: HMBC spectrum of compound 2 13

Figure S18: ^{1}H-^{1}H COSY spectrum of compound 2........13

Figure S19: HRESIMS spectrum of compound 3........14

Figure S20: IR spectrum of compound 3 14

Figure S21: ECD spectra of compound 4 in MeOH........15

Figure S22: ^{1}H-NMR (600 MHz, CD_3OD) spectrum of compound 3 15

Figure S23: ^{13}C-NMR and DEPT (150 MHz, CD_3OD) spectrum of compound 3 16

Figure S24: HSQC spectrum of compound 3 16

Figure S25: HMBC spectrum of compound 3 17

Figure S26: ^{1}H-^{1}H COSY spectrum of compound 3 17

Figure S27: HRESIMS spectrum of compound 4 18

Figure S28: IR spectrum of compound 4 18

Figure S29: ^{1}H-NMR (600 MHz, CD_3OD) spectrum of compound 4 19

Figure S30: ^{13}C-NMR and DEPT (150 MHz, CD_3OD) spectrum of compound 4 19

Figure S31: HSQC spectrum of compound 4 20

Figure S32: HMBC spectrum of compound 4 20

Figure S33: ^{1}H-^{1}H COSY spectrum of compound 4 21

Figure S34: HRESIMS spectrum of compound 5 21

Figure S35: IR spectrum of compound 5 22

Figure S36: ^{1}H-NMR (600 MHz, $CDCl_3$) spectrum of compound 5 22

Figure S37: ^{13}C-NMR and DEPT (150 MHz, $CDCl_3$) spectrum of compound 5 23

Figure S38: HSQC spectrum of compound 5 23

Figure S39: HMBC spectrum of compound 5 24

Figure S40: ^{1}H-^{1}H COSY spectrum of compound 5 24

Figure S41: HRESIMS spectrum of compound 6 25

Figure S42: IR spectrum of compound 6 25

Figure S43: ^{1}H-NMR (600 MHz, CD_3OD) spectrum of compound 6 26

Figure S44: ^{13}C-NMR and DEPT (150 MHz, CD_3OD) spectrum of compound 6 26

Figure S45: HSQC spectrum of compound 6 27

Figure S46: HMBC spectrum of compound 6 27

Figure S47: ^{1}H-^{1}H COSY spectrum of compound 6 28

Figure S48: HRESIMS spectrum of compound 7 28

Figure S49: IR spectrum of compound 7 29

Figure S50: ^{1}H-NMR (600 MHz, CD_3OD) spectrum of compound 7 29

Figure S51: ^{13}C-NMR and DEPT (150 MHz, CD_3OD) spectrum of compound 7 30

Figure S52: HSQC spectrum of compound 7..30

Figure S53: HMBC spectrum of compound 7 31

Figure S55: HRESIMS spectrum of compound 8..32

Figure S56: IR spectrum of compound 8 32

Figure S57: ^{1}H-NMR (600 MHz, CD_3OD) spectrum of compound 8 33

Figure S58: ^{13}C-NMR and DEPT (150 MHz, CD_3OD) spectrum of compound 8 33

Figure S59: HSQC spectrum of compound 8..34

Figure S60: HMBC spectrum of compound 8 34

Figure S61: ^{1}H-^{1}H COSY spectrum of compound 8...35

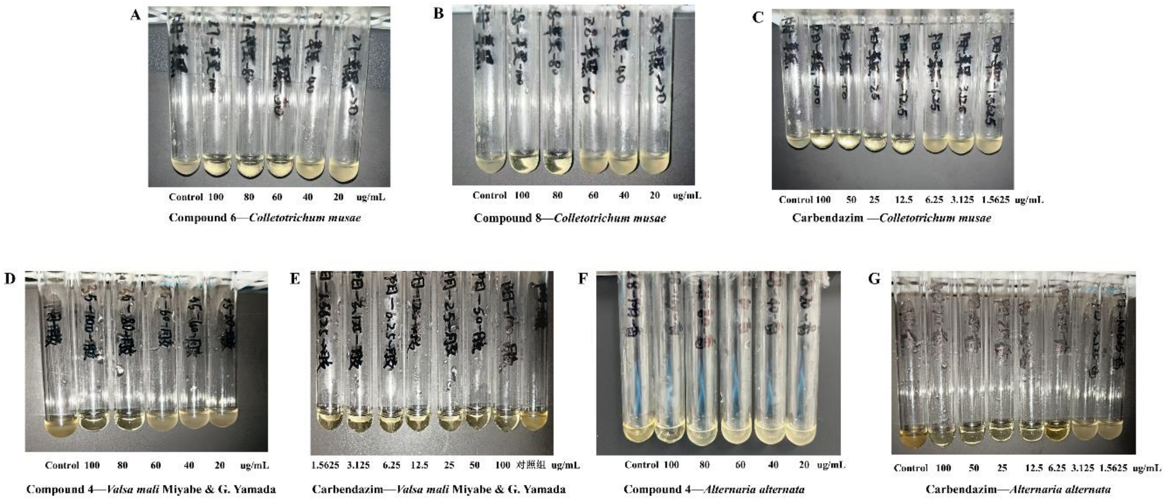

Figure S1: Visual assessment of the antifungal activities of compounds 4, 6, and 8 against *Botryosphaeria dothidea* (A-C), *Colletotrichum gloeosporioides* (D-E), and *Colletotrichum musae* (F-G). Carbendazim and 5% DMSO were used as positive and negative controls, respectively.

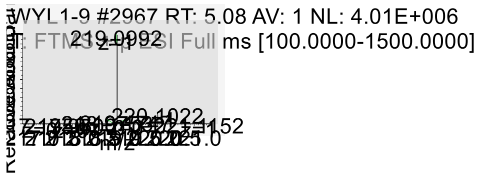

Figure S2: HRESIMS spectrum of compound 1

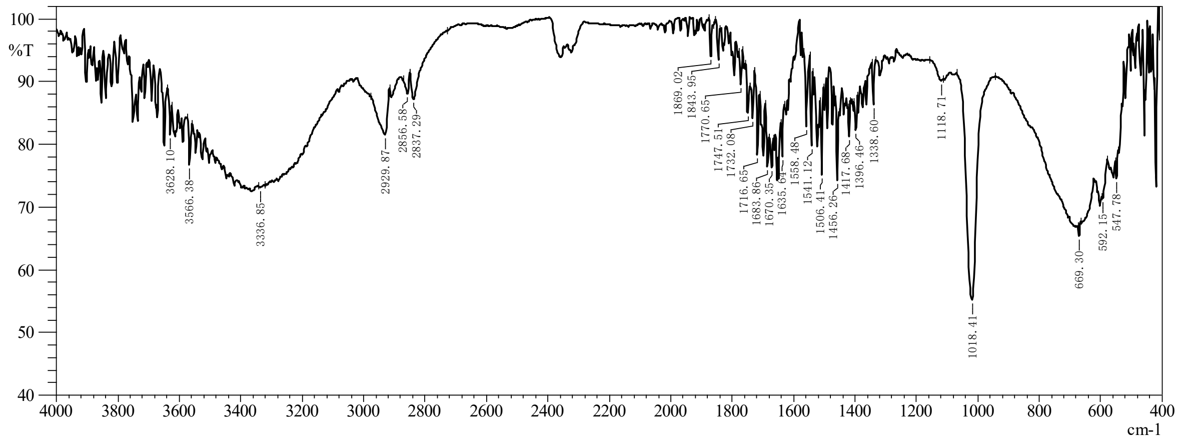

Figure S3: IR spectrum of compound 1

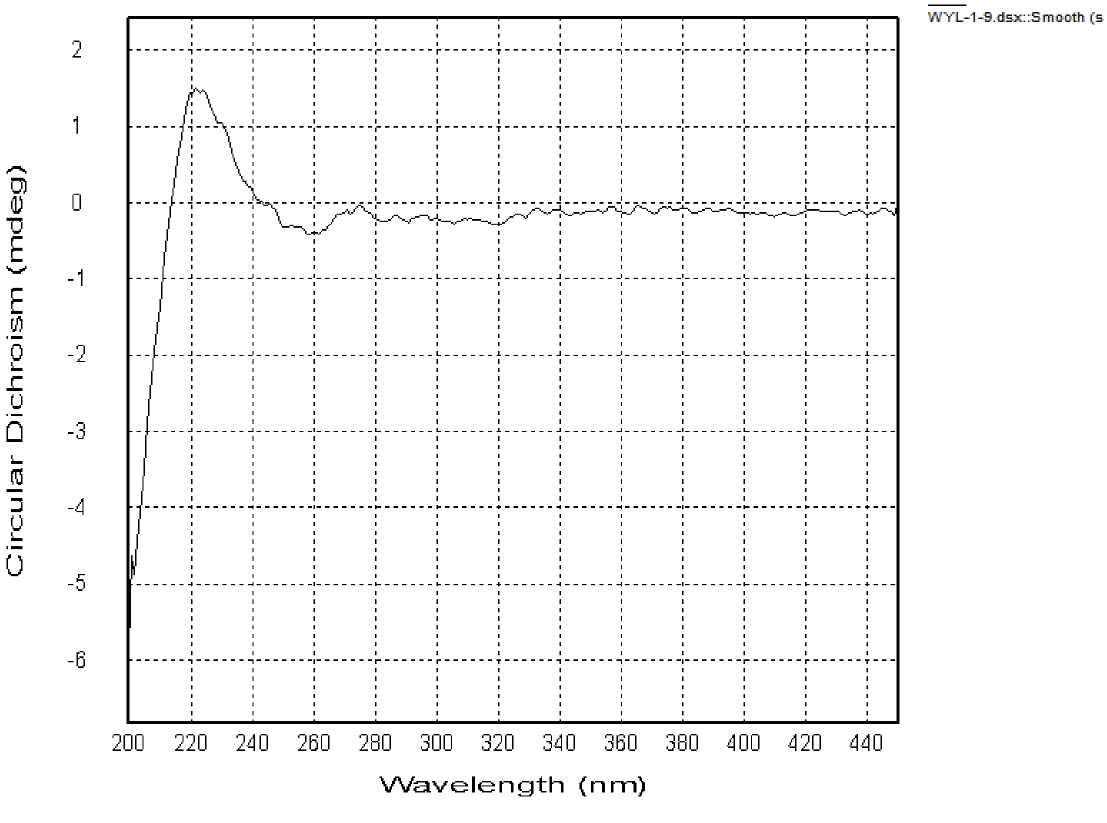

Figure S4: ECD spectra of compound 1 in MeOH

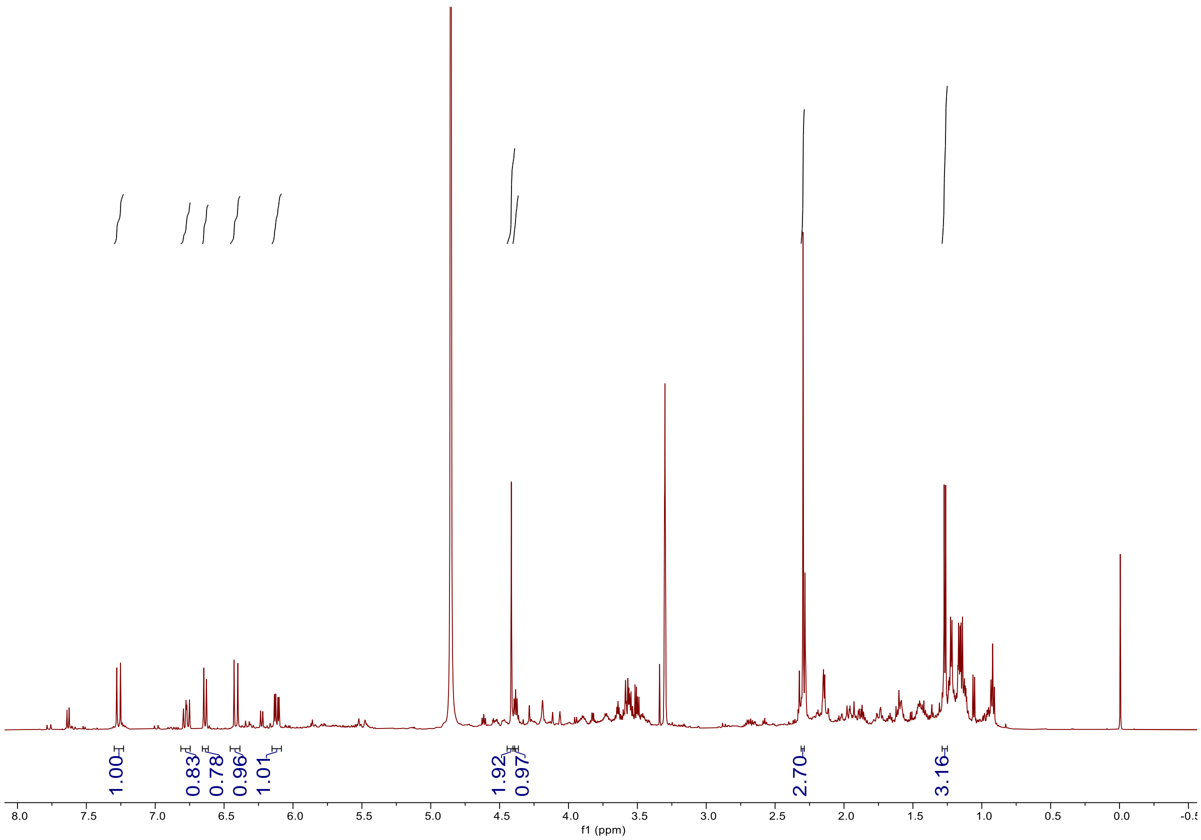

Figure S5: ^{1}H-NMR (600 MHz, CD_3OD) spectrum of compound 1

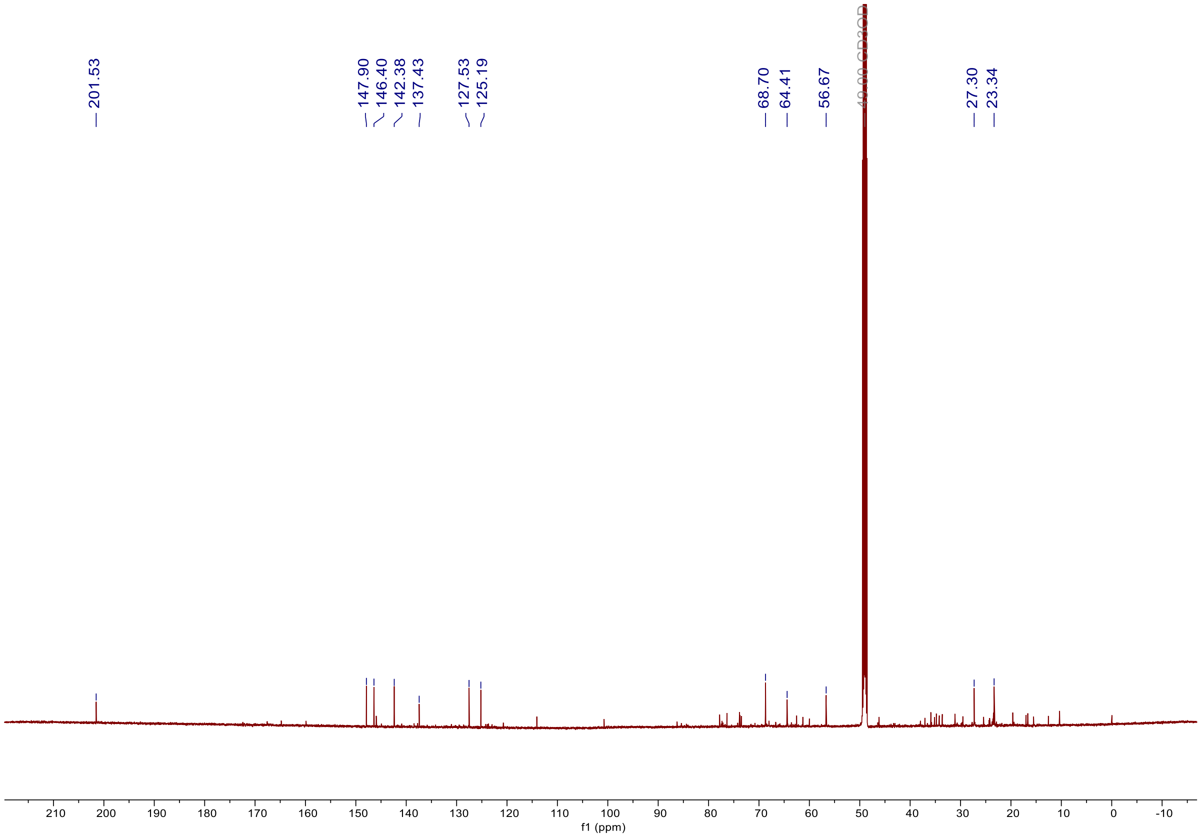

Figure S6: ^{13}C-NMR (150 MHz, CD_3OD) spectrum of compound 1

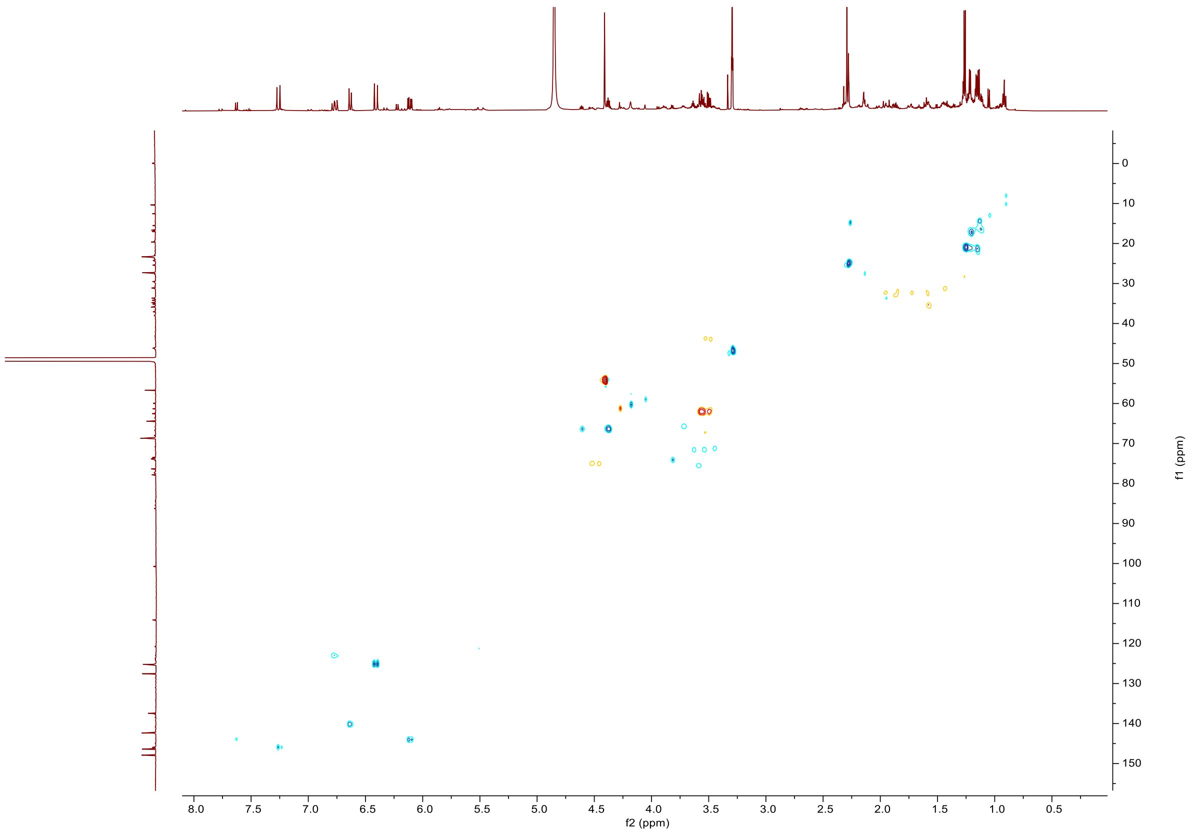

Figure S7: HSQC spectrum of compound 1

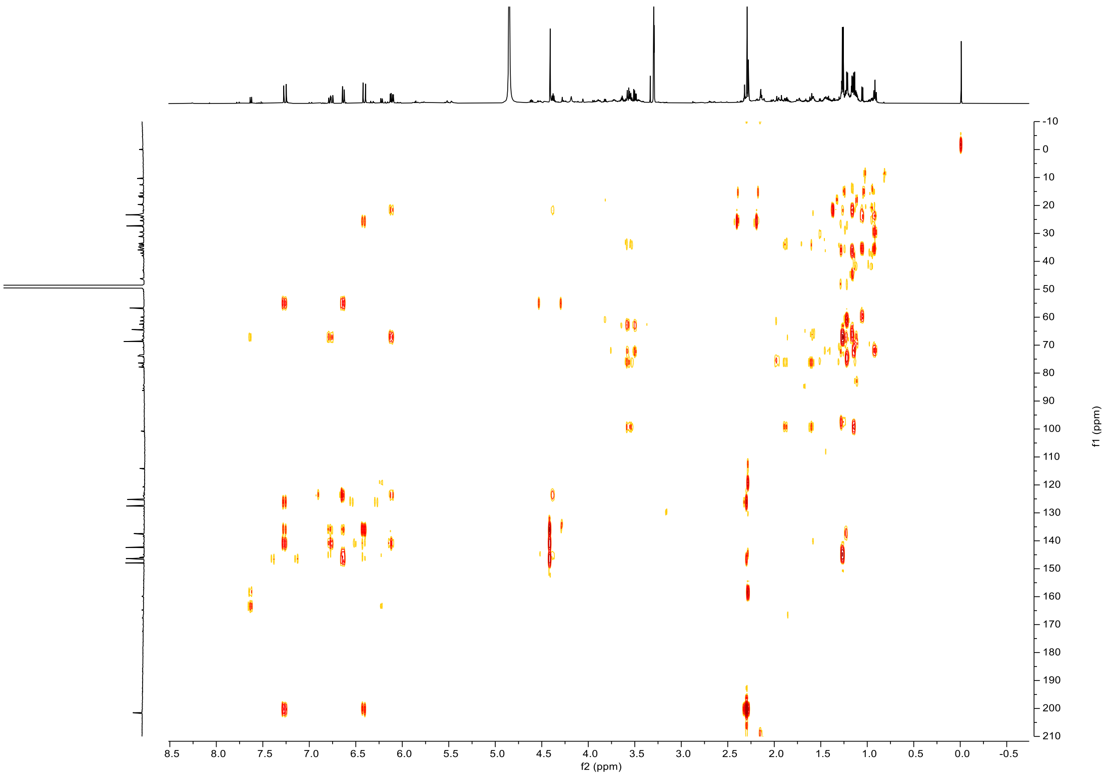

Figure S8: HMBC spectrum of compound 1

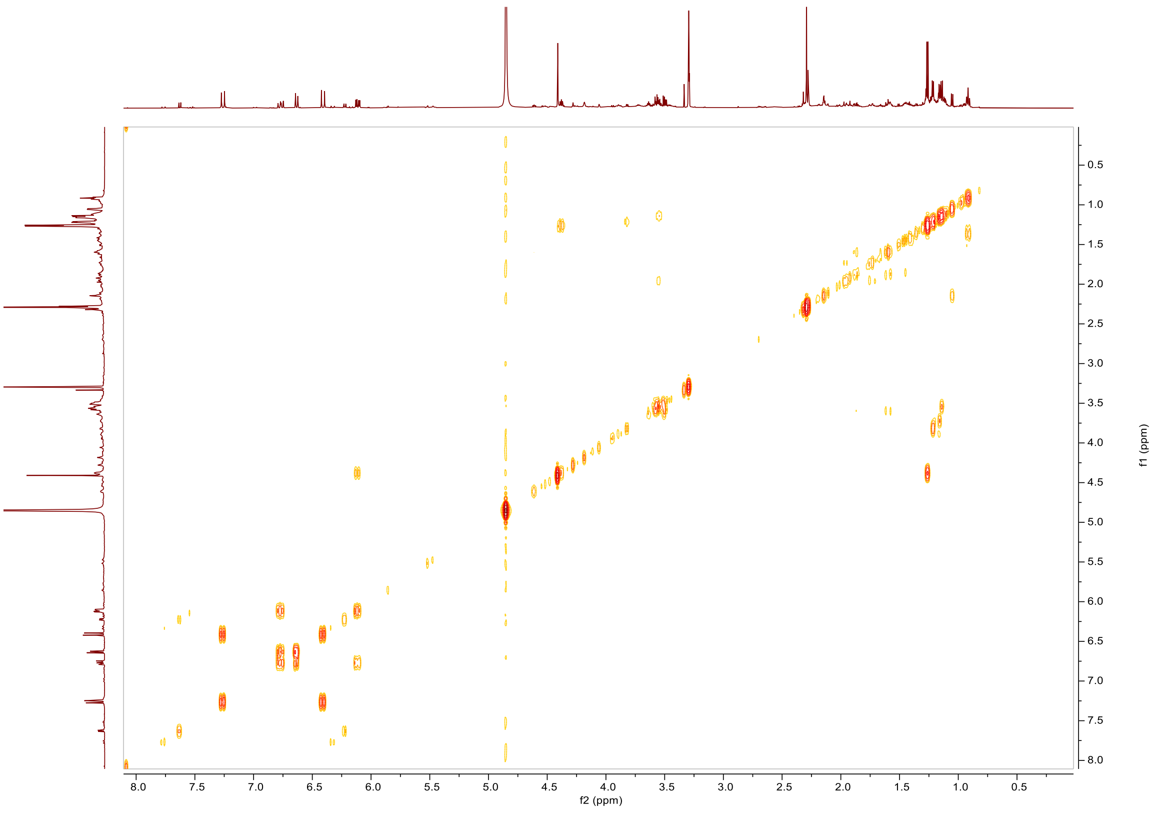

Figure S9: ^1H-^1H COSY spectrum of compound 1

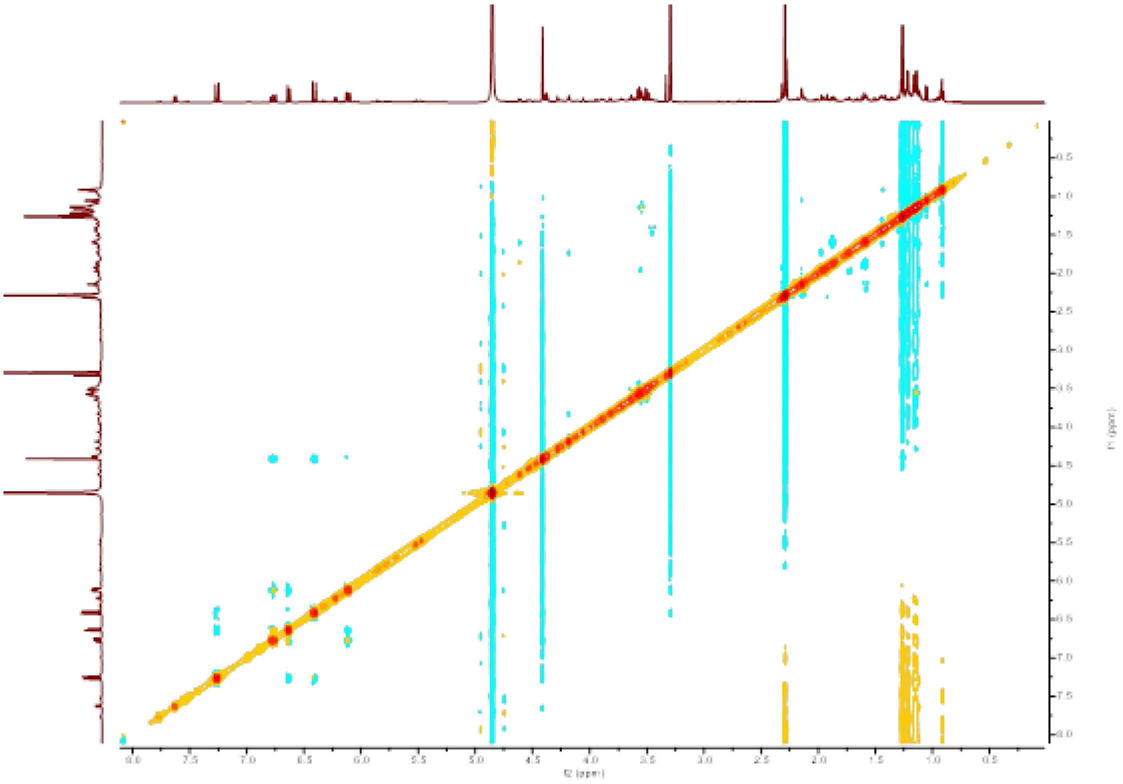

Figure S10: NOESY spectrum of compound 1

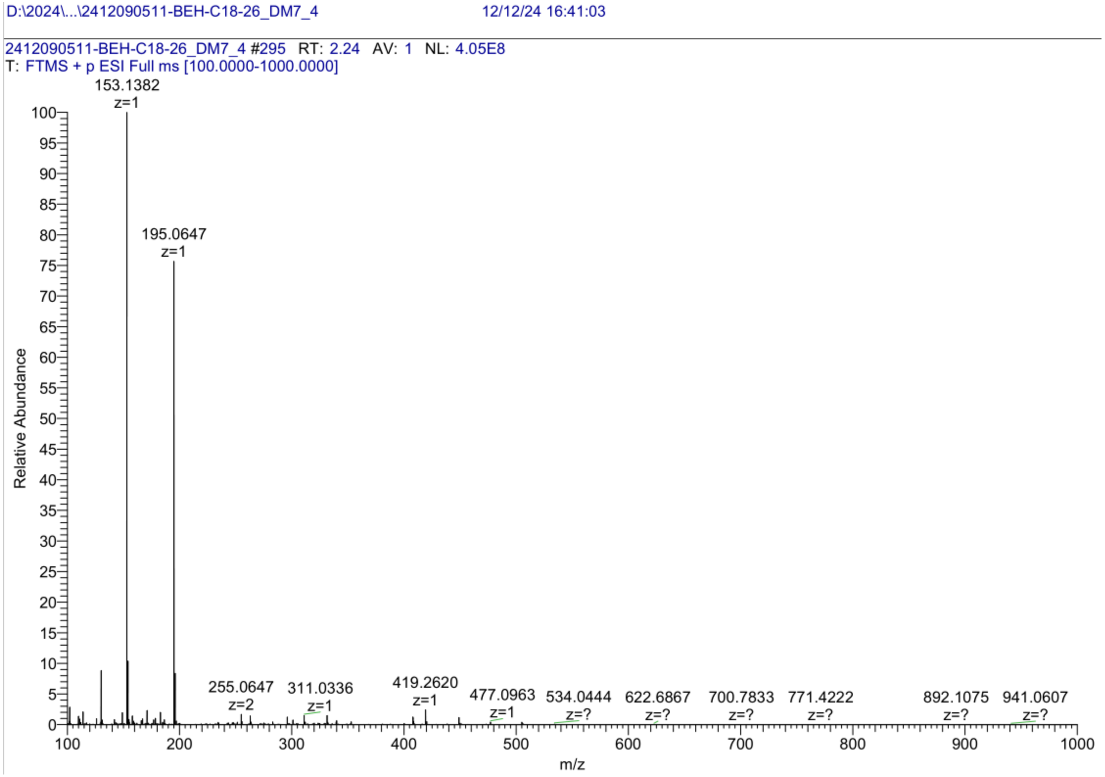

Figure S11: HRESIMS spectrum of compound 2

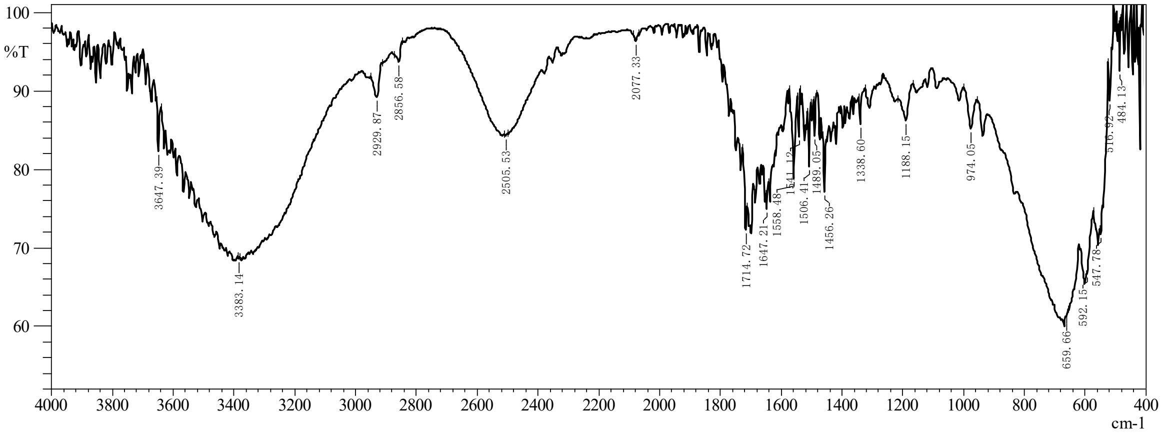

Figure S12: IR spectrum of compound 2

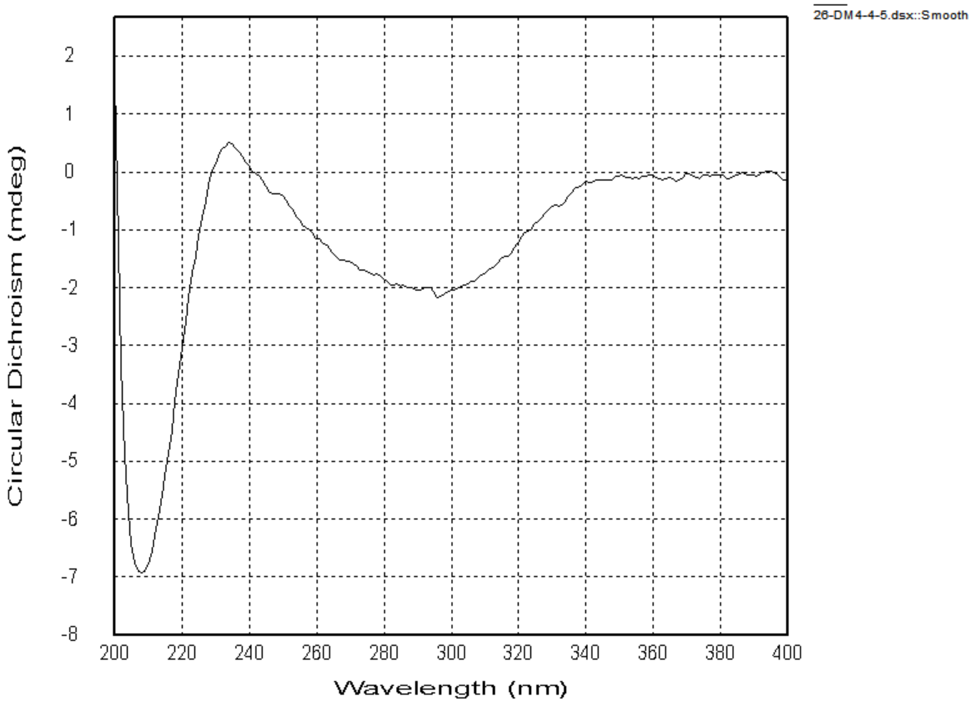

Figure S13: ECD spectra of compound 2 in MeOH

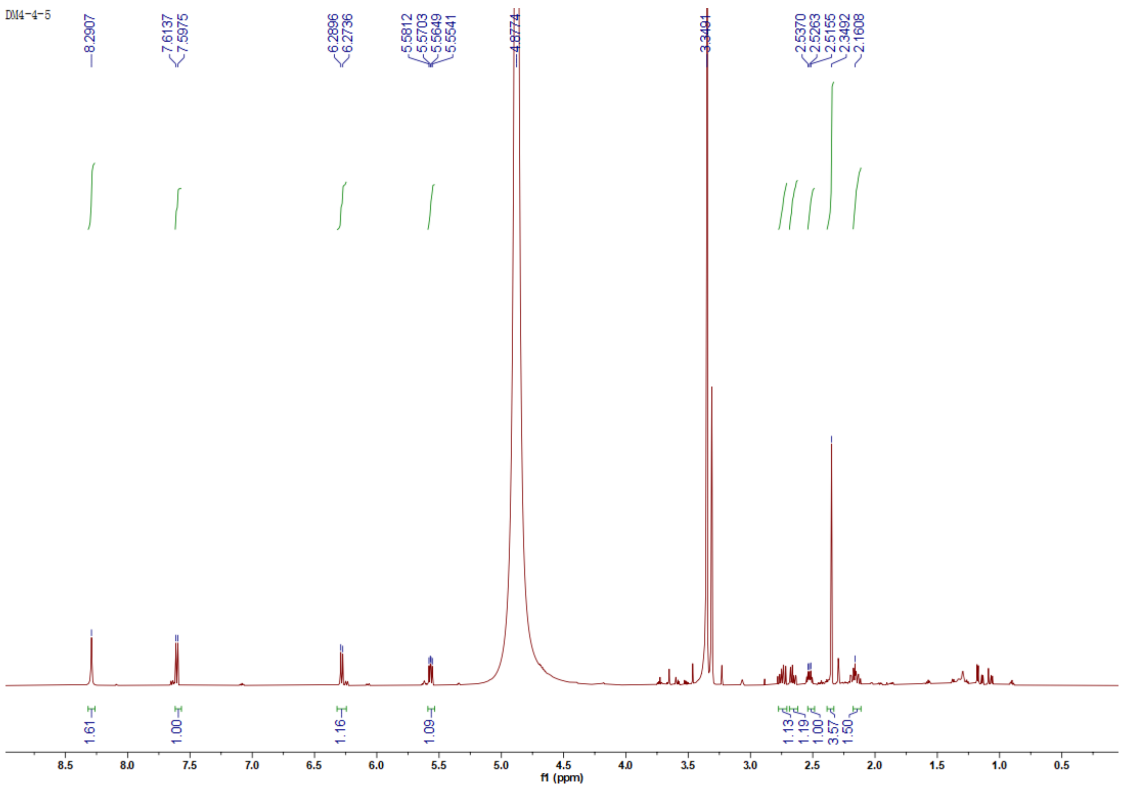

Figure S14: ^{1}H-NMR (600 MHz, CD_3OD) spectrum of compound 2

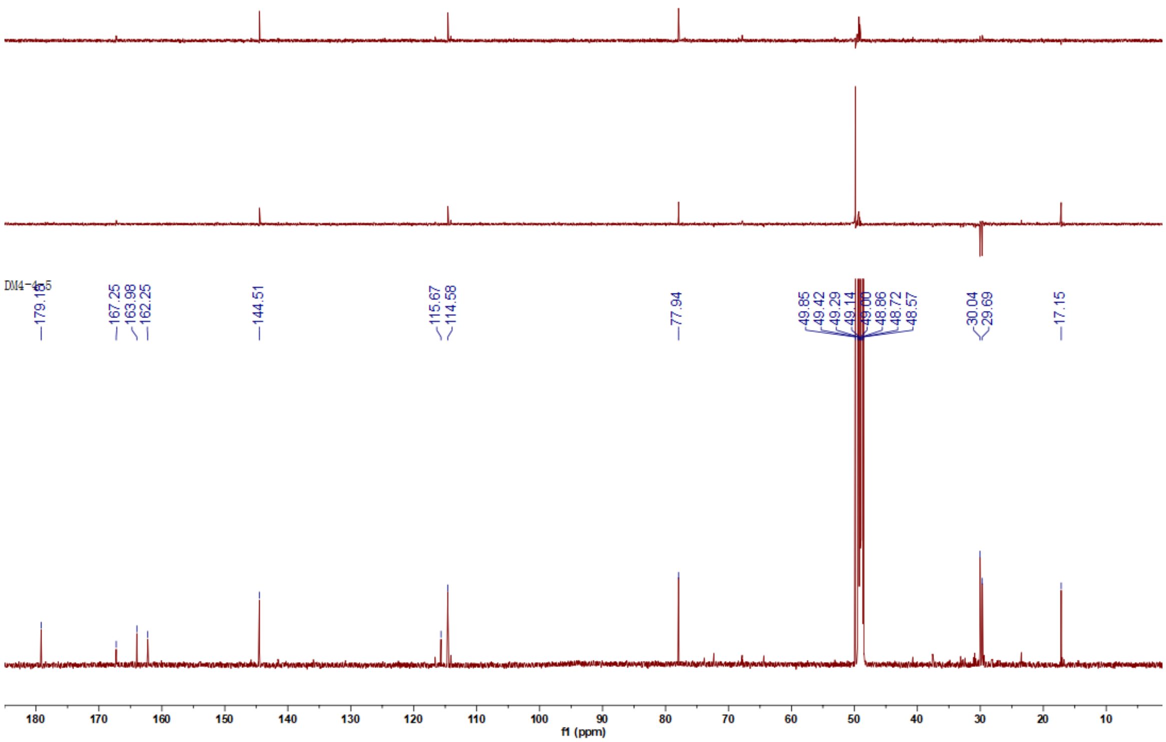

Figure S15: ^{13}C-NMR and DEPT (150 MHz, CD_3OD) spectrum of compound 2

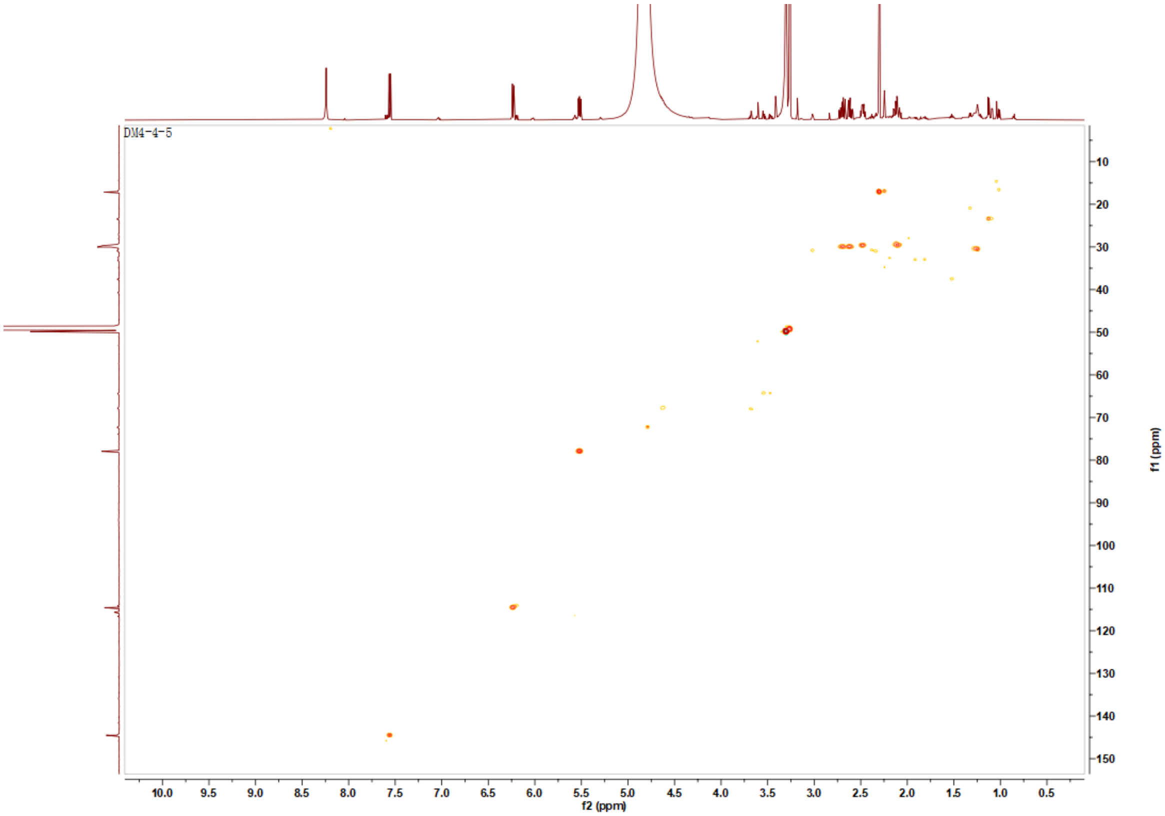

Figure S16: HSQC spectrum of compound 2

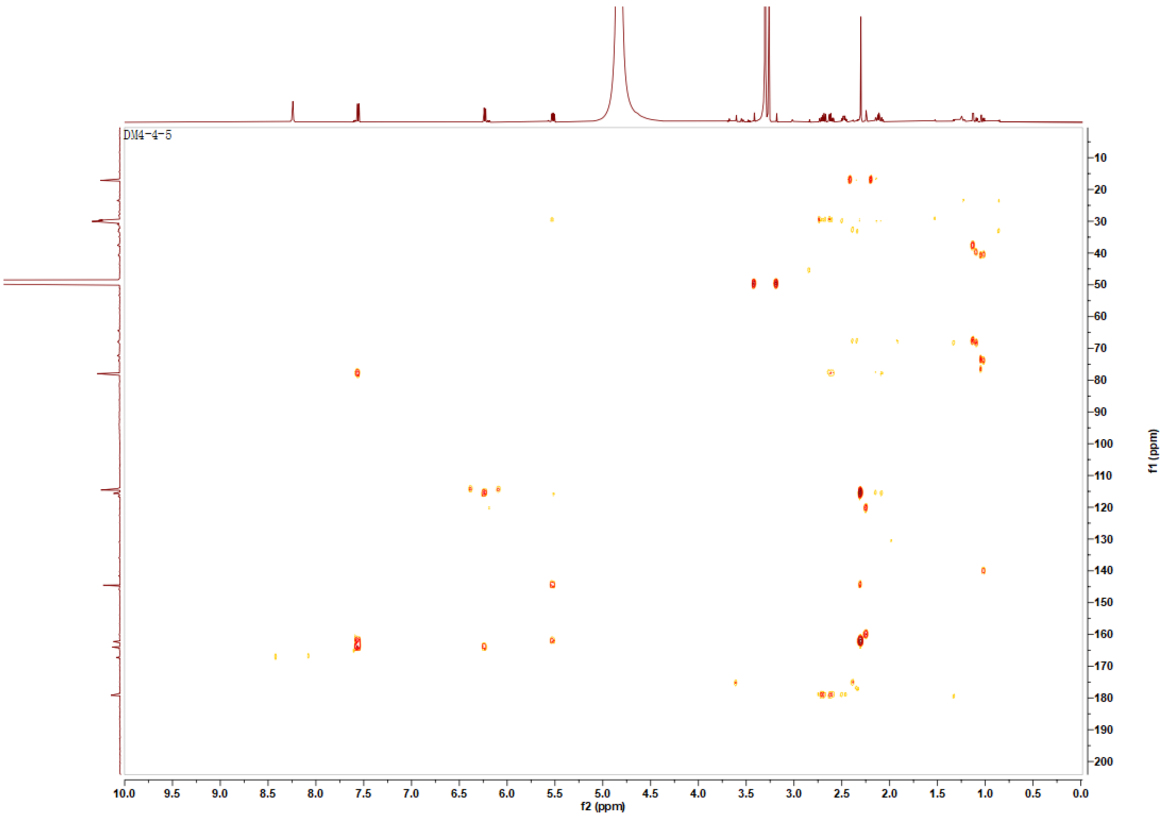

Figure S17: HMBC spectrum of compound 2

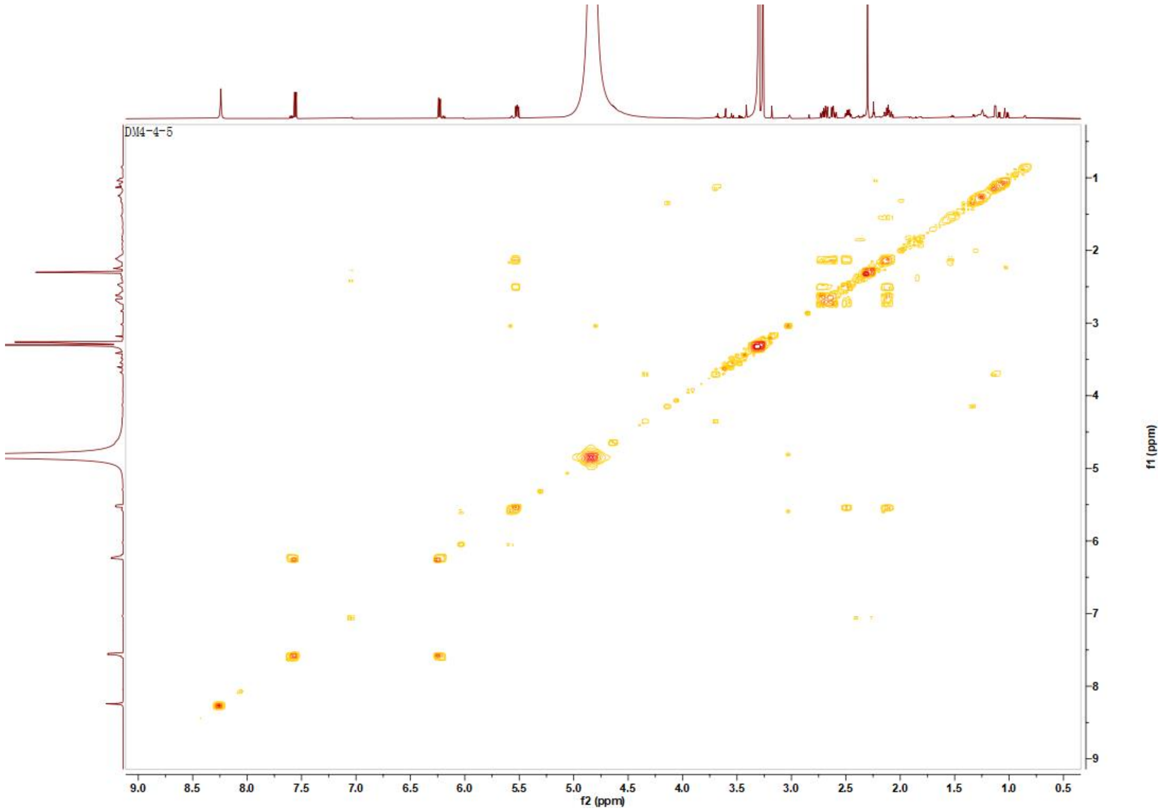

Figure S18: ^{1}H-^{1}H COSY spectrum of compound 2

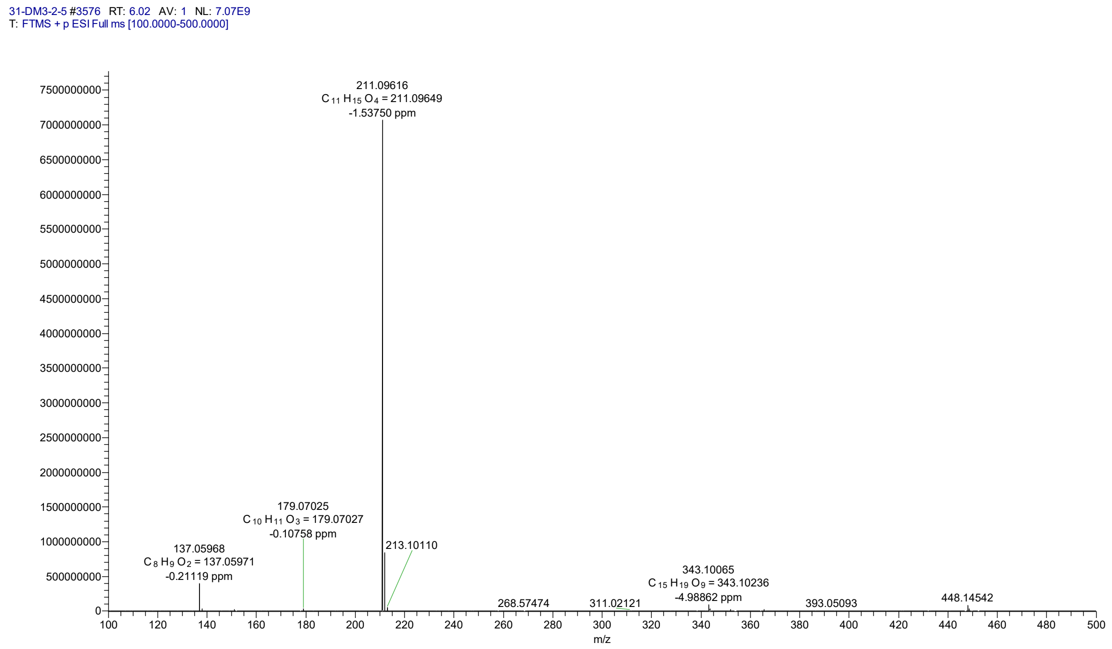

Figure S19: HRESIMS spectrum of compound 3

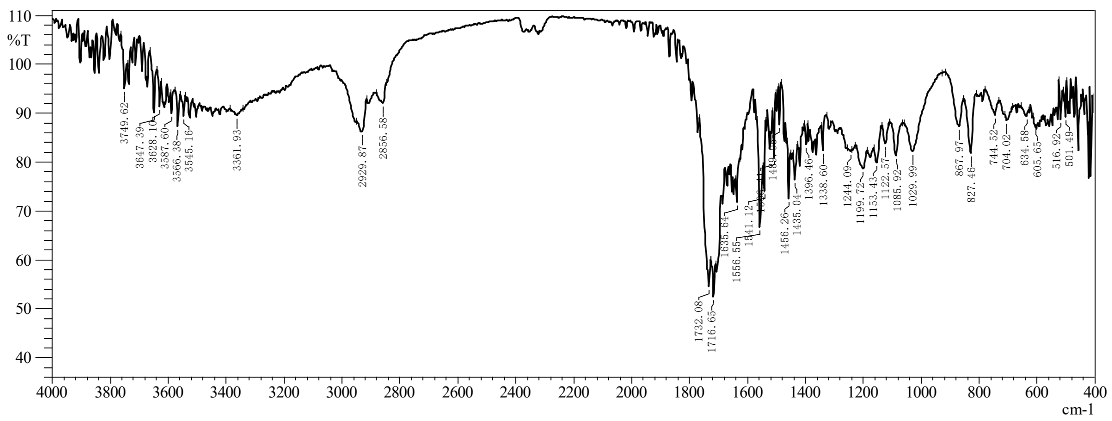

Figure S20: IR spectrum of compound 3

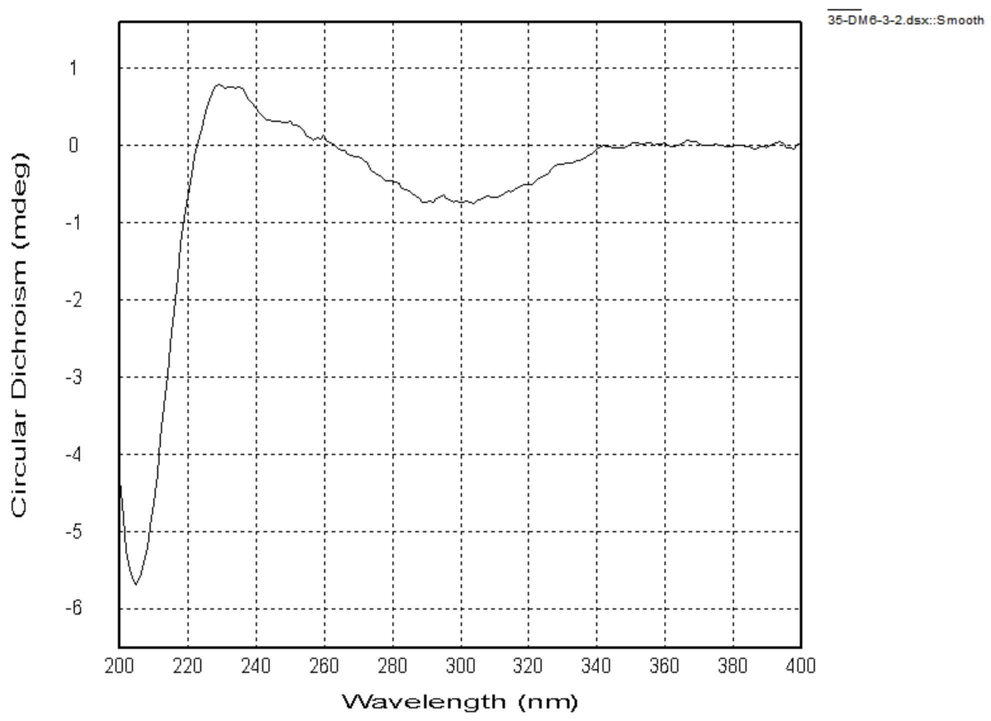

Figure S21: ECD spectra of compound 4 in MeOH

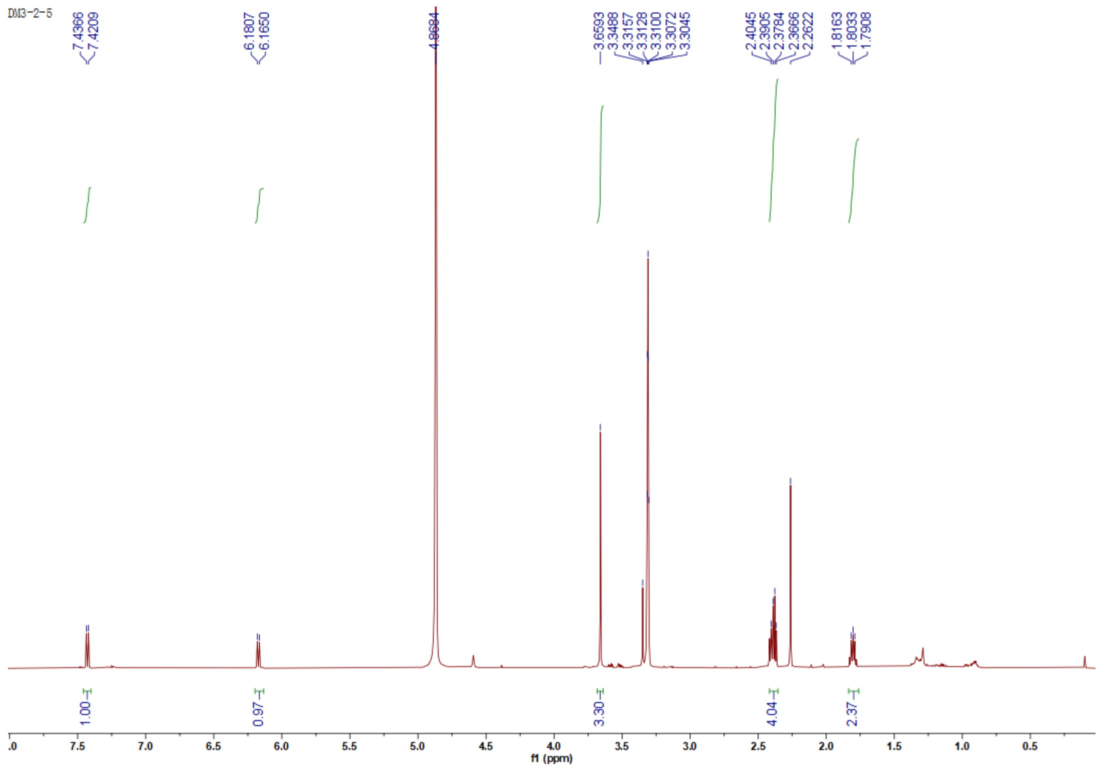

Figure S22: ^{1}H-NMR (600 MHz, CD_3OD) spectrum of compound 3

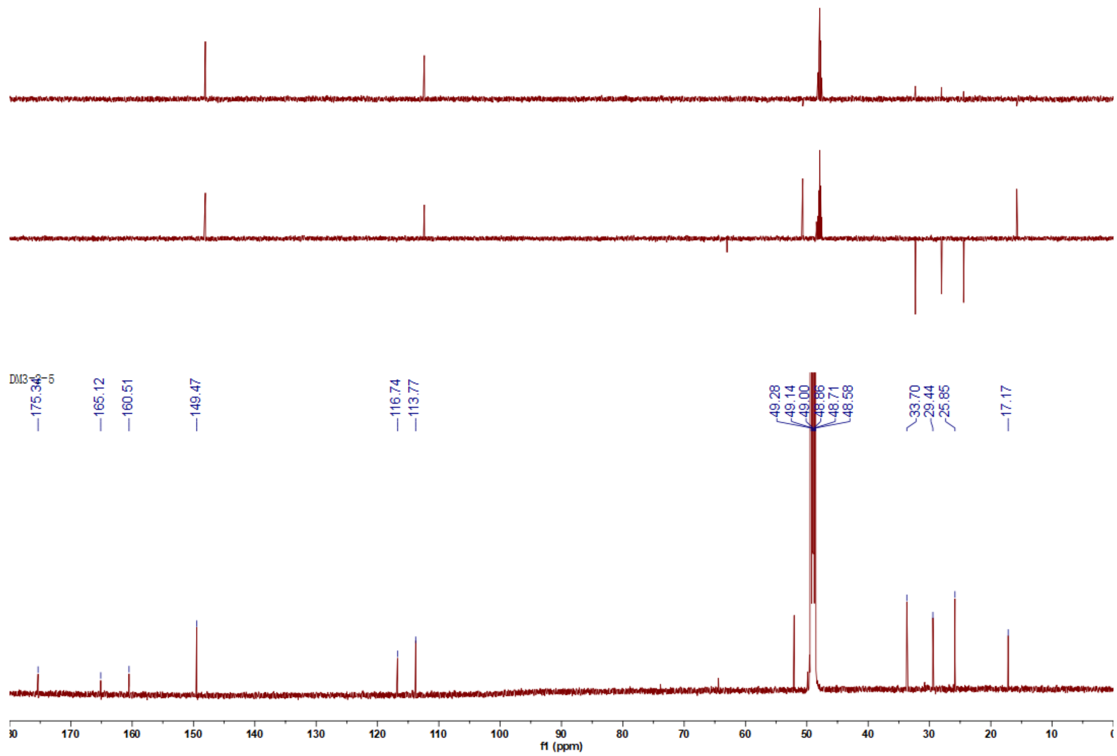

Figure S23: ^{13}C-NMR and DEPT (150 MHz, CD_3OD) spectrum of compound 3

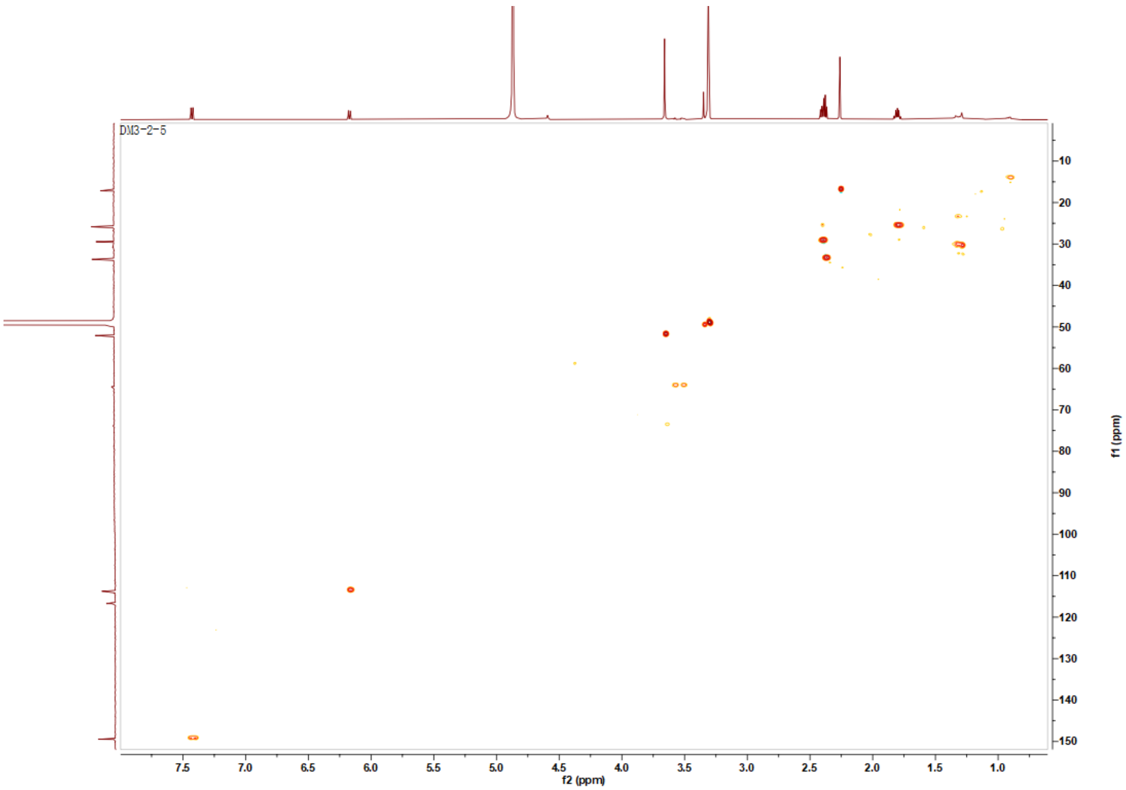

Figure S24: HSQC spectrum of compound 3

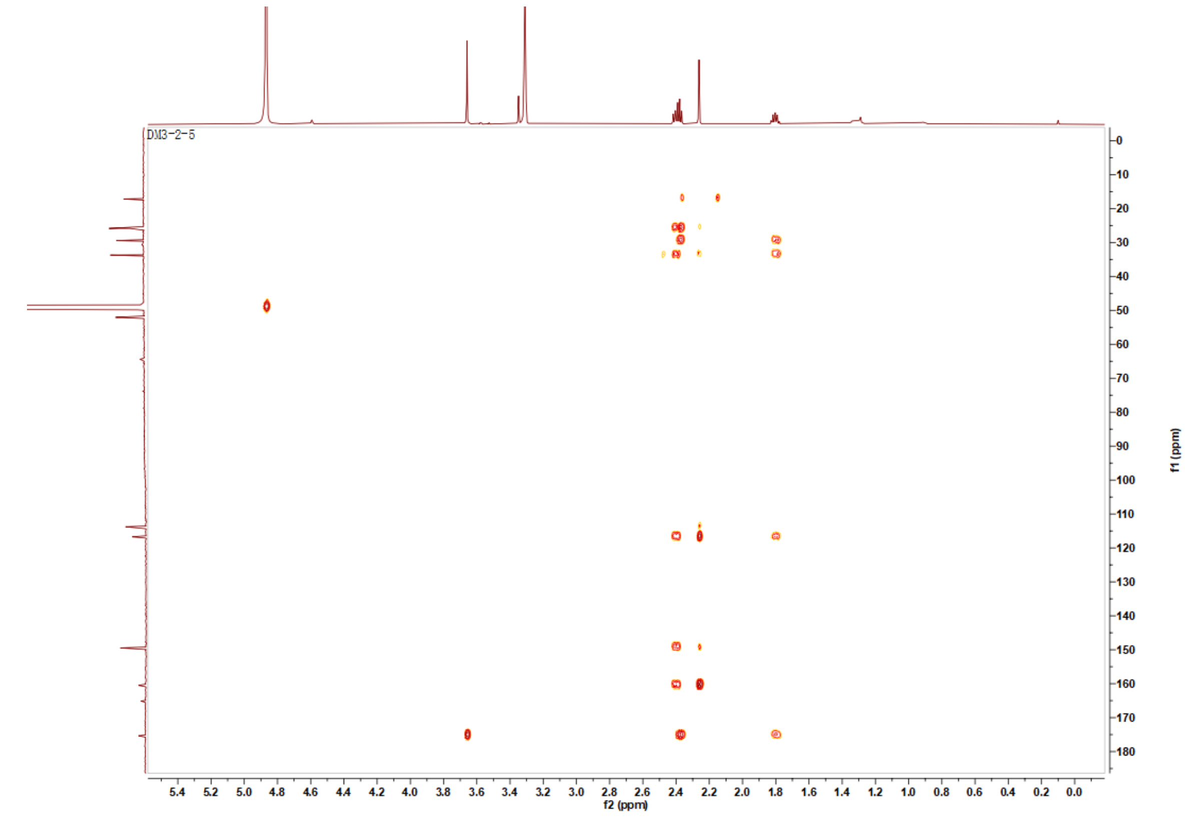

Figure S25: HMBC spectrum of compound 3

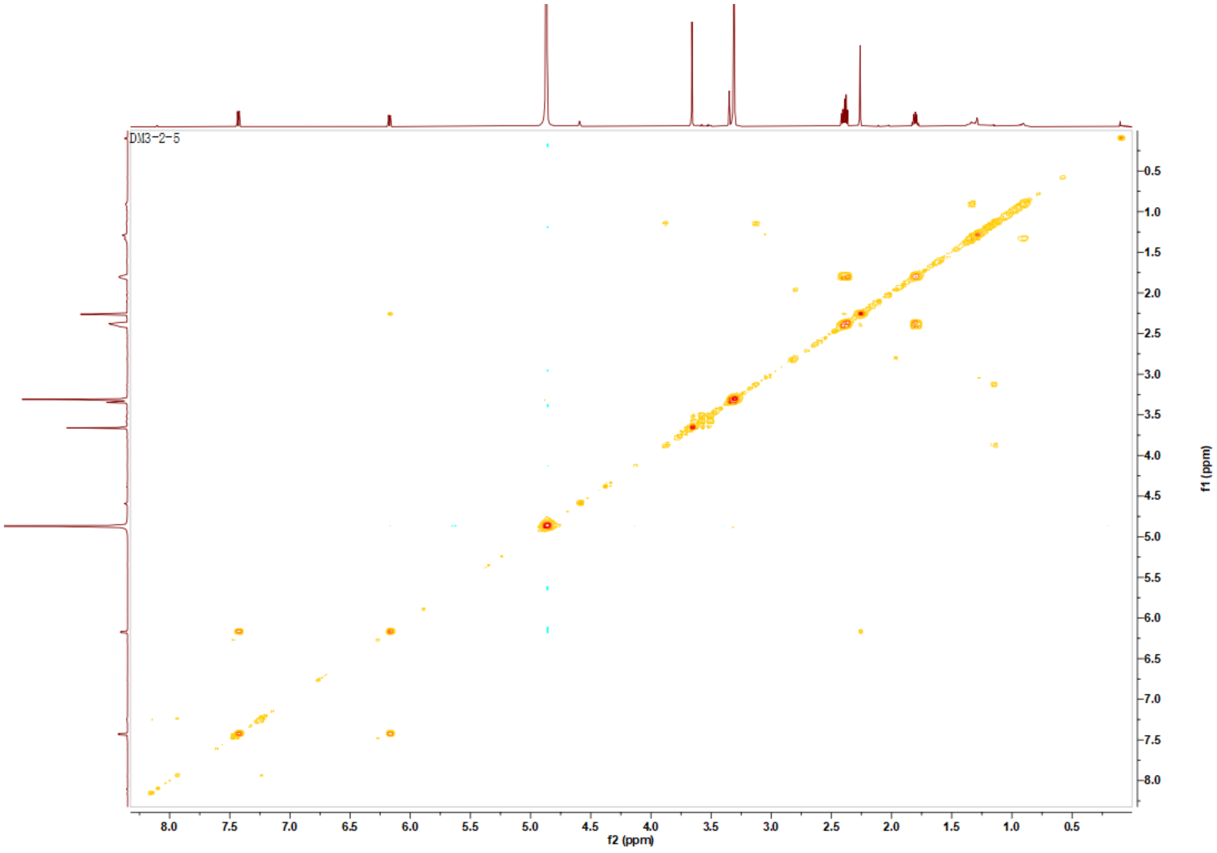

Figure S26: ¹H-¹H COSY spectrum of compound 3

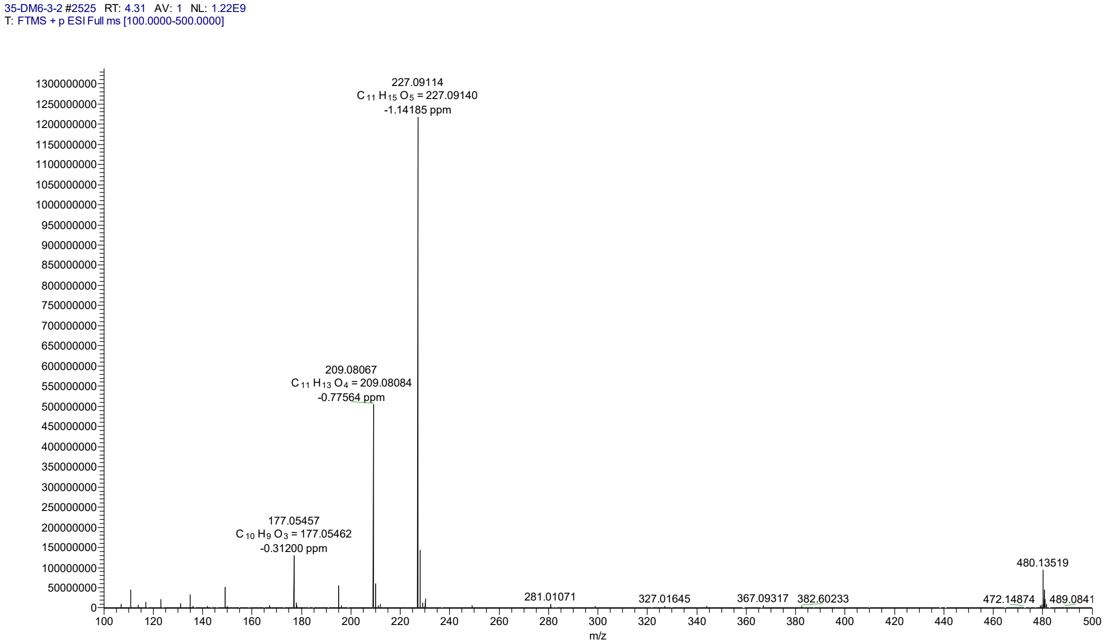

Figure S27: HRESIMS spectrum of compound 4

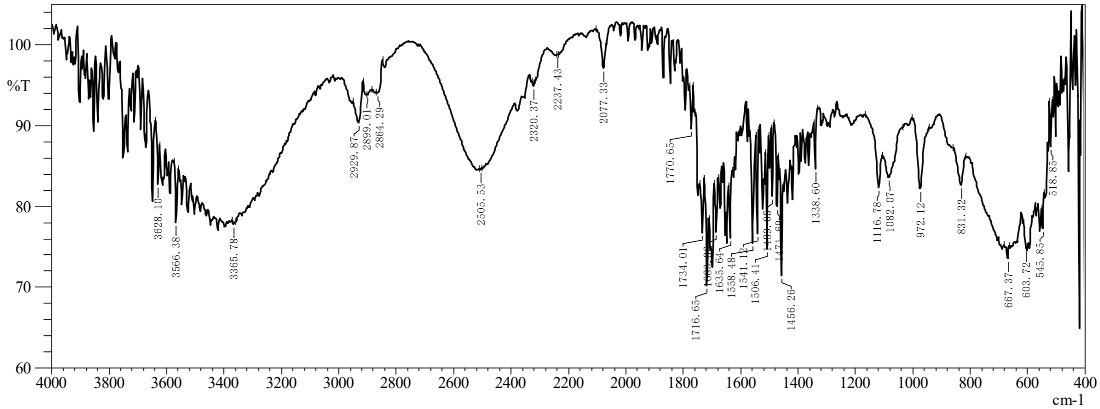

Figure S28: IR spectrum of compound 4

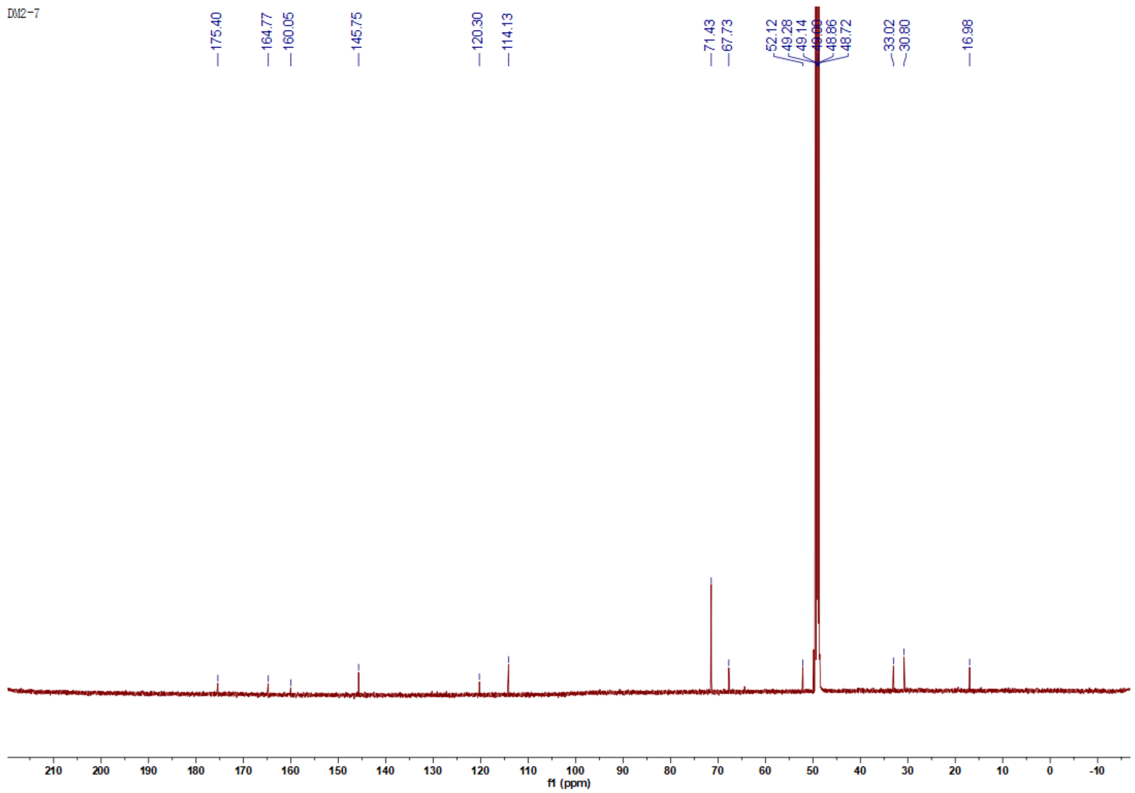

Figure S29: ^{1}H-NMR (600 MHz, CD_3OD) spectrum of compound 4

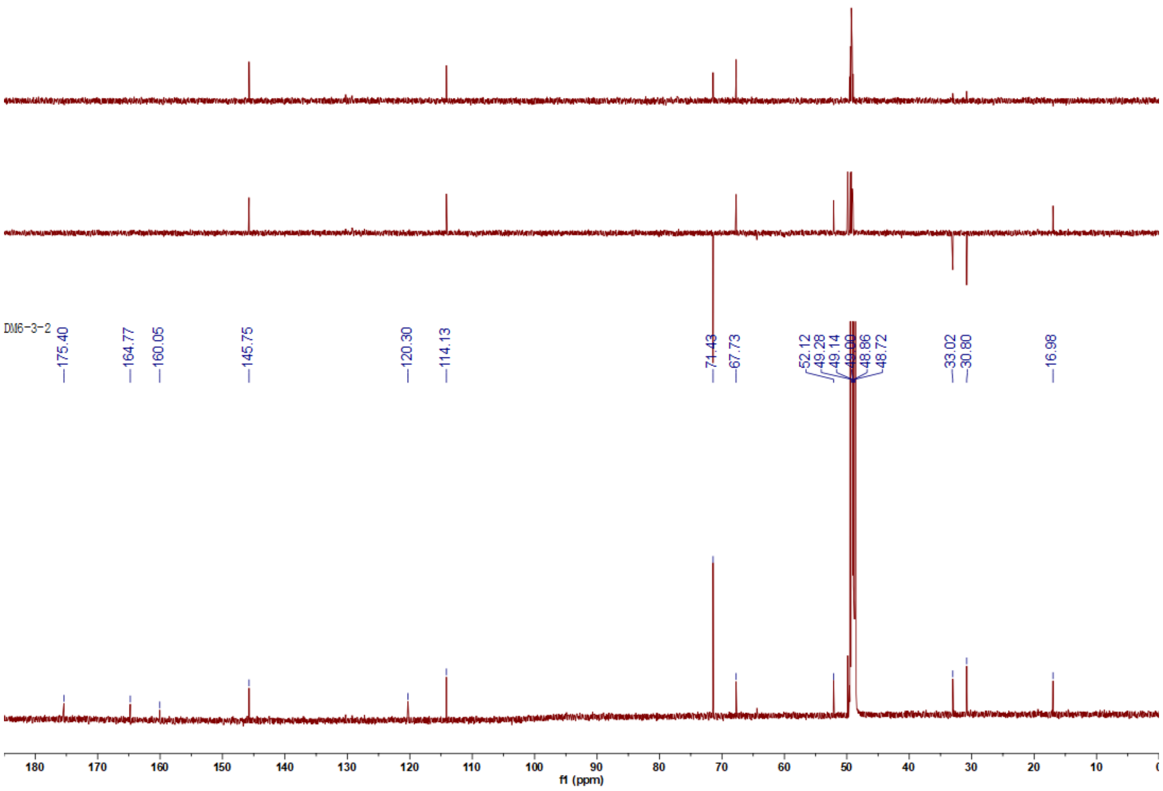

Figure S30: ^{13}C-NMR and DEPT (150 MHz, CD_3OD) spectrum of compound 4

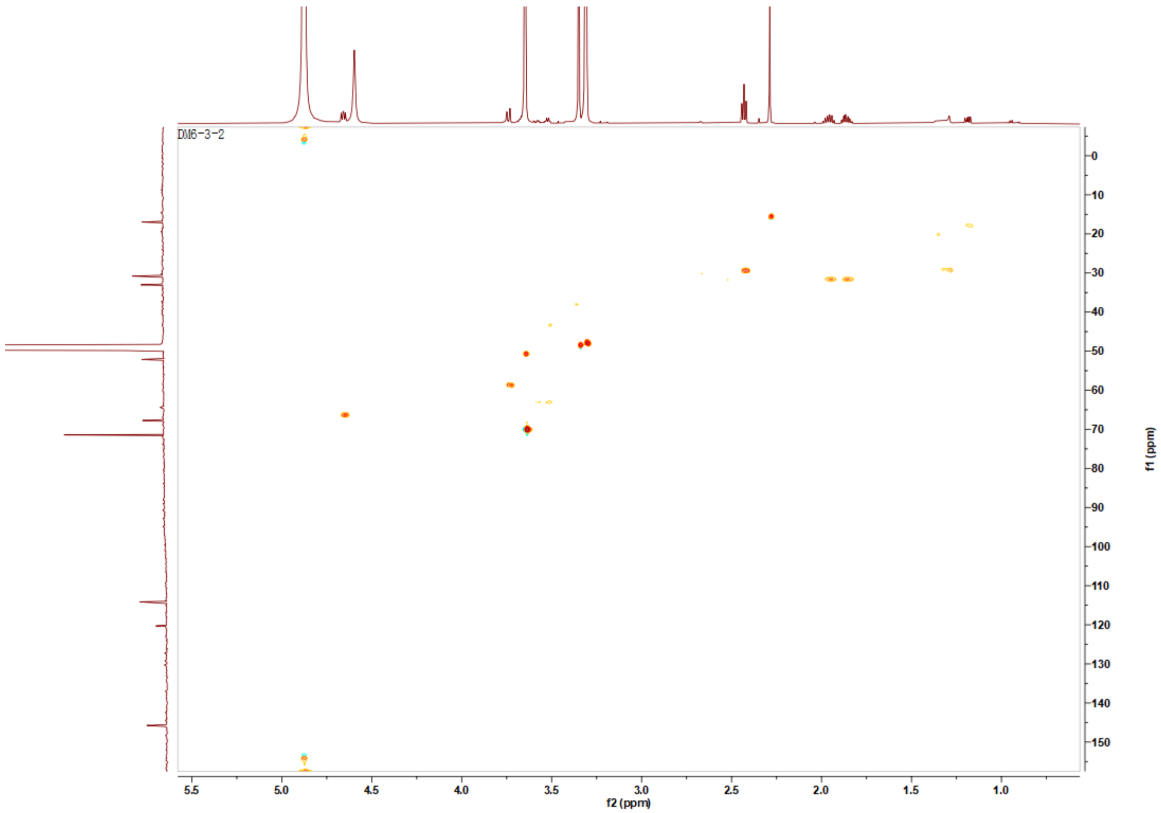

Figure S31: HSQC spectrum of compound 4

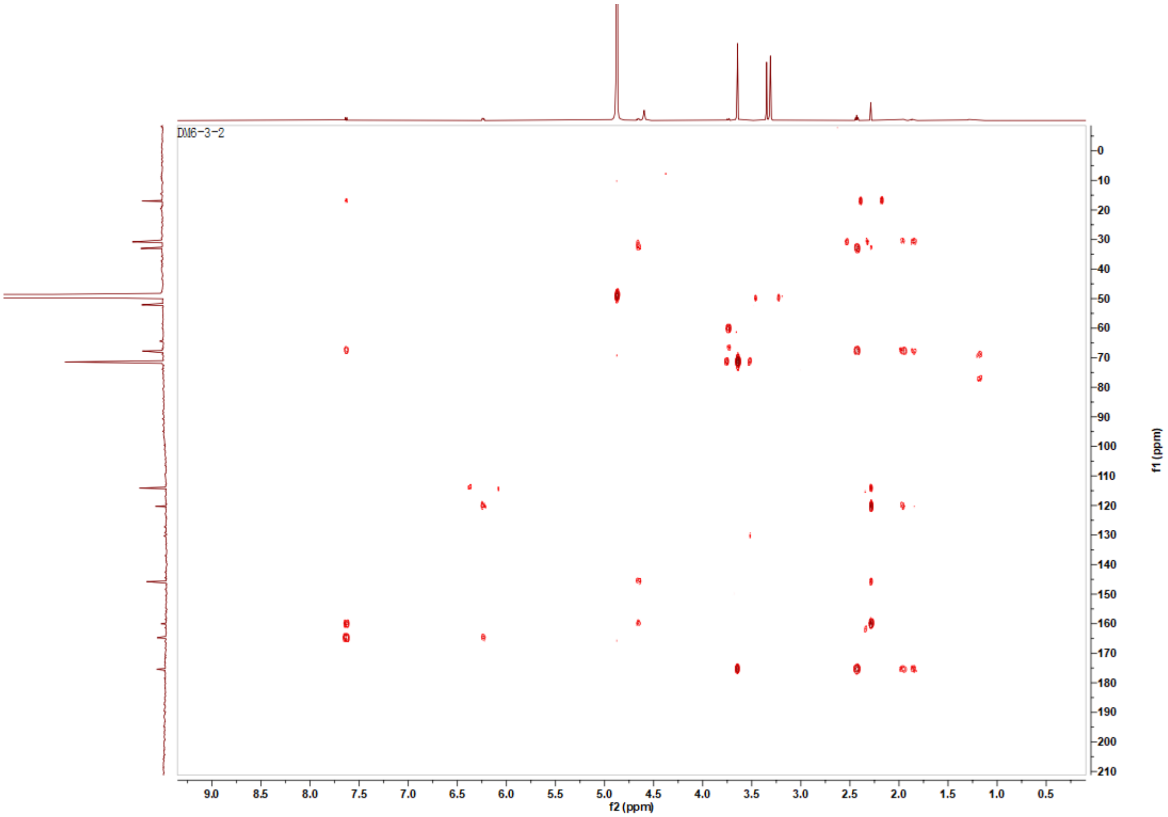

Figure S32: HMBC spectrum of compound 4

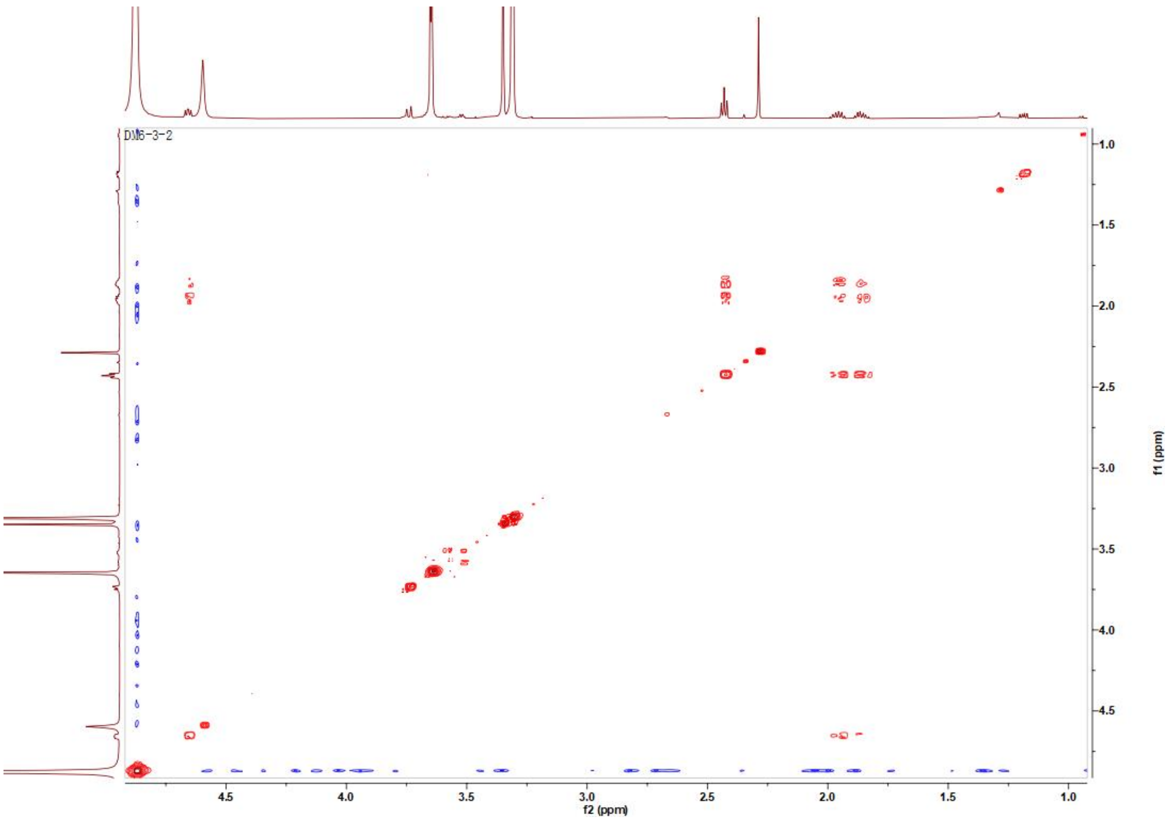

Figure S33: ^{1}H-^{1}H COSY spectrum of compound 4

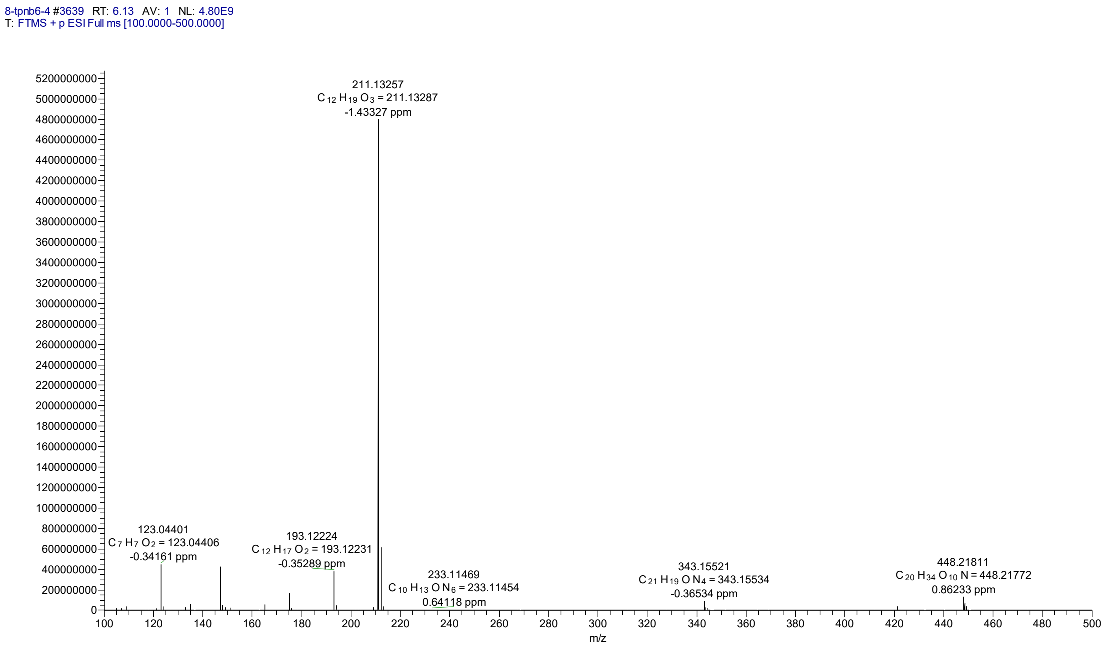

Figure S34: HRESIMS spectrum of compound 5

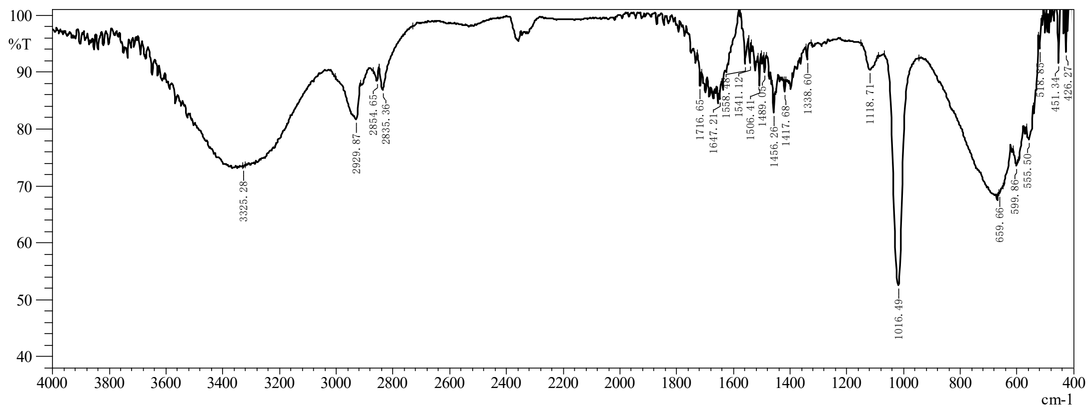

Figure S35: IR spectrum of compound 5

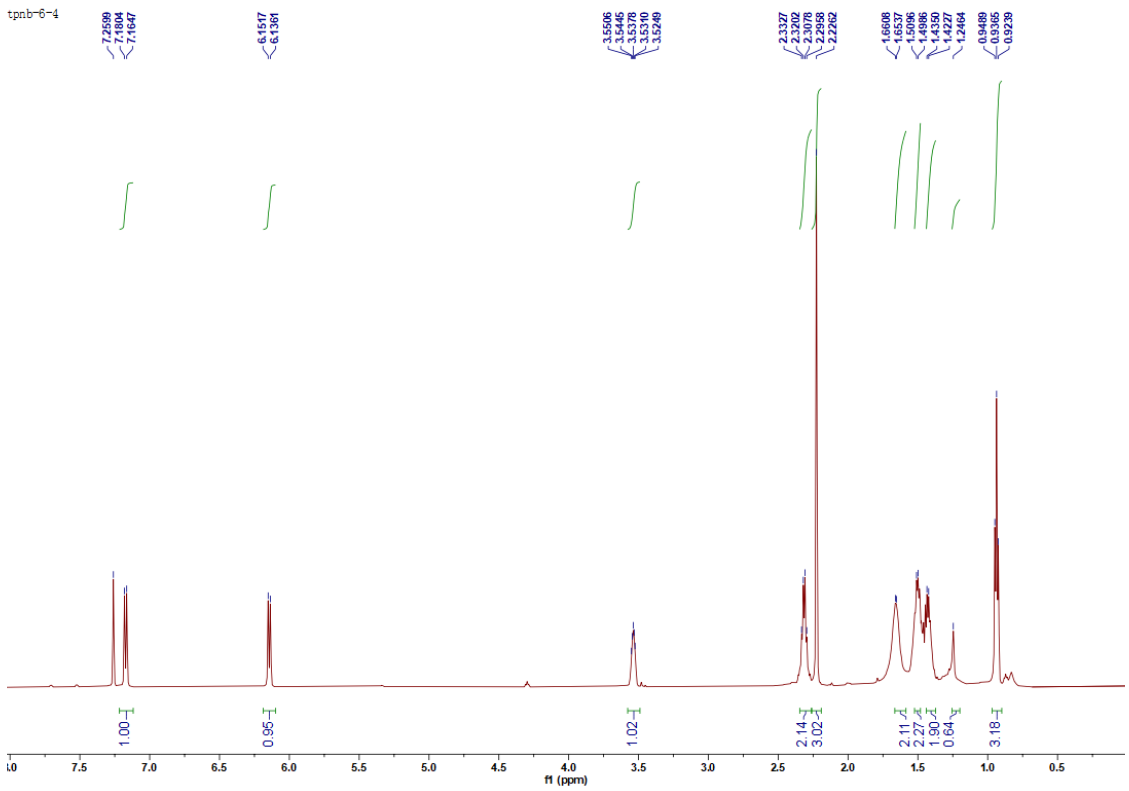

Figure S36: ^{1}H-NMR (600 MHz, $CDCl_3$) spectrum of compound 5

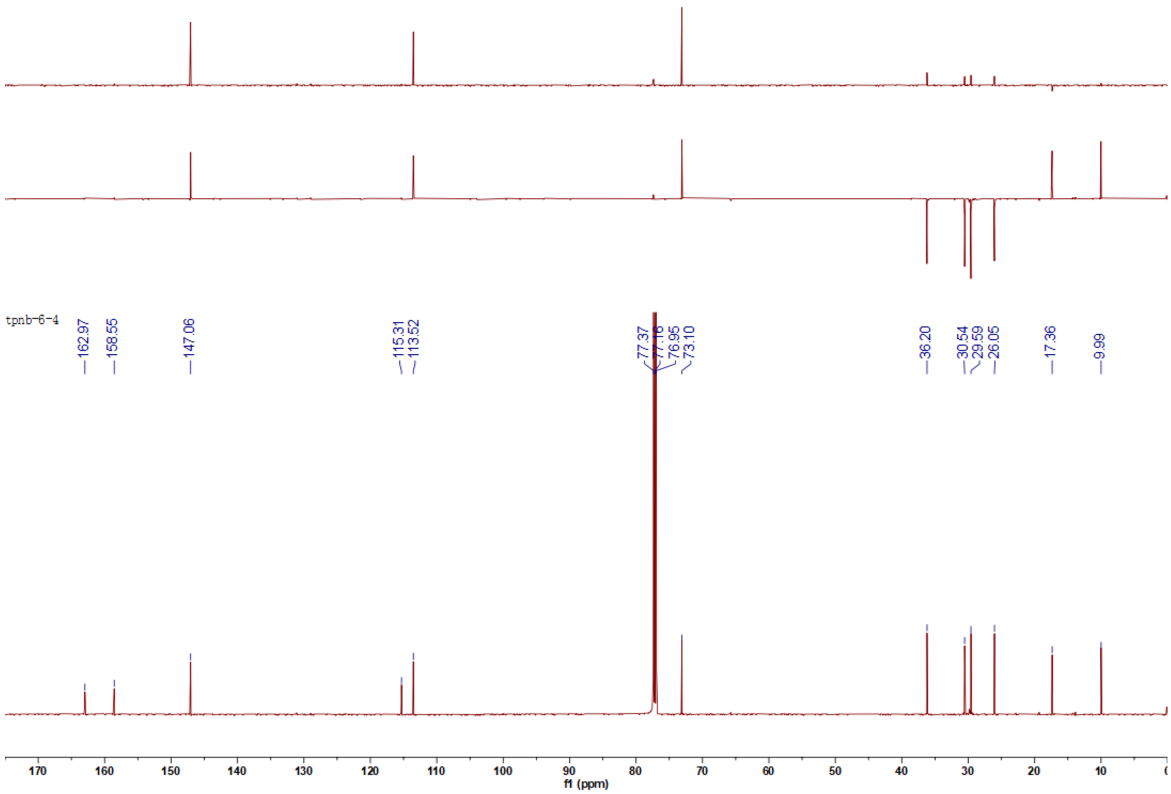

Figure S37: ^{13}C-NMR and DEPT (150 MHz, $CDCl_3$) spectrum of compound 5

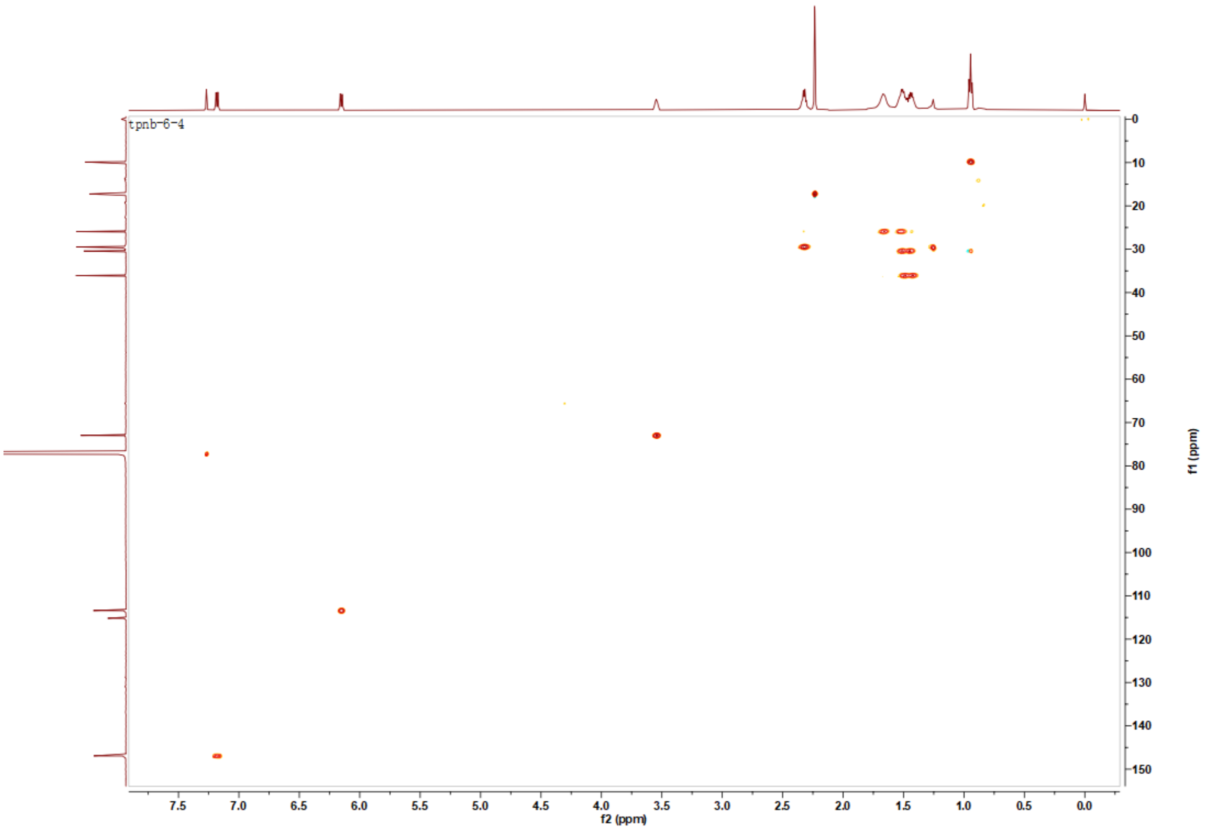

Figure S38: HSQC spectrum of compound 5

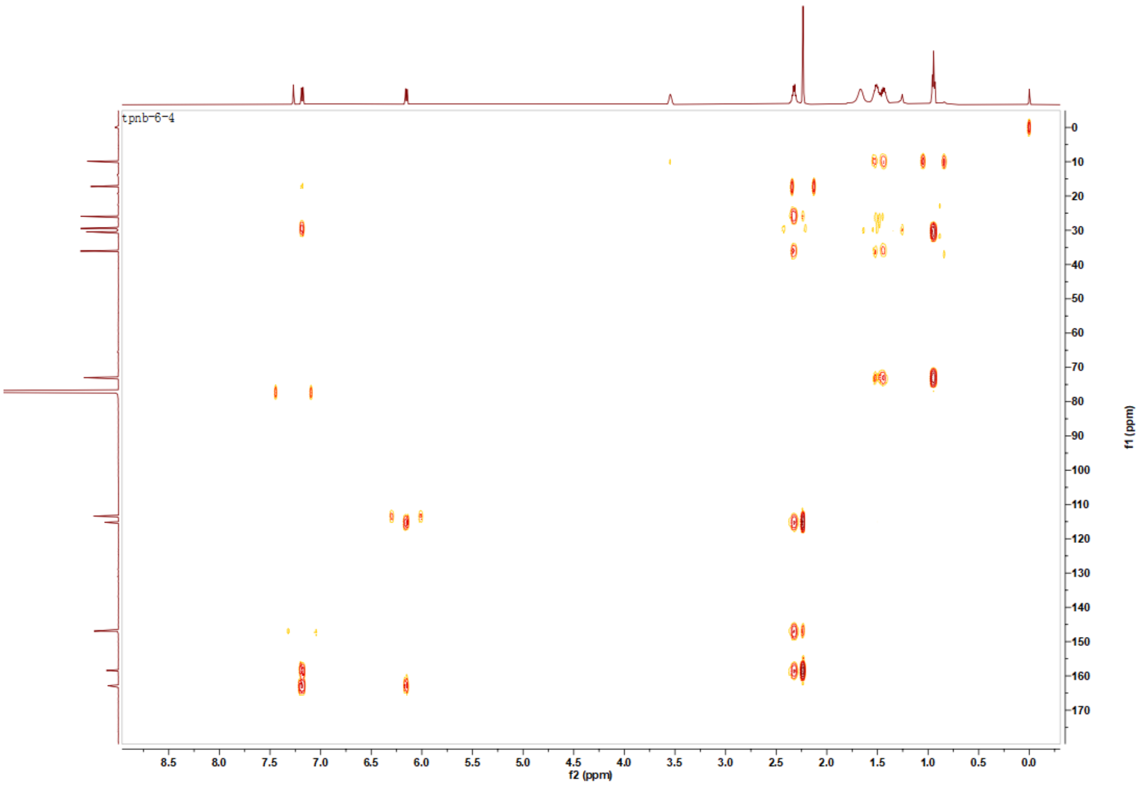

Figure S39: HMBC spectrum of compound 5

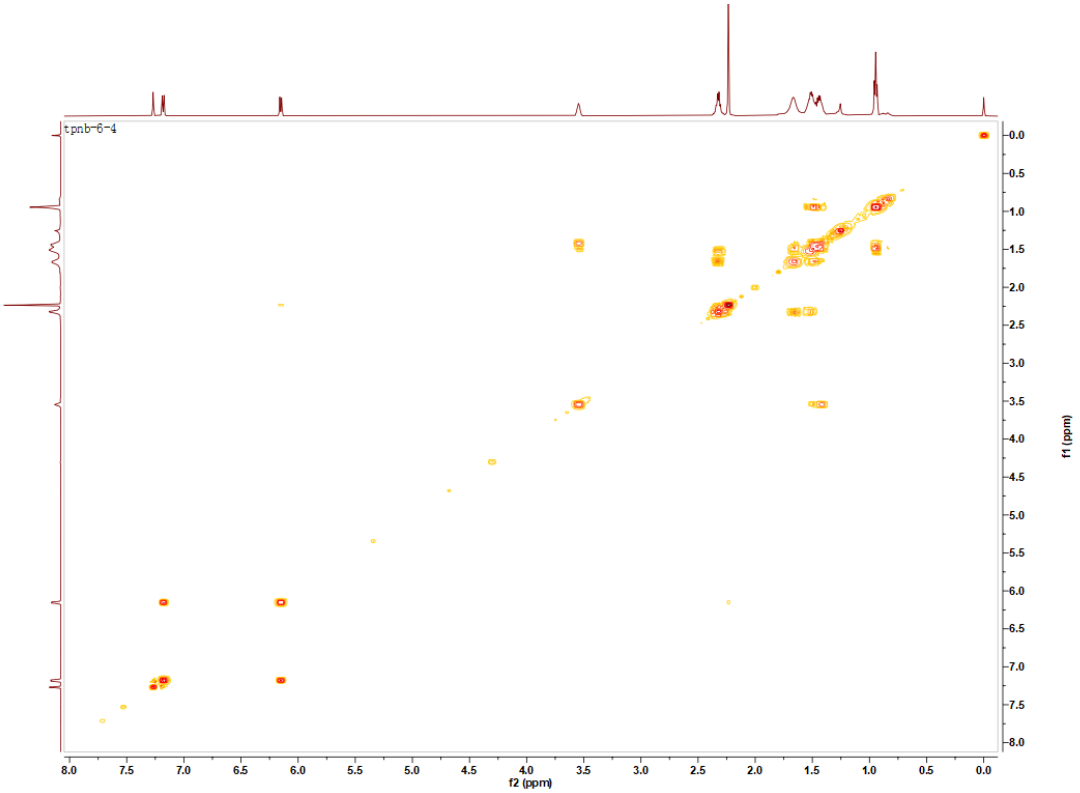

Figure S40: ^{1}H-^{1}H COSY spectrum of compound 5

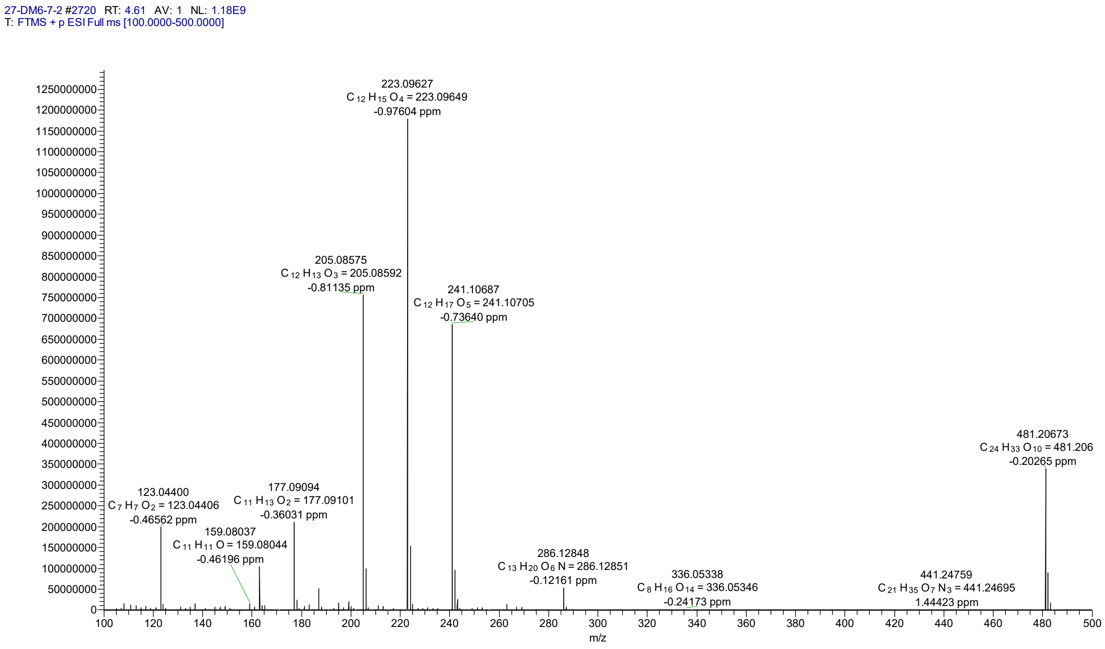

Figure S41: HRESIMS spectrum of compound 6

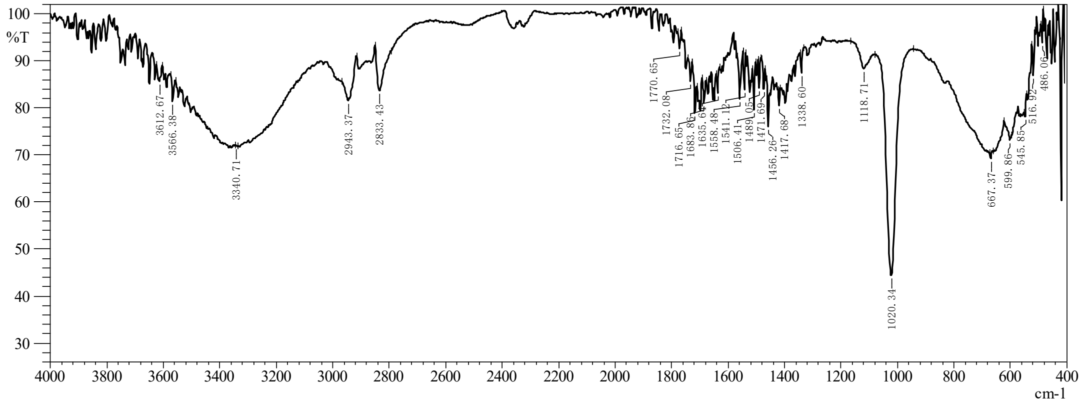

Figure S42: IR spectrum of compound 6

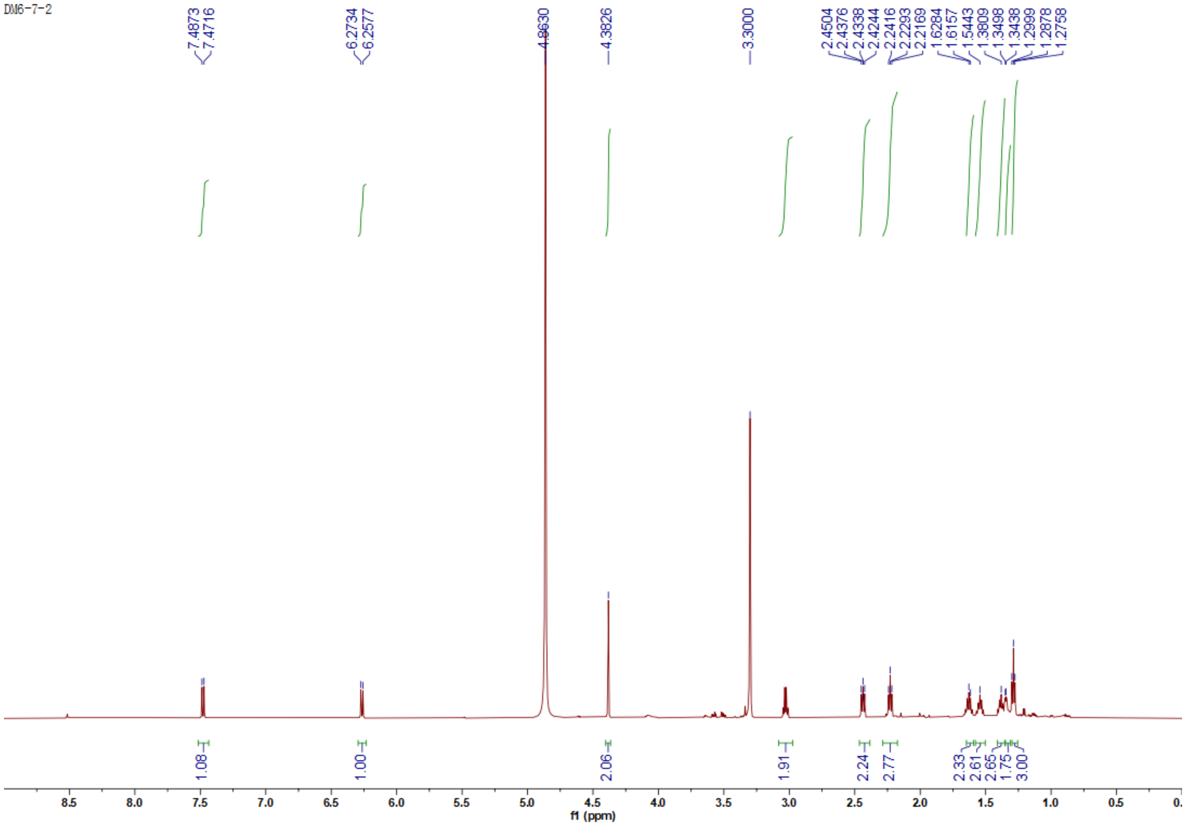

Figure S43: ^{1}H-NMR (600 MHz, CD3OD) spectrum of compound 6

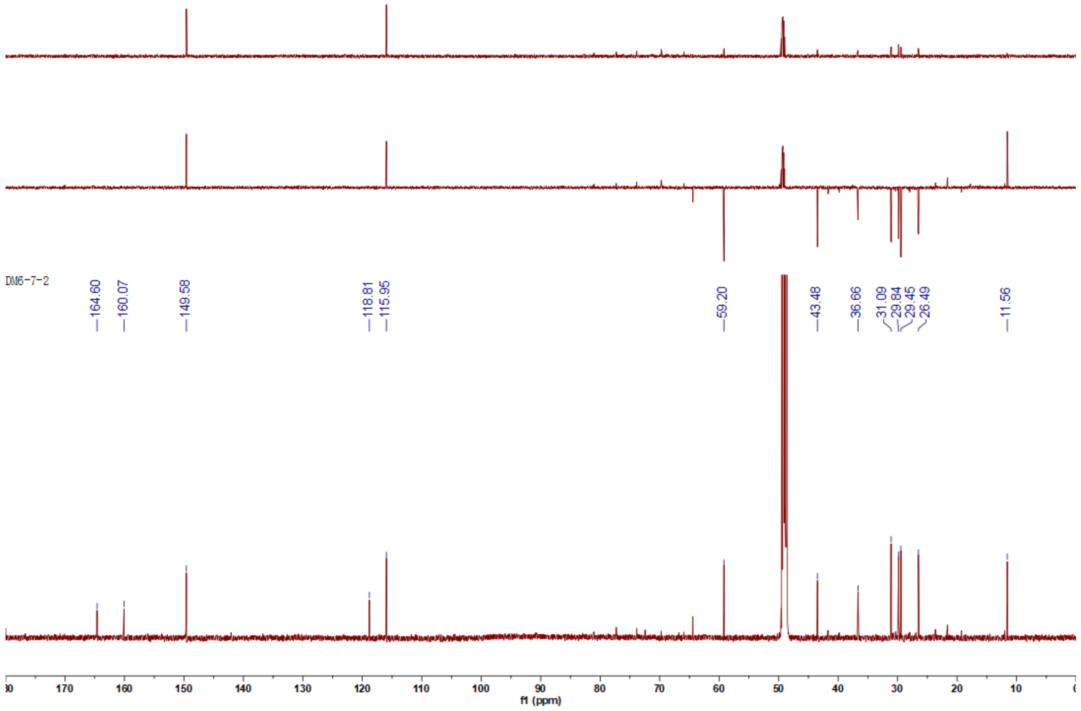

Figure S44: ^{13}C-NMR and DEPT (150 MHz, CD3OD) spectrum of compound 6

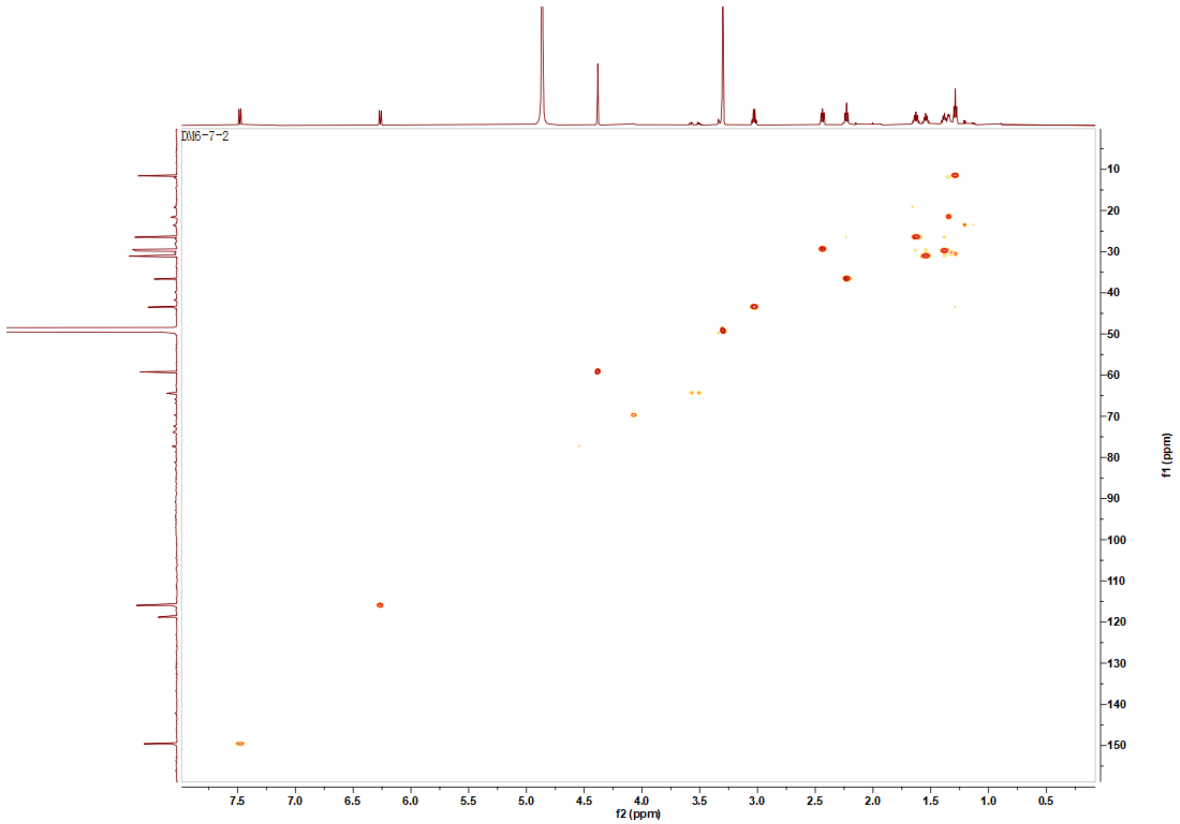

Figure S45: HSQC spectrum of compound 6

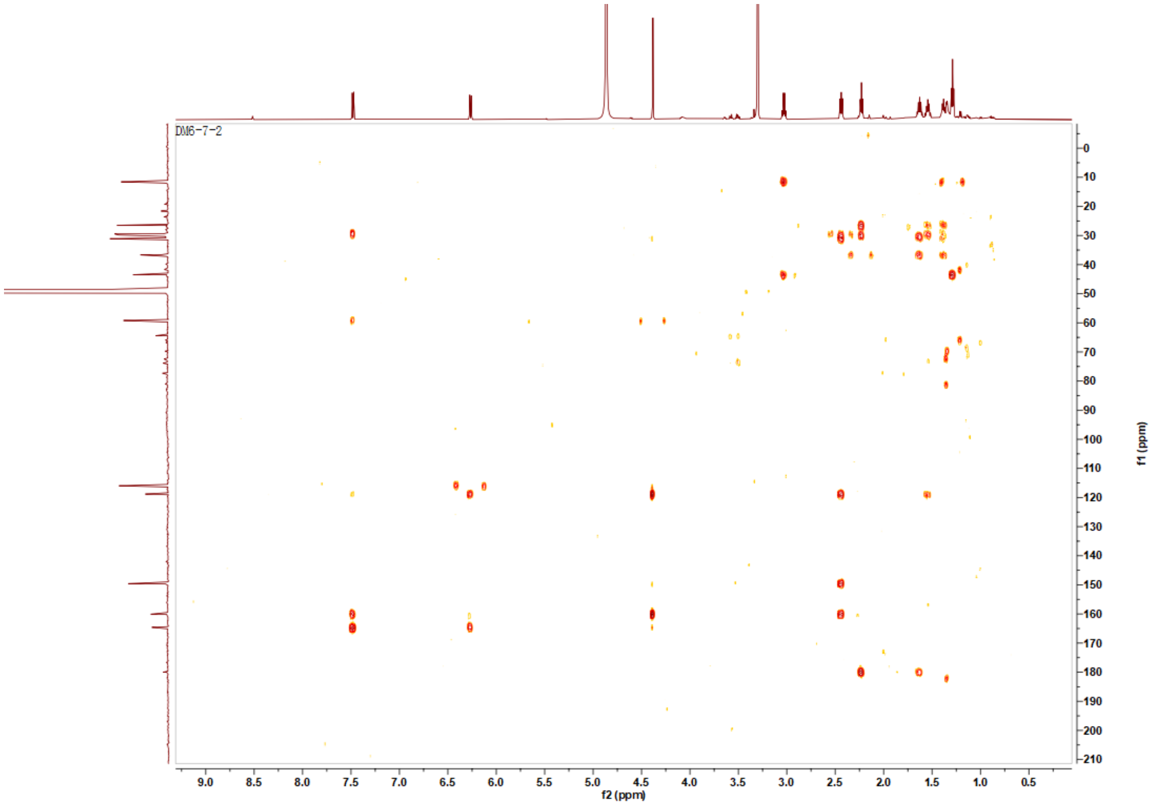

Figure S46: HMBC spectrum of compound 6

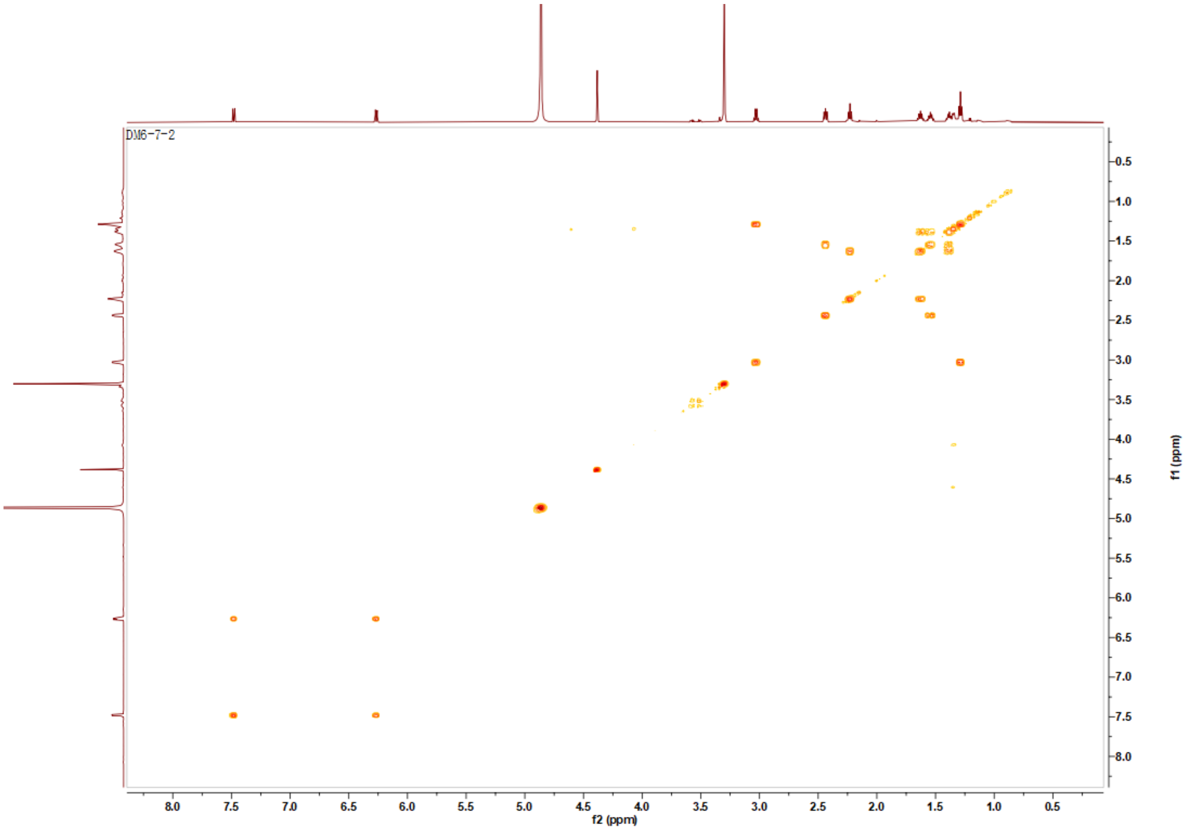

Figure S47: ^{1}H-^{1}H COSY spectrum of compound 6

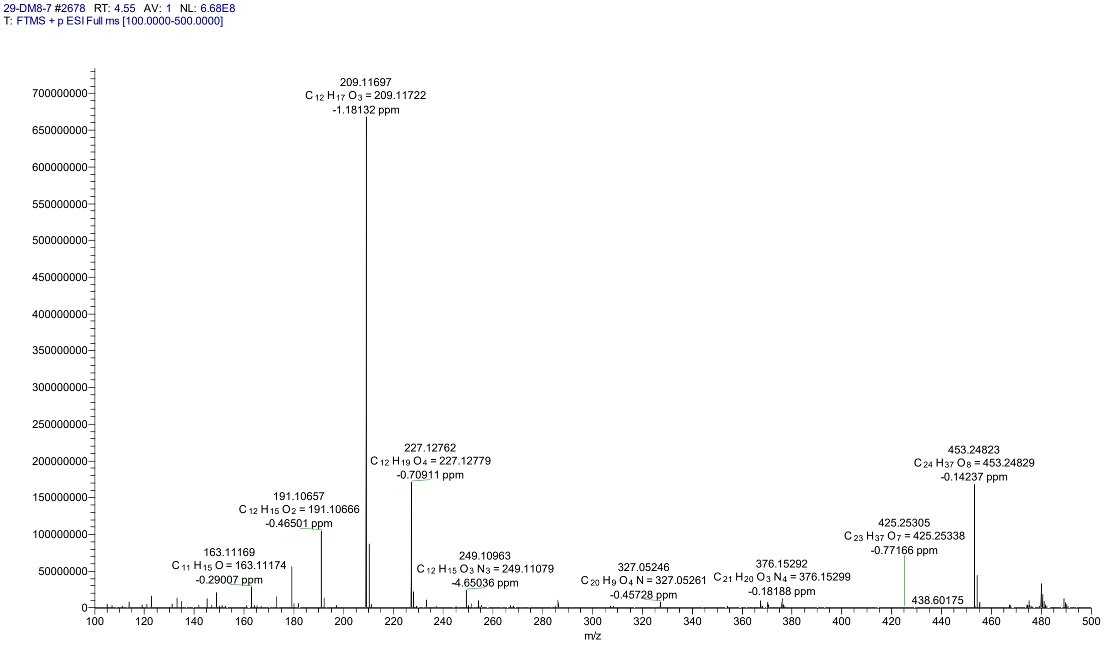

Figure S48: HRESIMS spectrum of compound 7

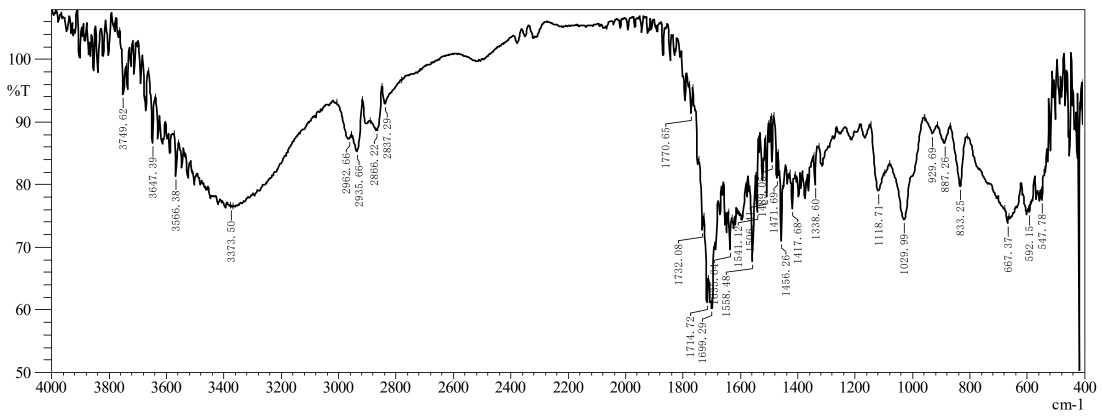

Figure S49: IR spectrum of compound 7

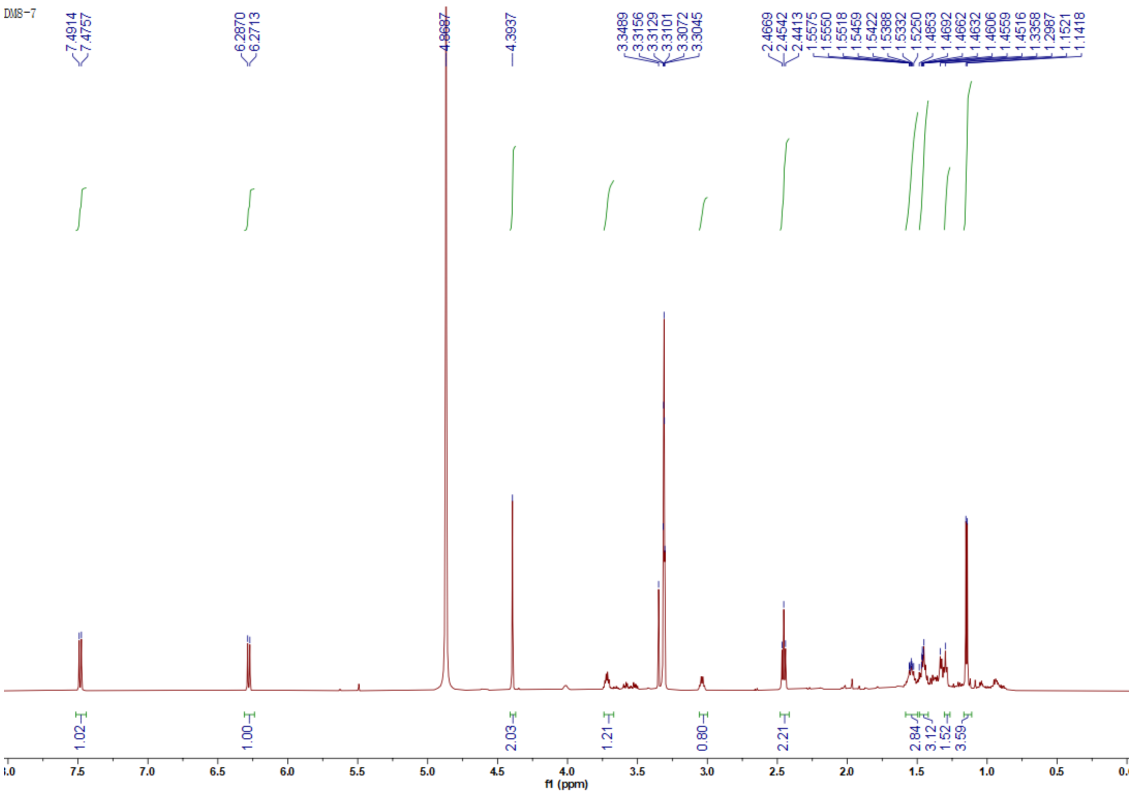

Figure S50: ^{1}H-NMR (600 MHz, CD_3OD) spectrum of compound 7

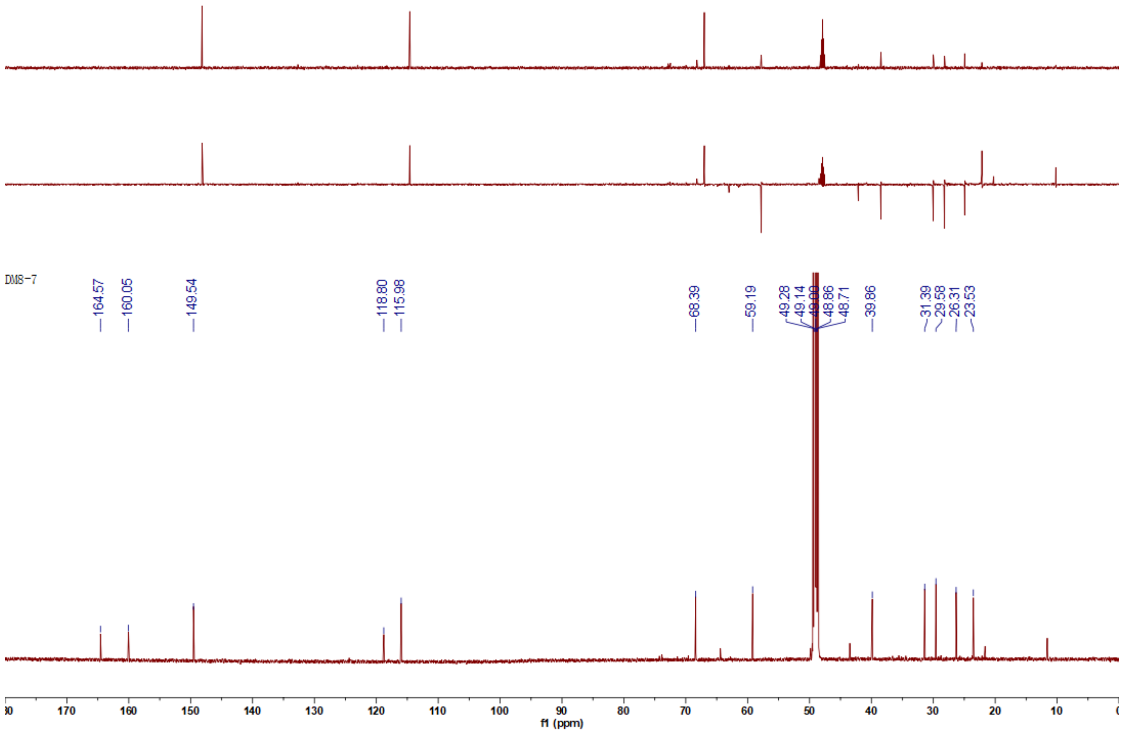

Figure S51: ^{13}C-NMR and DEPT (150 MHz, CD_3OD) spectrum of compound 7

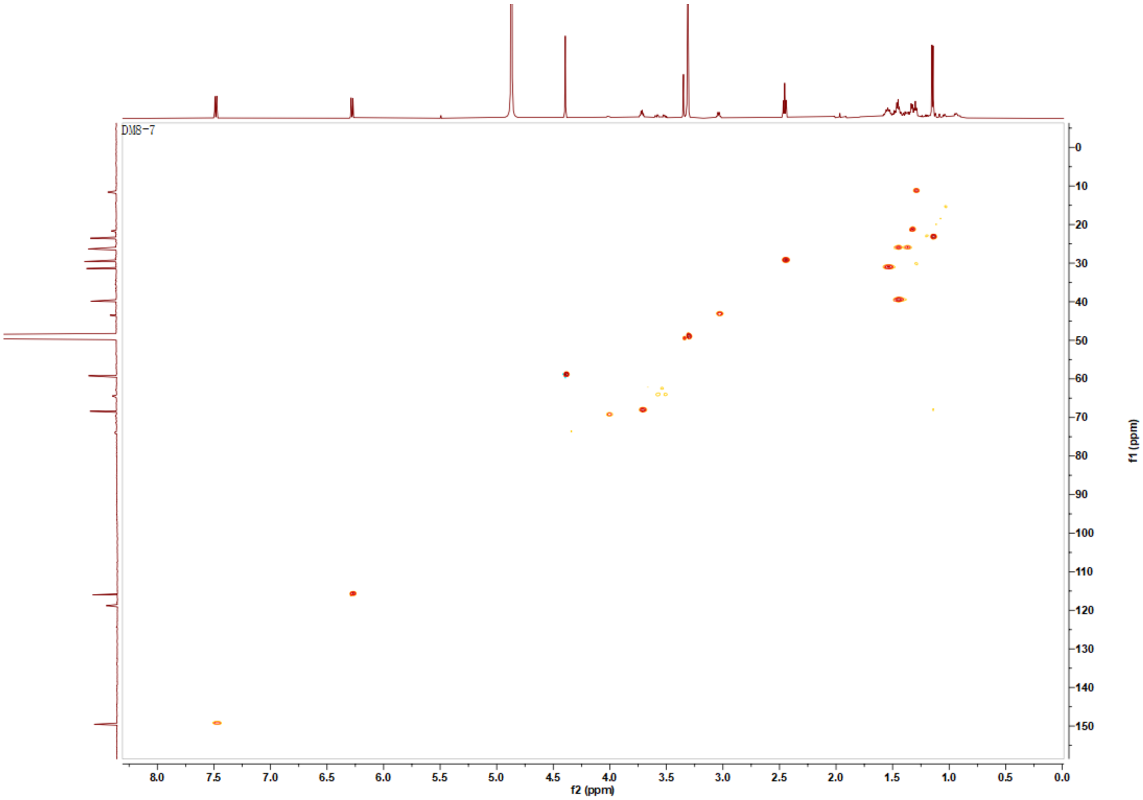

Figure S52: HSQC spectrum of compound 7

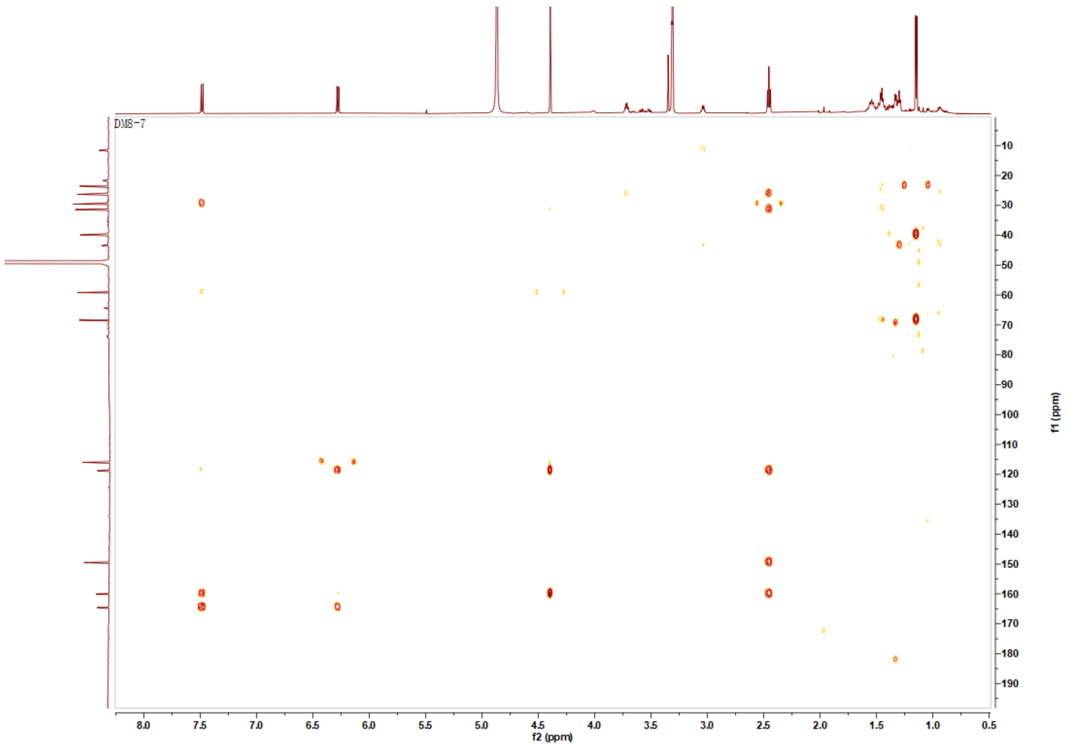

Figure S53: HMBC spectrum of compound 7

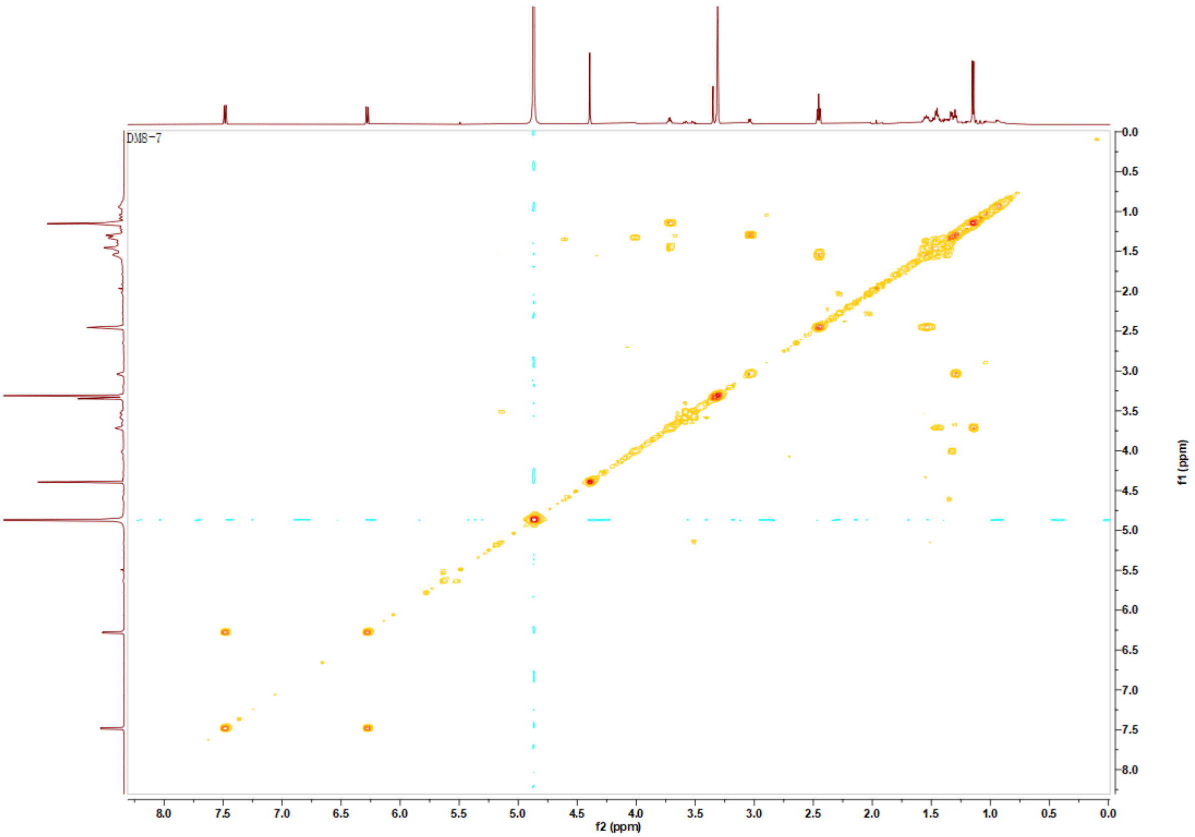

Figure S54: ^{1}H-^{1}H COSY spectrum of compound 7

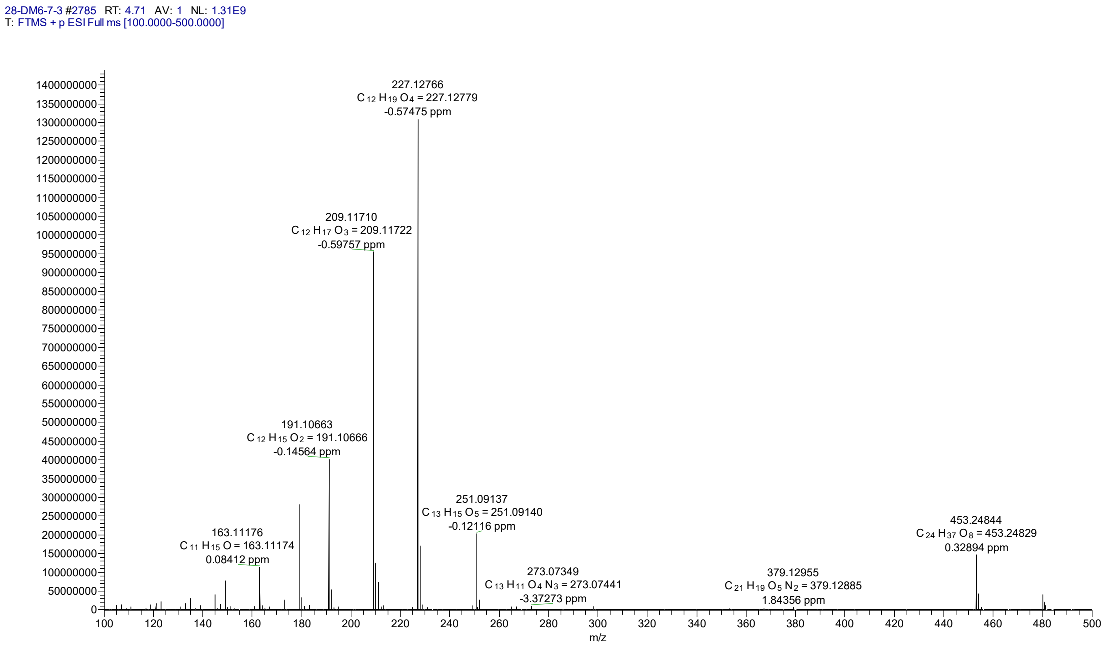

Figure S55: HRESIMS spectrum of compound 8

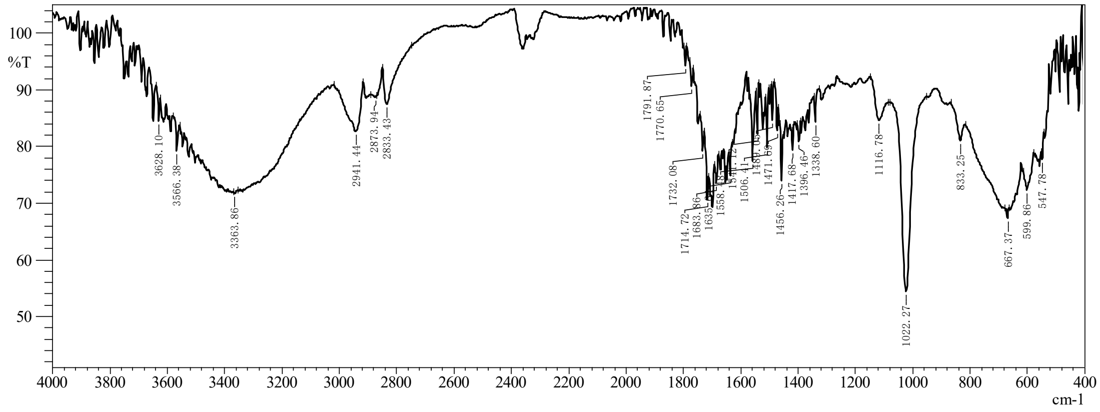

Figure S56: IR spectrum of compound 8

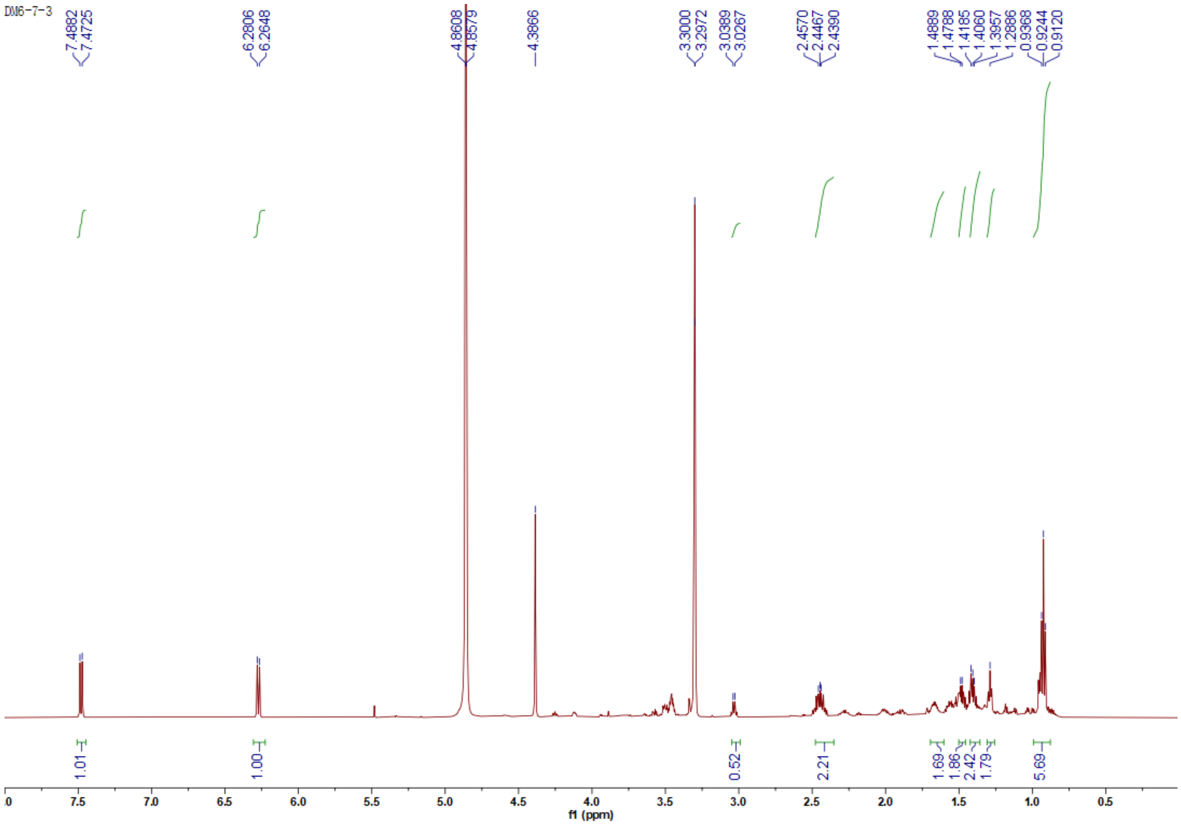

Figure S57: ^{1}H-NMR (600 MHz, CD3OD) spectrum of compound 8

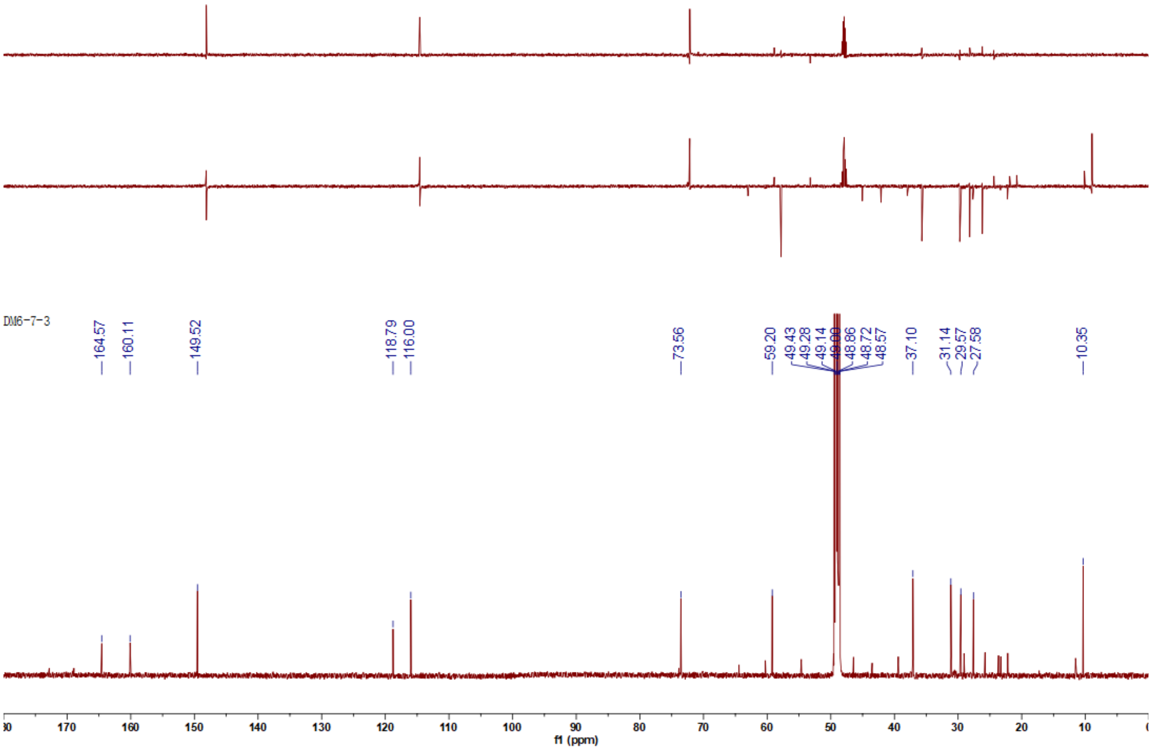

Figure S58: ^{13}C-NMR and DEPT (150 MHz, CD3OD) spectrum of compound 8

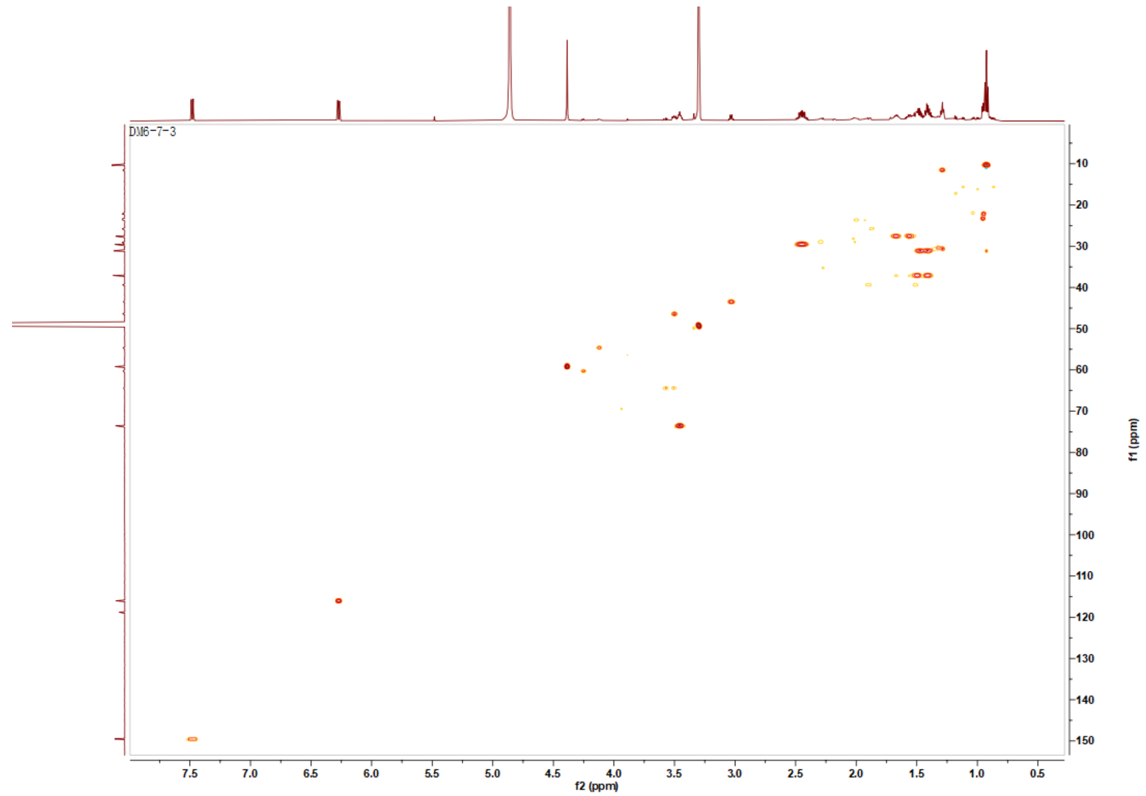

Figure S59: HSQC spectrum of compound 8

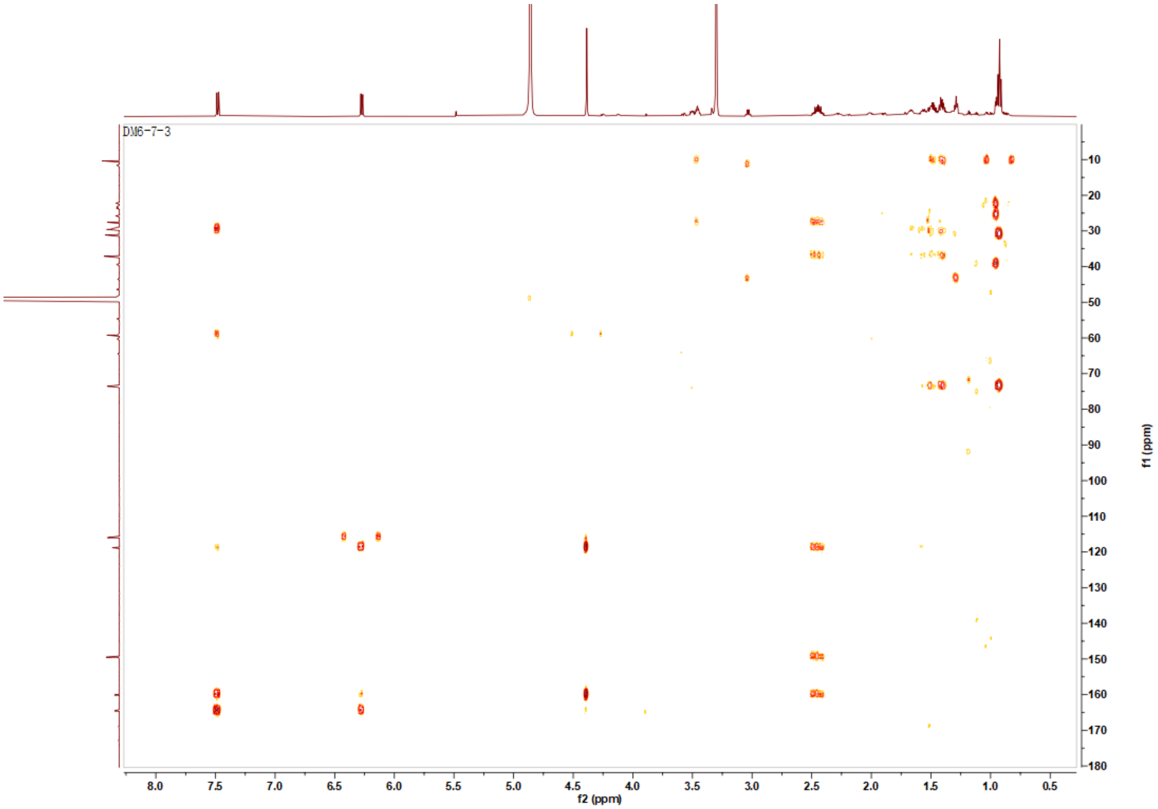

Figure S60: HMBC spectrum of compound 8

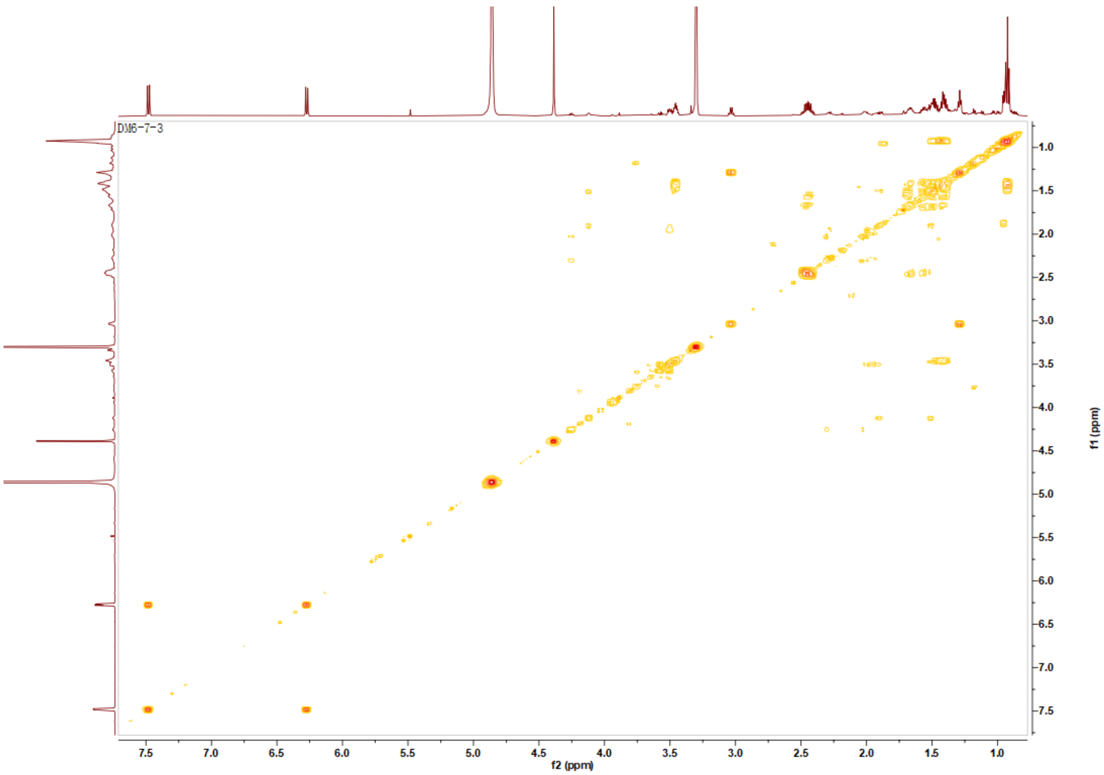

Figure S61: ^{1}H-^{1}H COSY spectrum of compound 8
